# LAceModule: Identification of Competing Endogenous RNA Modules by Integrating Dynamic Correlation

**DOI:** 10.3389/fgene.2020.00235

**Published:** 2020-03-18

**Authors:** Xiao Wen, Lin Gao, Yuxuan Hu

**Affiliations:** School of Computer Science and Technology, Xidian University, Xi'an, China

**Keywords:** ceRNA, microRNA, correlation, liquid association, modules

## Abstract

Competing endogenous RNAs (ceRNAs) regulate each other by competitively binding microRNAs they share. This is a vital post-transcriptional regulation mechanism and plays critical roles in physiological and pathological processes. Current computational methods for the identification of ceRNA pairs are mainly based on the correlation of the expression of ceRNA candidates and the number of shared microRNAs, without considering the sensitivity of the correlation to the expression levels of the shared microRNAs. To overcome this limitation, we introduced liquid association (LA), a dynamic correlation measure, which can evaluate the sensitivity of the correlation of ceRNAs to microRNAs, as an additional factor for the detection of ceRNAs. To this end, we firstly analyzed the effect of LA on detecting ceRNA pairs. Subsequently, we proposed an LA-based framework, termed LAceModule, to identify ceRNA modules by integrating the conventional Pearson correlation coefficient and dynamic correlation LA with multi-view non-negative matrix factorization. Using breast and liver cancer datasets, the experimental results demonstrated that LA is a useful measure in the detection of ceRNA pairs and modules. We found that the identified ceRNA modules play roles in cell adhesion, cell migration, and cell-cell communication. Furthermore, our results show that ceRNAs may represent potential drug targets and markers for the treatment and prognosis of cancer.

## Introduction

MicroRNAs (length: ~22 nt) are a kind of small non-coding RNAs (Yates et al., 2013). They can interact with Argonaute protein to form the RNA-induced silencing complex. This complex binds to target RNA sequences (termed microRNA response elements, MRE) with partial complementarity, influencing the stability of target RNAs (Bartel, 2009; Yates et al., 2013). Recent studies revealed that different RNAs with microRNA response elements that bind to the same microRNAs can regulate each other by competitively binding to the microRNAs they share. This is known as the competing endogenous RNA (ceRNA) model, and these RNAs are termed ceRNAs (Salmena et al., 2011). CeRNAs can be messenger RNAs (mRNAs), long non-coding RNAs (lncRNAs), pseudogene gene transcripts, and circular RNAs (circRNAs). The detection of ceRNA can be used to explain the function of thousands of uncharacterized non-coding RNAs, and is also considered the “Rosetta stone of a hidden RNA language” (Salmena et al., 2011; Thomson and Dinger, 2016). In addition, ceRNAs play critical roles in post-transcriptional regulation. Thus, they are involved in numerous physiological and pathological progresses, such as cancer (Salmena et al., 2011; Karreth and Pandolfi, 2013; Tay et al., 2014; Qi et al., 2015). For example, the tumor suppressor gene *PTEN* has been demonstrated to compete for microRNAs shared with other transcripts, such as *ZEB2, CNOT6L, VAPA, VCAN*, in many types of cancer (e.g., glioblastoma, melanoma, prostate cancer, and breast cancer) (Tay et al., 2014; Poliseno and Pandolfi, 2015). Of note, *PTENP1* (the pseudogene of *PTEN*), regulates the RNA levels of its cognate gene in prostate, melanoma, and clear-cell renal cell carcinoma (Poliseno et al., 2010, 2011; Johnsson et al., 2013; Yu et al., 2014). Furthermore, lncRNAs and circRNAs are also important regulators in the ceRNA model (Schmitt and Chang, 2016; Zhong et al., 2018). *HULC*, which is significantly upregulated in hepatocellular carcinoma, can reduce the expression and activity of miR-372 in liver cancer, which derepresses the translation of its target gene *PRKACB* and induces phosphorylation of the cAMP response element binding protein in liver cancer (Wang et al., 2010). CircHIPK3 inhibits the activity of mir-124 and promotes the expression of IL-6R by competitively binding to miR-124 (Zheng et al., 2016).

Owing to the large number of candidate ceRNA pairs and high cost of biological experiments, computational methods have become an efficient approach for the study of the ceRNA model (Le et al., 2017). For example, Zhou et al. (2014) constructed and investigated a ceRNA network in breast cancer; Sumazin et al. (2011) proposed a method based on conditional mutual information to infer candidate ceRNAs and analyze the ceRNA network in glioblastoma. Paci et al. (2014) used sensitivity correlation (SI) to infer the ceRNA network between lncRNAs and mRNAs in breast cancer. In that model, SI equals the difference between the Pearson correlation coefficient (PCC) and the partial correlation coefficient of ceRNAs with respect to their shared microRNAs. And List et al. (2019) further improved this method. All these methods predict ceRNA pairs based on two aspects: (1) ceRNAs should share a sufficient number of microRNAs, which can be evaluated through statistical tests, such as the hyper-geometric test (Salmena et al., 2011; Le et al., 2017); and (2) the expression of ceRNAs should be positively correlated, which can be estimated using the PCC (Chiu et al., 2015), SI (Paci et al., 2014; Do and Bozdag, 2018), or conditional mutual information (Sumazin et al., 2011). In addition, Zhang et al. (2018) proposed LncmiRSRN to construct lncRNA-mRNA ceRNA network via estimating the causal effects of lncRNAs on mRNAs with the IDA method. Besides studying ceRNAs using sequencing data, researchers also proposed mathematical models to simulate the ceRNA process, such as the minimal model (Figliuzzi et al., 2013), mass-action model (Ala et al., 2013), and coarse-grained model (Yuan et al., 2015). Recently, Wei et al. constructed a unified coarse-grained competition motif model and uncovered the complexity and generality of the molecular competition effect, including the ceRNA model (Wei et al., 2019). In that study, the investigators proposed that the strength of competition between ceRNAs is influenced by the abundance of ceRNAs and their target microRNAs (Wei et al., 2019). The strength of the correlation is maximized in the “R near-equimolar” regime, and gradually decreases with concentration of microRNAs away from the regime (Martirosyan et al., 2019; Wei et al., 2019). This means that the strength of ceRNA regulation varies based on the concentration of microRNAs. Those results also indicated that the strength of ceRNA regulation is sensitive to the expression levels of microRNAs around the proper concentration. Intuitively, two RNAs may be co-expressed due to some biological mechanisms. If they have an additional ceRNA relationship, their co-expression should be improved. When the levels of microRNA expression are low, the regulation between ceRNAs is not obvious. Furthermore, the ceRNA strength is sensitive to the expression levels of microRNAs. Hence, the strength of the co-expression should also be sensitive to these levels. We found the current studies using RNA sequencing data did not consider this characteristic of the ceRNA model. Hence, we propose that the sensitivity of co-expression to the expression levels of microRNAs they share may be a factor for predicting ceRNA relationships. We used dynamic correlation measure, termed liquid association (LA), to assess this sensitivity.

Unlike conventional correlations (e.g., PCC), dynamic correlation focuses on the change in the correlation of two variables following alterations in a third variable (Gunderson and Ho, 2014; Yu, 2018). For example, LA is defined as the mean of the derivative of the correlation between two objects with respect to a third condition (Li, 2002). LA has been used to identify disease candidate genes (Li et al., 2007) and human age-associated genes (Yang et al., 2018), as well as discover key microbial species and environmental factors of the microbial community (Ai et al., 2019).

LA is an appropriate measure for the evaluation of the correlation sensitivity of ceRNAs to microRNAs. In this study, we firstly analyzed the effectiveness of LA in detecting ceRNA pairs. Subsequently, we proposed a framework to investigate LA-based ceRNA modules (LAceModule) by integrating the conventional PCC and dynamic correlation LA with multi-view non-negative matrix factorization (NMF). By performing further analysis in breast cancer, we revealed that ceRNAs play roles in cell adhesion, cell migration, and cell-cell communication. Our results also showed that ceRNAs may represent promising drug targets and markers for the treatment and prognosis of cancer.

## Results

### LA for the Prediction of ceRNA Pairs

Current studies often use the PCC or SI to detect ceRNA pairs. This approach ignores the sensitivity of the correlation between RNAs to the expression levels of their shared microRNAs. To overcome this limitation, we used LA (Li, 2002) to measure the dynamic change of the correlation for a ceRNA pair depending on the expression levels of their shared microRNAs. Suppose that *EXP*_*R*1_ and *EXP*_*R*2_ represent the expression levels of two ceRNA candidates *R*1 and *R*2, respectively, while *EXP*_*MIC*_ denotes the sum of the expression levels of all their shared microRNAs, *MIC*. We normalized *EXP*_*R*1_ and *EXP*_*R*2_ using the z-scoring method such that *E*(*EXP*_*R*1_) = *E*(*EXP*_*R*2_) = 0, *Var*(*EXP*_*R*1_) = *Var*(*EXP*_*R*2_) = 1, where *E*(·)and *Var*(·) represent the expectation and variance of a random variable, respectively.

Supposing the above, the PCC between *R*1 and *R*2 is:

$$\begin{array}{l} \rho \left(R1,R2\right)=\frac{E\left[\left(EXP_{R1}-E\left(EXP_{R1}\right)\right)\times \left(EXP_{R2}-E\left(EXP_{R2}\right)\right)\right]}{\sqrt{Var\left(EXP_{R1}\right)\times Var\left(EXP_{R2}\right)}}\\ \;=E\left(EXP_{R1}\times EXP_{R2}\right). \end{array}$$

By conditioning, *E*(*EXP*_*R*1_ × *EXP*_*R*2_) = *E*(*E*(*EXP*_*R*1_ × *EXP*_*R*2_|*EXP*_*MIC*_)) = *E*(*g*(*EXP*_*MIC*_)), where *g*(*EXP*_*MIC*_) = *E*(*EXP*_*R*1_ × *EXP*_*R*2_|*EXP*_*MIC*_ = *exp*_*MIC*_) is the correlation between *R*1 and *R*2 when the expression level of the shared microRNA is *exp*_*MIC*_.

The LA of *R*1 and *R*2 with respect to their shared microRNAs is defined as $LA\left(R1,R2|MIC\right)=E\left(g^{\prime }\left(EXP_{MIC}\right)\right)$, where *g*(*EXP*_*MIC*_) = *E*(*EXP*_*R*1_ × *EXP*_*R*2_|*EXP*_*MIC*_ = *exp*_*MIC*_). According to the Stein *Lemma* (Stein, 1981), if the sum of the expression levels of all the shared microRNAs *MIC* follows the standard normal distribution, *LA*(*R*1, *R*2|*MIC*) = *E*(*EXP*_*R*1_ × *EXP*_*R*2_ × *EXP*_*MIC*_), the calculation of LA can be simplified as shown below:

$$\begin{array}{l} LA\left(R1,R2|MIC\right)=\frac{\overset{N}{\underset{i=1}{\sum }}EXP_{R1_{i}}\times EXP_{R2_{i}}\times EXP_{MIC_{i}}}{N} \end{array}$$

where *N* is the number of sample. We performed data transformation on *EXP*_*MIC*_ using the Van der Waerden's method to ensure that *EXP*_*MIC*_ follows the standard normal distribution. For *EXP*_*MIC*_1__, *EXP*_*MIC*_2__, ⋯, *EXP*_*MIC*_*N*__, we initially obtained their ranks *r*_1_, *r*_2_, ⋯, *r*_*N*_, and subsequently computed the transformed value as follows:

$$\begin{array}{l} EXP_{MIC_{1}}\text{=}\Phi^{-1}\left(\frac{r_{1}}{N+1}\right),EXP_{MIC_{2}}=\Phi^{-1}\left(\frac{r_{2}}{N+1}\right),\cdots \;,\\ EXP_{MIC_{N}}=\Phi^{-1}\left(\frac{r_{N}}{N+1}\right), \end{array}$$

where Φ(·) is the cumulative distribution function of the standard normal distribution.

**Figure 1:**
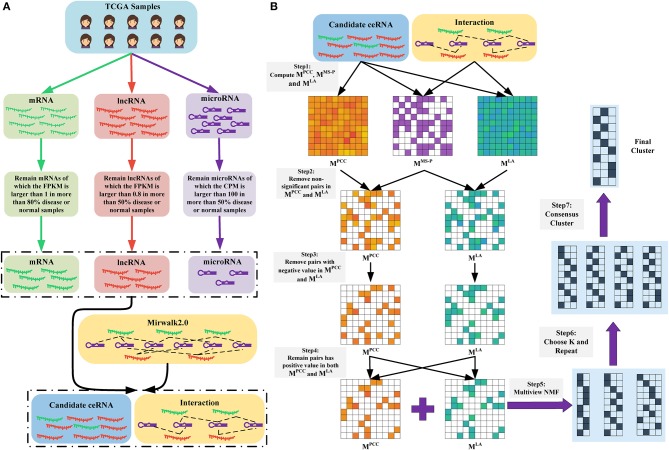
**(A)** Data preparation. We obtained the RNA-seq data of mRNAs and lncRNAs, as well as the microRNA-seq data of microRNAs. Subsequently, we removed non-expressed and lowly expressed RNAs. Finally, we retained RNAs that were presented in the RNA-microRNA interaction datasets (here is Mirwalk2.0) as candidate ceRNAs. **(B)** Overview of LAceModule. The inputs of LAceModule are candidate ceRNA expression profiles, microRNA expression profiles, and RNA-microRNA interactions. For each candidate ceRNA pair, the PCC value, LA value, and significance degree of shared microRNAs (MS-P) value can be obtained. For pairs with higher MS-P values (threshold is 0.05), negative PCC values or LA values should be removed (i.e., the PCC values and LA values of these pairs are set to zero). Multi-view NMF is executed using the PCC matrix, LA matrix, and different *K* as inputs. The best *K* is selected by comparing four clustering evaluation metrics. Subsequently, multi-view NMF procedures are repeated 10 times with the best *K* and different initial values. The final modules are obtained through consensus clustering of the repeat results.

**Figure 2:**
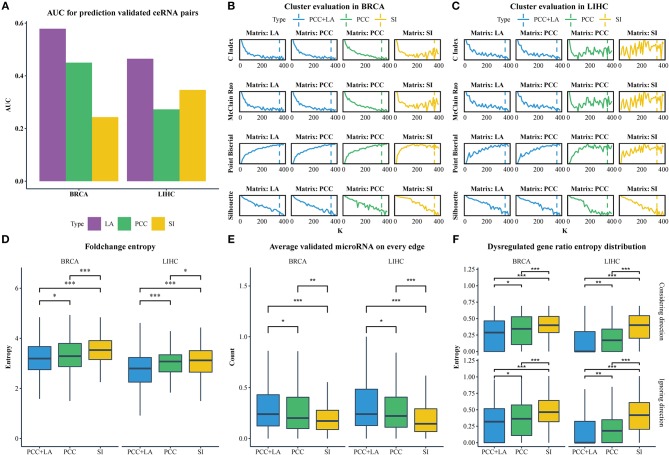
**(A)** The AUC value for predicting ceRNA pairs with LA, PCC, and SI in BRCA and LIHC. **(B)** Cluster evaluation of three methods on different matrices in BRCA. **(C)** Cluster evaluation of three methods on different matrices in LIHC. **(D)** Comparison of the gene fold-change entropy in modules between different clustering methods. **(E)** Comparison of the average validated microRNA of each pair in modules between different methods. **(F)** Comparison of the dispersion of dysregulated genes in modules between different methods. Top row: ignoring the direction of dysregulation, bottom row: considering the direction of dysregulation. (*p < 0.05; **p < 0.01; ***p < 0.001).

We downloaded the expression profiles of mRNAs, lncRNAs, and microRNAs of 1,072 and 365 patients with BRCA and LIHC, respectively, from The Cancer Genome Atlas (TCGA) to investigate the effect of LA on the prediction of ceRNA pairs. Non-expressed genes across all samples in a given type of cancer were removed (Figure 1A). Subsequently, we downloaded 2,667 experimentally validated ceRNA pairs from the LncCeRBase (Pian et al., 2018), miRSponge (Wang et al., 2015b), and lncACTdb (Wang et al., 2015a) databases. Considering the tissue-specific characteristics of ceRNAs and gene symbol mapping, seven validated ceRNA pairs in breast cancer and six validated ceRNA pairs in liver cancer were obtained (Table 1). All pairs in our candidate ceRNAs set significantly shared microRNAs (hypergeometric test, *p* < 0.05). For each disease, we considered these ceRNA pairs as benchmarks and all pairs, which were consisted of two genes in benchmarks and had sufficient number of shared microRNAs, as candidate pairs. We use these two datasets to evaluate the performance of LA-, PCC-, and SI-based methods, respectively. In breast cancer, the area under curve (AUC) values of the LA, PCC, and SI approaches were 0.58, 0.45, and 0.24, respectively. In liver cancer, these AUC values were 0.46, 0.27, and 0.35, respectively (Figure 2A). PCC and SI are usually used to identify ceRNA pairs, and our results indicate that LA also has the ability to predict ceRNA pairs.

**Table 1:** LA, PCC, and SI values of validated ceRNA pairs.

**ceRNA1**	**ceRNA2**	**PCC**	**LA**	**SI**	MS-P^*^	**Disease**
ENSG00000234741	ENSG00000171862	−0.058	0.040	−0.008	0.005	BRCA
ENSG00000251562	ENSG00000070831	0.043	−0.009	0.002	0.001	BRCA
ENSG00000251562	ENSG00000135446	−0.377	0.000	−0.003	0.022	BRCA
ENSG00000115414	ENSG00000026508	0.082	−0.003	−0.001	0.001	BRCA
ENSG00000108821	ENSG00000026508	−0.014	0.082	0.001	0.029	BRCA
ENSG00000171862	ENSG00000038427	0.379	0.075	−0.004	0.002	BRCA
ENSG00000038427	ENSG00000139687	0.368	0.058	0.000	0.003	BRCA
ENSG00000226950	ENSG00000168036	0.131	0.103	−0.003	0.012	LIHC
ENSG00000234741	ENSG00000150593	0.205	−0.205	−0.014	0.003	LIHC
ENSG00000234741	ENSG00000171862	−0.003	−0.107	−0.002	0.013	LIHC
ENSG00000241388	ENSG00000057663	0.035	−0.068	−0.005	0.033	LIHC
ENSG00000251164	ENSG00000148516	−0.093	0.097	−0.001	0.004	LIHC
ENSG00000251164	ENSG00000168615	−0.392	0.411	0.003	0.034	LIHC

* *MS-P, statistical significance of shared microRNAs between RNAs*.

### Identification of ceRNA Modules Using the LAceModule

We proposed the LAceModule (Figure 1B), a framework based on multi-view NMF (Liu et al., 2013) to systematically identify ceRNA modules using LA. For each candidate ceRNA pair, we computed the PCC value, LA value, and the degree of significance of shared microRNAs (MS-P) (see section Materials and Methods) to construct three matrices *M*^*PCC*^, *M*^*LA*^, and *M*^*MS*−*P*^, respectively. Subsequently, when the MS-P values of candidate ceRNA pairs were ≥0.05, we set their corresponding PCC values and LA values to zero. Owing to the non-negativity requirement in the multi-view NMF framework, we set negative values in *M*^*PCC*^ and *M*^*LA*^ to zero. Considering that a ceRNA pair should be co-expressed and sensitive to change in the expression of their shared microRNAs, we set the values in the same entry of *M*^*PCC*^ and *M*^*LA*^ of candidate ceRNA pairs to zero if either of these values was zero. Finally, we integrated *M*^*PCC*^ and *M*^*LA*^ using multi-view NMF to identify ceRNA modules.

For multi-view NMF, there are two observation views *M* = {*M*^*PCC*^, *M*^*LA*^}, each of which is a *G* × *G* non-negative matrix, where *G*is the number of candidate ceRNAs. Each matrix in *M*, *M*^*v*^ ∈ {*M*^*PCC*^, *M*^*LA*^}, can be factorized to $U_{G\times K}^{v}\geq 0$ and ${\left(V_{G\times K}^{v}\right)}^{T}\geq 0$ that *M*^*v*^ ≈ *U*^*v*^(*V*^*v*^)*T* and each row of (*V*^*v*^)*T* can be considered as the *K*-rank representation of the corresponding candidate ceRNA point. Here, we attempted to identify a low-rank representation that is suitable for both views, which is defined as (V^*^)*T*. We factorized each matrix in *M* and made each (*V*^*v*^)*T* as close as possible to (V^*^)*T*. Therefore, we determined the objective function as follows:

$$\begin{array}{l} \min({\left\|M^{PCC}-U^{PCC}{\left(V^{PCC}\right)}^{T}\right\|}_{F}^{2}+{\left\|M^{LA}-U^{LA}{\left(V^{LA}\right)}^{T}\right\|}_{F}^{2}\\ +\lambda_{PCC}{\left\|V^{PCC}-V^{*}\right\|}_{F}^{2}+\lambda_{LA}{\left\|V^{LA}-V^{*}\right\|}_{F}^{2})\\ \text{s}.\text{t}.\;\forall 1\leq k\leq K,{\left\|U_{\cdot ,k}^{PCC}\right\|}_{1}=1,{\left\|U_{\cdot ,k}^{LA}\right\|}_{1}\\ =1\text{ and }U^{PCC},U^{LA},V^{PCC},V^{LA},V^{*}\geq 0 \end{array}$$

where λ_*PCC*_ and λ_*LA*_ tunes the relative weight among different views and between standard NMF error and disagreement among (V^*^)*T*, (*V*^*PCC*^)*T*, and (*V*^*LA*^)*T*. We used an iterative procedure by updating one variable, while maintaining the remaining variables fixed to solve this optimization problem (see details in Materials and Methods section). After computing the (V^*^)*T*, we obtained the module label of RNA *i* using $arg\max_{j=1,2,\cdots \;,K}V_{ij}^{*}$.

Of note, the LAceModule requires pre-determination of the number of modules, *K*. We evaluated the clustering performance to select an optimal *K* ranging from 10 to 400 with an increment of 10 by considering four metrics (Figures 2B,C), namely the C-index (Hubert and Schultz, 1976), McClain-Rao (McClain and Rao, 1975), point biserial correlation coefficient (Milligan, 1981), and silhouette coefficient (Rousseeuw, 1987). By simultaneously considering four metrics on two matrices, we selected *K* = 360 in BRCA and *K* = 370 in LIHC. To obtain robust ceRNA modules, the LAceModule repeated the multi-view NMF procedures 10 times, and computed a consensus matrix to identify ceRNA modules using the cluster-based similarity partitioning algorithm (CSPA) (Strehl and Ghosh, 2003). Specifically, CSPA generates a binary matrix for each result of the multi-view NMF clustering, with “1” representing two associated genes in the same cluster and “0” for not. The consensus matrix is the sum of these binary matrices. ceRNA modules can be identified through spectral clustering on this consensus matrix using the optimal *K* selected above.

### Comparison Between the LAceModule and PCC/SI-Based Methods

We used NMF to replace the multi-view NMF and the PCC matrix or SI matrix as input to compare the performance of conventional and dynamic correlations in the detection of ceRNA modules. In the PCC matrix and SI matrix, negative values or the corresponding MS-P values ≥0.05 were set to zero. We also tested *K* ranging 10–400, with an increment of 10, and evaluated the clustering performance with the same indicators mentioned in Section Identification of ceRNA modules using the LAceModule. We selected *K*s equal to 350 and 360 for PCC-based and SI-based results in BRCA, respectively, while *K*s equal to 360 and 340, respectively, were selected for LIHC (Figures 2B,C). In the following sections, we used “PCC+LA” to represent the modules detected by the LAceModule, as well as “PCC” and “SI” to represent the modules based on PCC or SI, respectively.

CeRNA pairs are highly co-expressed; hence, the fold change of gene expression in a ceRNA module tends to be similar in the disease and normal states. We analyzed the differential expression of genes between normal tissues and tumor tissues using the R packages edgeR (Robinson et al., 2010) (Materials and Methods section) to obtain the fold change. Subsequently, we calculated the entropy of fold change for each module. As shown in Figure 2D, the entropy distribution of “PCC+LA” was significantly lower than those of “PCC” (FDR = 0.0476 in BRCA, FDR = 5.12E-06 in LIHC; Kolmogorov–Smirnov one-tailed test) and “SI” (FDR = 8.16E-09 in BRCA, FDR = 5.12E-06 in LIHC; Kolmogorov–Smirnov one-tailed test). By comparing “PCC” and “SI,” we also found that the former was significantly lower than the latter (FDR = 7.20E-05 in BRCA, FDR = 4.76E-02 in LIHC; Kolmogorov–Smirnov one-tailed test). Similarly, if a gene in a ceRNA pair is dysregulated, the other gene also tends to be dysregulated. In addition, the direction of the dysregulation also tends to be the same. This indicates that, when a gene in a ceRNA pair is upregulated, the other gene also tends to be upregulated. Therefore, we calculated the entropy of the dysregulated gene ratio and the entropy of the different dysregulation direction ratio of each module. The results are shown in Figure 2F; the top row illustrates the dysregulated gene ratio without direction, while the bottom row shows the dysregulated gene ratio with direction. The results showed that the modules of “PCC+LA” were significantly lower than those of “PCC” (top: FDR = 0.0246 in BRCA, FDR = 6.50E-03 in LIHC; bottom: FDR = 2.62E-02 in BRCA, FDR = 7.82E-03 in LIHC; Wilcoxon one-tailed test) and “SI” (top: FDR = 5.82E-12 in BRCA, FDR = 3.47E-24 in LIHC; bottom: FDR = 6.88E-10 in BRCA, FDR = 2.59E-23 in LIHC; Wilcoxon one-tailed test) in both situations. For “PCC” and “SI”, the former performed better than the latter in two situations (top: FDR = 5.39E-06 in BRCA, FDR = 9.15E-16 in LIHC; bottom: FDR = 2.39E-04 in BRCA, FDR = 2.09E-15 in LIHC; Wilcoxon one-tailed test).

CeRNAs are regulated through shared microRNAs. Therefore, ceRNA modules may tend to share more microRNAs in each pair. We used experimentally validated mRNA-microRNA interaction in miRTarBase (Chou et al., 2016) to evaluate the average number of shared microRNAs in a pair. The results are shown in Figure 2E. The modules of “PCC+LA” shared more microRNAs on average than those of “PCC” (FDR = 1.84E-02 in BRCA, FDR = 1.84E-02 in LIHC; Wilcoxon one-tailed test) and “SI” (FDR = 1.05E-06 in BRCA, FDR = 2.62E-09 in LIHC; Wilcoxon one-tailed test). Moreover, the modules of “PCC” shared more microRNAs on average than those of “SI” (FDR = 8.46E-03 in BRCA, FDR = 3.82E-05 in LIHC; Wilcoxon one-tailed test).

Collectively, the comparisons of gene fold change, gene dysregulation ratio, and number of shared microRNAs suggest that the integration of conventional and dynamic correlations offers better detection of ceRNA modules than conventional correlation alone.

### Functional Analysis of ceRNA Modules in Breast Cancer

**Figure 3:**
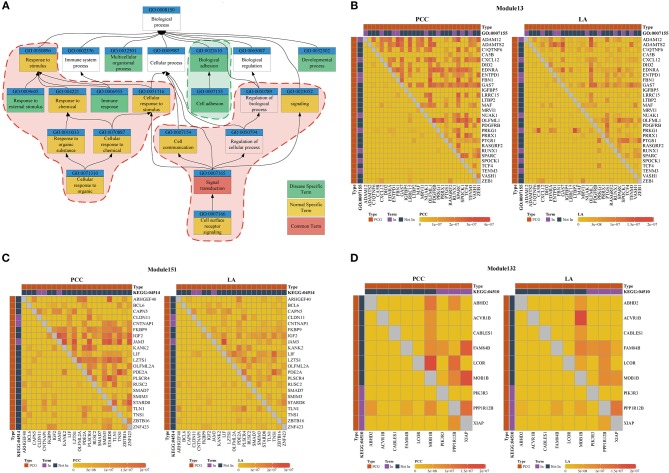
**(A)** Top five most frequently enriched terms in disease and normal states. Red areas are considered common functions in disease and normal states. Green areas are considered disease-specific functions. **(B–D)** Heatmap of PCC and LA values in disease and normal states (top right: disease state, bottom left: normal state). **(B)** Module 13 enriched for GO:0007155 **(C)** Module 151 enriched for KEGG:045142. **(D)** Module 132 enriched for KEGG:04510. (PCG: protein-coding gene).

We used the results of the BRCA dataset for further analysis. For investigating the difference in ceRNA relationship between tumor and normal states, we identified 348 ceRNA modules in breast tumor tissues and 314 modules in normal breast tissues using LAceModule. We used the g:Profiler (Reimand et al., 2007) for function enrichment of ceRNA modules in the disease and normal states. In Table 2, we listed the top five most frequently enriched Gene Ontology (GO) terms in both states. We analyzed the relationship of these GO terms (Figure 3A) and found that the ceRNA modules are associated with cell adhesion (GO:0007155) specifically in disease tissues, compared with the modules of the normal tissues. It is established that activation of invasion and metastasis is an important hallmark of cancer (Hanahan and Weinberg, 2011). Current research studies suggest that the loss of cell adhesion is strongly associated with tumor invasion and metastasis (Cavallaro and Christofori, 2001; Okegawa et al., 2004). This term and its parent term biological adhesion (GO:0022610) are enriched in 15 modules. In most of these modules, gene pairs have larger PCC and LA values in diseased cells than in normal cells (Figure 3B, Supplementary File 1). Similarly, the most frequently and specifically enriched Kyoto Encyclopedia of Genes and Genomes (KEGG) pathways (Supplementary Table 1) in the disease state were cell adhesion molecules (CAMs) (KEGG:004514) and focal adhesion (KEGG:04510), which play a pivotal role in tumor metastasis and cell migration (Ridley et al., 2003; Okegawa et al., 2004; Kim and Wirtz, 2013). In most of these modules, gene pairs also have larger PCC and LA values in diseased cells than in normal cells (Figures 3C,D, Supplementary Files 2, 3). These results suggest that ceRNAs play important roles in the invasion and metastasis of breast tumor cells, as previously indicated (Yang et al., 2014; Hu et al., 2017; Zheng et al., 2018).

**Table 2:** Most frequently enriched GO terms.

**Term ID**	**Domain**	**Term name**	**Count**
**Disease state**			
GO:0007155^1^	BP	Cell adhesion	15
GO:0022610^1^	BP	Biological adhesion	15
GO:0032501^1^	BP	Multicellular organismal process	13
GO:0006955^1^	BP	Immune response	11
GO:0007165^3^	BP	Signal transduction	11
GO:0009605^1^	BP	Response to external stimulus	11
GO:0032502^1^	BP	Developmental process	11
**Normal state**			
GO:0010033^2^	BP	Response to organic substance	8
GO:0070887^2^	BP	Cellular response to chemical stimulus	8
GO:0071310^2^	BP	Cellular response to organic substance	8
GO:0007154^2^	BP	Cell communication	7
GO:0007165^3^	BP	Signal transduction	7
GO:0007166^2^	BP	Cell surface receptor signaling pathway	7
GO:0023052^2^	BP	Signaling	7
GO:0042221^2^	BP	Response to chemical	7
GO:0050896^2^	BP	Response to stimulus	7
GO:0051716^2^	BP	Cellular response to stimulus	7

1 *Disease state-specific GO terms*,

2 normal state-specific GO terms, and

3 *common GO terms in both disease state and normal state*.

**Figure 4:**
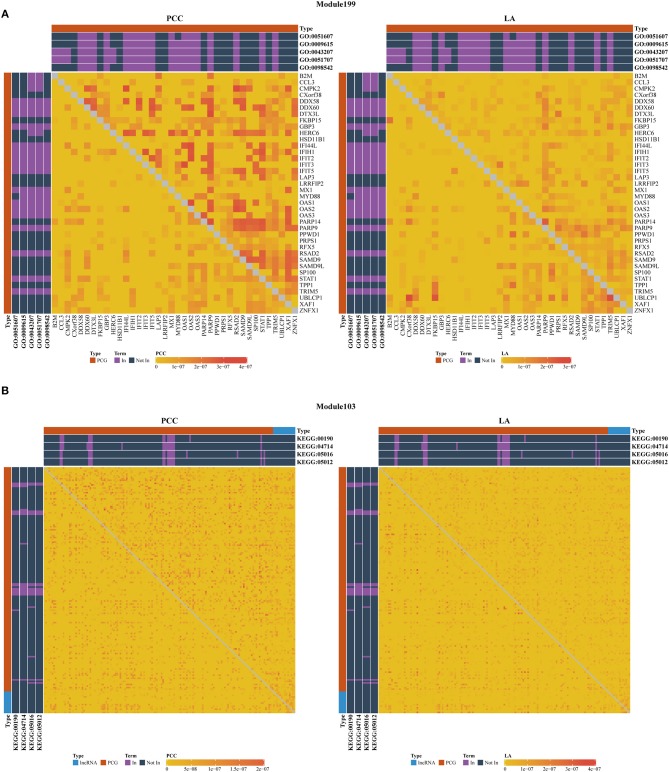
Heatmap of PCC and LA values with most significant GO **(A)** and KEGG **(B)** terms in disease and normal states (top right: disease state, bottom left: normal state, PCG: protein-coding gene, lncRNA: long-non-coding RNA).

As shown in Table 3, we also investigated the most significant terms. The most significant GO terms were obtained from Module 199, and were associated with defense against other organisms, such as viruses. Numerous studies have indicated that the mouse mammary tumor virus, bovine leukemia virus, human papillomavirus, and Epstein–Barr virus are associated with breast cancer (Amarante and Watanabe, 2009; Alibek et al., 2013; Lawson et al., 2018). As shown in Figure 4A, genes in this module exhibit larger PCC and LA values. For the KEGG dataset, the most significant pathways were obtained from Module 103 (Figure 4B); these pathways were significantly enriched for oxidative phosphorylation, thermogenesis, Huntington's disease, and Parkinson's disease.

**Table 3:** Most significant GO terms and KEGG pathways.

**Term ID**	**Domain**	**Term name**	**FDR**	**Precision**	**Module ID**
GO:0051607	BP	Defense response to virus	3.81E-19	0.459	Module 199
GO:0009615	BP	Response to virus	1.70E-18	0.486	Module 199
GO:0043207	BP	Response to external biotic stimulus	8.96E-18	0.595	Module 199
GO:0051707	BP	Response to other organism	8.96E-18	0.595	Module 199
GO:0098542	BP	Defense response to other organism	8.96E-18	0.486	Module 199
KEGG:00190	KEGG	Oxidative phosphorylation	3.07E-09	0.217	Module 103
KEGG:03010	KEGG	Ribosome	2.30E-08	0.2	Module 228
KEGG:04714	KEGG	Thermogenesis	2.33E-08	0.246	Module 103
KEGG:05016	KEGG	Huntington's disease	2.33E-08	0.232	Module 103
KEGG:05012	KEGG	Parkinson's disease	2.33E-08	0.203	Module 103

Collectively, comparison of the functions of ceRNA modules in breast tumor and normal tissues suggested that the ceRNA relationship may rewire in different cell states and ceRNA may exert an effect on the development or progression of breast cancer.

### ceRNA Modules Are Associated With Aberrant Genetic and Epigenetic Regions in Breast Cancer

**Figure 5:**
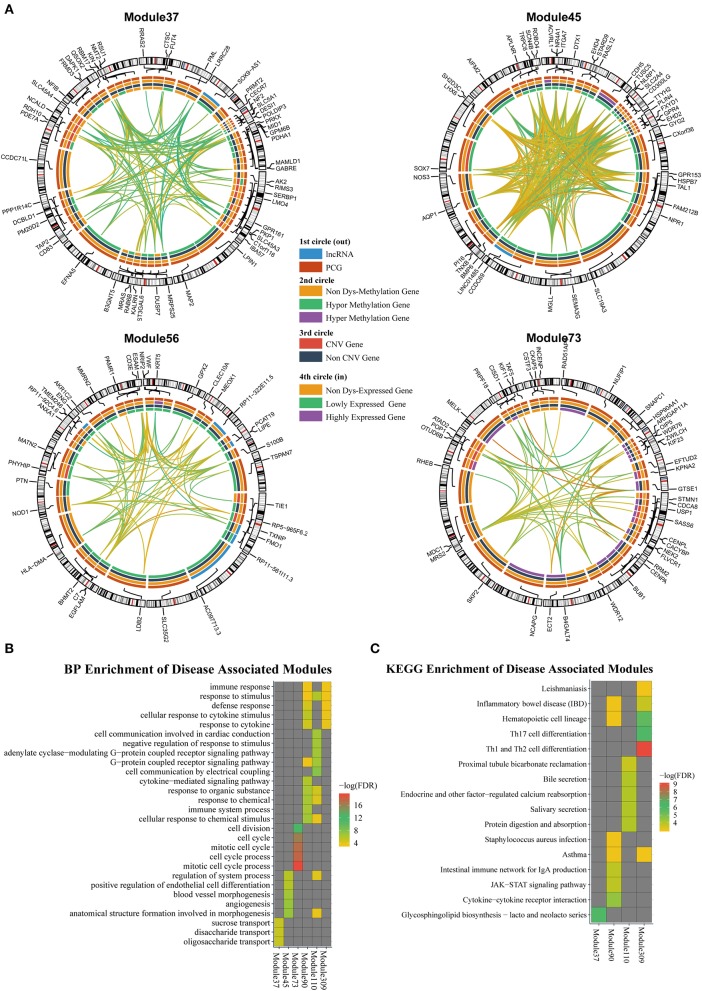
**(A)** The modules that are enriched for breast invasive carcinoma (BRCA)-associated genes. **(B)** GO enriched terms of BRCA-associated modules. **(C)** KEGG enriched terms of BRCA-associated modules.

According to the methods described in Materials and Methods section, we obtained 1,829 dysregulated mRNAs, 264 dysregulated lncRNAs (Supplementary File 4, Supplementary Table 2), 1,074 CNV genes, and 51 differentially methylated genes. We considered these genes as BRCA-associated genes. We identified BRCA-associated modules that are enriched for both expression-dysregulated genes (*p* < 0.01); and genes associated with aberrant CNV or DNA methylation (*p* < 0.05). Totally, we obtained eight BRCA associated modules (Table 4, Figure 5A, Supplementary File 5). The top five most significant enriched functions and pathways of these modules are shown in Figures 5B,C. We found that six of eight modules are significantly enriched for breast cancer-associated functions and pathways, such as immune response (Module 90 and Module 309) (Bates et al., 2018), cell communication (Module 110) (Brooks and Wicha, 2015), cell cycle (Module 73) (Otto and Sicinski, 2017), blood vessel morphogenesis (Module 45) (Kakolyris et al., 2000), and sucrose process (Module 37) (Jiang et al., 2016).

**Table 4:** BRCA-associated ceRNA modules.

**Module ID**	**Differentially expressed gene *p*-value**	**Methylation gene *p*-value**	**CNV gene *p*-value**	**LncRNA count**
Module 37	1.26E-5^***^	0.20	4.7E-2^*^	2
Module 45	0^***^	1.3E-2^*^	0.89	1
Module 56	0^***^	9.7E-3^**^	0.62	6
Module 73	8.86E-7^***^	1	2.6E-2^*^	0
Module 90	0^***^	2.2E-3^**^	0.81	2
Module 110	0^***^	0.16	3.3E-3^**^	0
Module 128	2.24E-6^*^	2.1E-2^*^	0.51	0
Module 309	0^***^	2.1E-2^*^	0.96	7

Level of significance:

* *p < 0.05*;

** *p < 0.01*;

*** *p < 0.001*.

We also investigated the relationship between lncRNAs and oncogenes in these modules. We downloaded the oncogene dataset from ONGENE (Liu et al., 2017). We found that in Module 45, *LINC01485* was correlated with *AQP1*, with PCC and LA values of 0.397 and 0.087, respectively. *AQP1* is related to tumor cell migration, invasion, and angiogenesis (Tomita et al., 2017). In Module 309, lncRNA *HSPC324* (ENSG00000228401) was highly co-expressed with oncogene *KLF2*, with PCC and LA values of 0.642 and 0.012, respectively. *KLF2* is a tumor suppressor gene, which inhibits cell growth, migration, and invasion in numerous types of cancer, such as colorectal cancer (Wang et al., 2017), breast cancer (Zhang et al., 2015), and prostate cancer (Wang et al., 2019). Our results indicate that these lncRNAs may be new drug targets for cancer therapy.

### ceRNA Modules Predict Survival in Patients With Breast Cancer

**Figure 6:**
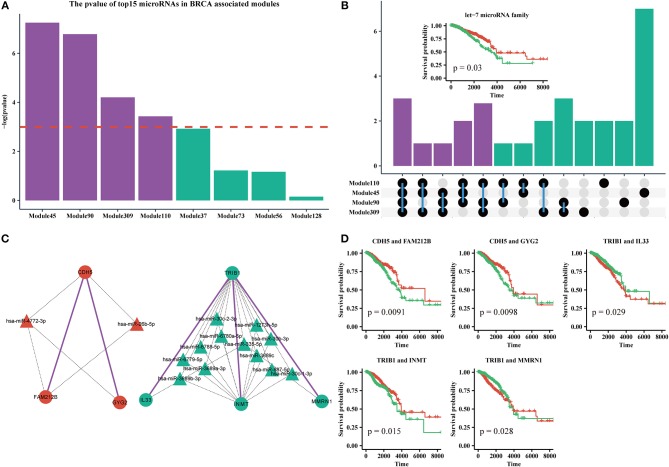
**(A)** The log-rank test p-values of the top15 most frequently shared microRNAs in modules enriched for BRCA-associated genes; the red dashed line indicates the level of –log(0.05). **(B)** Upset plot of microRNAs in Module 45, 90, 110, and 309. Subplot: the Kaplan-Meier curves of five microRNAs from the let-7 family. **(C)** The ceRNA pair markers for BRCA therapy and prognosis and their target microRNAs in miRTarBase. **(D)** Kaplan–Meier curves of ceRNA pair markers.

MicroRNAs are clinically important in cancer. Therefore, we used the top 15 most commonly shared microRNAs in BRCA-associated modules to analyze their relationship with patient outcome. We used a k-means algorithm to classify patients into two groups based on the expression of the top 15 microRNAs in each module, and performed a Kaplan–Meier analysis. We found that half of the top 15 microRNAs sets in Module 45, Module 110, Module 90, and Module 309, could significantly distinguish patients (Figure 6A). By analyzing the microRNAs in these modules, we found that 10 of 30 microRNAs were shared by more than three modules (Figure 6B, Supplementary Table 3). In addition, half of those 10 microRNAs were derived from the *let-7* microRNA family. Many studies have suggested that the *let-7* family participates in the process of metastasis and resistance to therapy in breast cancer (Cunningham et al., 2010; Chiu et al., 2014). Our results also demonstrate that the *let-7* microRNA family can be a prognostic marker of breast cancer (Figure 6B, Supplementary File 9).

In addition, we identified five ceRNA pairs (i.e., *CDH5* with *FAM212B, CDH5* with *GYG2* in Module 45, *TRIB1* with *IL33, TRIB1* with *INMT*, and *TRIB1* with *MMRN1* in Module 110) that can be used as prognostic markers of breast cancer in BRCA-associated modules (Figures 6C,D, Supplementary File 9). In our dataset, *CDH5* is a hyper-methylated gene (six of eight sites are hypermethylated in the promoter region), while *TRIB1* is duplicated or amplified in >14% of patients with BRCA. Recent studies showed that *CDH5* levels and *CDH5* glycosylation are biomarkers for metastatic breast cancer (Fry et al., 2016) and *TRIB1* plays a critical role in cell cycle and survival via NF-κB signaling (Gendelman et al., 2017). Interestingly, we found that these genes cannot individually act as prognostic markers (Supplementary File 6). However, the gene sets of the ceRNA pairs and their experimental validated shared microRNAs in miRTarBase are effective markers for therapy and prognosis, indicating that these ceRNAs may collaborate in breast cancer.

**Figure 7:**
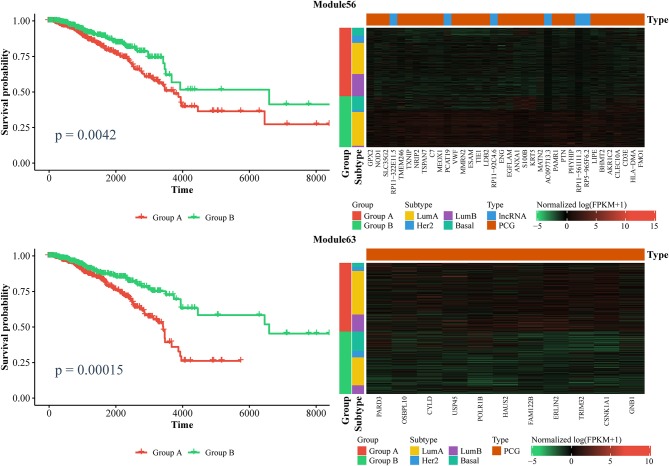
Kaplan–Meier curves of prognosis marker modules **(Left)** and the corresponding expression profiles **(Right)**.

Furthermore, we also identified ceRNA modules that can be considered prognostic markers for breast cancer. Similarly, we used the expression of ceRNAs in each module to classify patients into two groups via a k-means algorithm, and performed a Kaplan–Meier analysis. In total, we found six modules that can distinguish patients into two subgroups with significantly different survival times (log-rank test, *p* < 0.01). As shown in Figure 7 and Supplementary File 7, the patients with lower expressions in Module 63 and Module 270, as well as those with higher expression in Module 11, Module 25, Module 56, and Module 204 had longer survival time. Notably, Module 11 consists of lncRNAs. Collectively, these results confirm that ceRNAs play important roles in the treatment and prognosis of BRCA.

## Discussion

CeRNAs play critical roles in post-transcriptional regulation and are thus involved in many physiological and pathological progresses. An increasing number of non-coding RNAs that are able to influence their ceRNA partners via the ceRNA mechanism have been detected. Computational methods are an efficient approach for the detection and analysis of ceRNA relationships. By integrating the basic hypothesis and latest studies of ceRNA, we introduced dynamic correlation LA as one of the factors for detecting ceRNA pairs. Moreover, we integrated the multi-view NMF method with conventional PCC to detect ceRNA modules. Our results indicated that LA is effective in detecting ceRNA pairs and modules. The results of subsequent analysis showed that ceRNAs play important roles in breast cancer, especially in cell adhesion, cell migration, and cell-cell communication. Our results also demonstrated that ceRNAs may represent promising drug targets and markers for the treatment and prognosis of cancers.

In multi-view NMF, the parameters λ s balances the weight of different views. We set λ_*COR*_ = 1, and test λ_*LA*_ ∈ {1, 2, 3, 4, 5, 10, 15, 20, 25} in the BRCA dataset. The results showed that there is no significant difference among these parameters (Supplementary File 8), indicating that our method is not sensitive to these parameters.

The simplified calculation of LA demands that the distribution of the third variable follows standard normal distribution. We used the Van der Waerden's method to transform the microRNA expression profile to ensure it follows the standard normal distribution. We tested the performance of this method. We got a number of random data, which followed the negative binomial distribution. The sizes of the data set ranged from 10 to 2,000, with an increment of 20. We transform them by using Van der Waerden's method, respectively. Then we got another random data set following the standard normal distribution. whose size ranged from 1,000 to 100,000 with an increment of 1,000. We tested if the distribution of each pair between these two sets was similar by using Kolmogorov–Smirnov test. The result was shown in Supplementary File 10. The result suggested that the distribution of each pair has no significant difference.

LA is very convenient for the measurement of dynamic correlation. However, according to the results reported by Wei (Wei et al., 2019), the ceRNA strength forms a sharp peak around the most optimal microRNA concentration, indicating very large deviation of correlation. In this study, we used LA as a characteristic of ceRNA regulation. When the data contain the microRNA concentration ranging from the lower situation to higher situation, or symmetrically distributed on both sides of the most optimal microRNA concentration, the value of LA may be close to zero (according to the definition of LA). A solution to this problem is to measure the mean of the absolute value of the deviation, $LA^{\prime }\left(R1,R2|MIC\right)=E\left(\left|g^{\prime }\left(EXP_{MIC}\right)\right|\right)$, instead of $LA\left(R1,R2|MIC\right)=E\left(g^{\prime }\left(EXP_{MIC}\right)\right)$, where $g^{\prime }\left(EXP_{MIC}\right)$ is the deviation of correlation corresponding to *EXP*_*MIC*_. Moreover, LA is developed based on linear correlation PCC. However, real systems are more likely non-linear. Additional studies are warranted to determine the method for the calculation of the dynamic correlation of non-linear correlation.

Furthermore, besides the positive correlation and dynamic correlation, ceRNA pairs may have additional unknown features, which are also worthy of further investigation. The multi-view NMF framework has potential to integrate all these features for more accurate identification of ceRNA modules (Wang et al., 2018).

## Materials and Methods

### Datasets

We downloaded the RNA-seq data of mRNAs and lncRNAs, as well as the microRNA-seq data of patients with breast invasive carcinoma (BRCA) and liver hepatocellular carcinoma (LIHC) from TCGA (Weinstein et al., 2013). For patients with BRCA, we obtained the fragments per kilobase of exon model per million reads mapped (FPKM) values and read counts of mRNAs and lncRNAs from 1,090 tumor samples and 113 normal samples; we also obtained the read counts of mature microRNAs from 1,077 tumor samples and 104 normal samples. For patients with LIHC, we obtained the FPKM values and read counts of mRNAs and lncRNAs from 370 tumor samples and 50 normal samples; we also obtained the read counts of mature microRNAs from 371 tumor samples and 50 normal samples. Both microRNA-mRNA and microRNA-lncRNA interactions were downloaded from the mirwalk2.0 database (Dweep and Gretz, 2015), which incorporates predicted interactions curated in at least two of 13 different RNA-microRNA interaction databases, such as DIANA (Maragkakis et al., 2011) and miRDB (Wang and El Naqa, 2008). For further analysis, we downloaded the copy number variation data and DNA methylation data of patients with BRCA from TCGA.

### Data Preparation

Figure 1A shows the process of data preparation. Firstly, we only included samples that have complete mRNA, lncRNA, and microRNA expression data. We obtained 1,072 disease samples and 113 normal samples of BRCA, as well as 365 disease samples and 50 normal samples of LIHC. Subsequently, we excluded lowly expressed mRNAs, lncRNAs, and microRNAs. We only retained mRNAs with FPKM values >1 in >80% of disease or normal samples, lncRNAs with FPKM values >0.8 in >50% of disease or normal samples, and microRNAs with counts per million mapped reads values >100 in >50% of disease or normal samples (Mullokandov et al., 2012). Notably, we also excluded RNAs without interactions curated in the mirwalk2.0 database. Finally, we obtained 1,128 lncRNAs, 1,1172 mRNAs, and 137 mature microRNAs in BRCA, as well as 621 lncRNAs, 8,783 mRNAs, and 138 mature microRNAs in LIHC. All retained mRNAs and lncRNAs were considered candidate ceRNAs. We transformed the expression profiles of the candidate ceRNAs through log(*FPKM*+1) and those of mature microRNAs through log(*CPM*+1).

### Conventional Correlation Between RNAs

Current methods use conventional correlations as factors to predict ceRNA pairs. PCC and SI are most commonly used. We calculated the PCC value of a candidate ceRNA pair (e.g., *R*1, *R*2) as follows:

$$\begin{array}{l} \rho \left(R1,R2\right)=\frac{E\left[\left(EXP_{\text{R}1}-E\left(EXP_{\text{R}1}\right)\right)\times \left(EXP_{\text{R2}}-E\left(EXP_{\text{R2}}\right)\right)\right]}{\sqrt{Var\left(EXP_{\text{R}1}\right)\times Var\left(EXP_{\text{R2}}\right)}} \end{array}$$

where *EXP*_*R*1_ and *EXP*_*R*2_ represent the paired expression of *R*1 and *R*2, respectively. For the SI value of *R*1 and *R*2, we initially added the expression of shared microRNAs between *R*1 and *R*2. Subsequently, we calculated the partial correlation with respect to their shared microRNAs as follows:

$$\begin{array}{l} \rho \left(R1,R2|MIC\right)=\frac{\rho \left(R1,R2\right)-\rho \left(R1,MIC\right)\times \rho \left(R2,MIC\right)}{\sqrt{1-\rho^{2}\left(R1,MIC\right)}\times \sqrt{1-\rho^{2}\left(R2,MIC\right)}}, \end{array}$$

where *MIC* indicates the shared microRNAs between *R*1 and *R*2. Finally, we computed the SI value as follows:

$$\begin{array}{l} SI\left(R1,R2\right)=\rho \left(R1,R2\right)-\rho \left(R1,R2|MIC\right) \end{array}$$

### Statistical Significance of Shared MicroRNAs Between RNAs

CeRNAs are thought to usually share numerous microRNA targets. We used the hypergeometric test to calculate the significance of shared microRNAs for a given ceRNA pair (e.g., *R*1 and *R*2). The significance *p*-value can be obtained as follows:

$$\begin{array}{l} p\left(R1,R2\right)=1-\overset{q-1}{\underset{i=0}{\sum }}\frac{\left(\begin{array}{c} T\\ i \end{array}\right)\times \left(\begin{array}{c} Q-T\\ b-i \end{array}\right)}{\left(\begin{array}{c} Q\\ b \end{array}\right)} \end{array}$$

where *Q* is the total number of considered microRNAs, *T* is the number of microRNAs targeting candidate *R*1, *b* is the number of microRNAs targeting candidate *R*2, and *q* is the number of microRNAs targeting both candidates *R*1 and *R*2.

### Solution of the Objective Function

According to the multi-view NMF framework, we obtained the objective function as follows:

$$\begin{array}{l} \min({\left\|M^{PCC}-U^{PCC}{\left(V^{PCC}\right)}^{T}\right\|}_{F}^{2}+{\left\|M^{LA}-U^{LA}{\left(V^{LA}\right)}^{T}\right\|}_{F}^{2}\\ +\lambda_{PCC}{\left\|V^{PCC}-V^{*}\right\|}_{F}^{2}+\lambda_{LA}{\left\|V^{LA}-V^{*}\right\|}_{F}^{2})\\ \text{s}.\text{t}.\;\forall 1\leq k\leq K,{\left\|U_{\cdot ,k}^{PCC}\right\|}_{1}=1,{\left\|U_{\cdot ,k}^{LA}\right\|}_{1}\\ =1\text{ and }U^{PCC},U^{LA},V^{PCC},V^{LA},V^{*}\geq 0 \end{array}$$

where λ_*PCC*_, λ_*LA*_ tunes the relative weight among different views and between the standard NMF error and disagreement among (V^*^)*T*, (*V*^*PCC*^)*T* and (*V*^*LA*^)*T*. We used an iterative update procedure by updating one variable and maintaining the remaining variables fixed to solve this optimization problem. The specific iteration rules are listed as follows:

1. fixing V^*^, *V*^*PCC*^, and *V*^*LA*^, updating *U*^*PCC*^ and *U*^*LA*^, respectively:
  $$\begin{array}{l} U_{i,k}^{PCC}\leftarrow U_{i,k}^{PCC}\frac{{\left(M^{PCC}V^{PCC}\right)}_{i,k}+\lambda_{PCC}\overset{N}{\underset{j=1}{\sum }}V_{j,k}^{PCC}V_{j,k}^{\text{*}}}{{\left(U^{PCC}{\left(V^{PCC}\right)}^{T}V^{PCC}\right)}_{i,k}+\lambda_{PCC}\overset{M}{\underset{l=1}{\sum }}U_{l,k}^{PCC}\overset{N}{\underset{j=1}{\sum }}{\left(V_{j,k}^{PCC}\right)}^{2}}\\ U_{i,k}^{LA}\leftarrow U_{i,k}^{LA}\frac{{\left(M^{LA}V^{LA}\right)}_{i,k}+\lambda_{LA}\overset{N}{\underset{j=1}{\sum }}V_{j,k}^{LA}V_{j,k}^{\text{*}}}{{\left(U^{LA}{\left(V^{LA}\right)}^{T}V^{LA}\right)}_{i,k}+\lambda_{LA}\overset{M}{\underset{l=1}{\sum }}U_{l,k}^{LA}\overset{N}{\underset{j=1}{\sum }}{\left(V_{j,k}^{LA}\right)}^{2}} \end{array}$$
2. fixing V^*^, *U*^*PCC*^, and *U*^*LA*^, updating *V*^*PCC*^ and *V*^*LA*^, respectively:
  $$\begin{array}{l} V_{j,k}^{PCC}\leftarrow V_{j,k}^{PCC}\frac{{\left({\left(M^{PCC}\right)}^{T}U^{PCC}\right)}_{j,k}+\lambda_{PCC}V_{j,k}^{*}}{{\left(V^{PCC}{\left(U^{PCC}\right)}^{T}U^{PCC}\right)}_{j,k}+\lambda_{PCC}V_{j,k}^{PCC}}\\ V_{j,k}^{LA}\leftarrow V_{j,k}^{LA}\frac{{\left({\left(M^{LA}\right)}^{T}U^{LA}\right)}_{j,k}+\lambda_{LA}V_{j,k}^{*}}{{\left(V^{LA}{\left(U^{LA}\right)}^{T}U^{LA}\right)}_{j,k}+\lambda_{LA}V_{j,k}^{v}} \end{array}$$
3. fixing *V*^*PCC*^, *V*^*LA*^, *U*^*PCC*^, and *U*^*LA*^, updating V^*^:
  $$\begin{array}{l} V^{*}=\frac{\lambda_{PCC}V^{PCC}Q^{PCC}+\lambda_{LA}V^{LA}Q^{LA}}{\lambda_{PCC}+\lambda_{LA}}, \end{array}$$
  where
  $$\begin{array}{l} Q^{PCC}=Diag\left(\overset{G}{\underset{i=1}{\sum }}U_{i,1}^{PCC},\overset{G}{\underset{i=1}{\sum }}U_{i,2}^{PCC},\cdots \;,\overset{G}{\underset{i=1}{\sum }}U_{i,K}^{PCC}\right)\\ Q^{LA}=Diag\left(\overset{G}{\underset{i=1}{\sum }}U_{i,1}^{LA},\overset{G}{\underset{i=1}{\sum }}U_{i,2}^{LA},\cdots \;,\overset{G}{\underset{i=1}{\sum }}U_{i,K}^{LA}\right) \end{array}$$

More details can be found in Liu et al. (2013).

### Disease-Associated Genes Filter

We retained the patients with both tumor and normal samples and used them to identify differentially expressed genes using the R package edgeR software (Robinson et al., 2010). We set genes with *FDR* < 0.05, |log *FC*| > 1 as differentially expressed genes.

Apart from the differentially expressed genes, we investigated the BRCA-associated copy number variation (CNV) genes and differentially methylated genes to further investigate the BRCA associated modules. This was based on the notion that the expression of these genes may be altered and reflect the ceRNA interactions. We calculated the level of copy number variation for each gene in each sample using GISTIC2.0 software (Mermel et al., 2011), and identified genes that did not equal two in >5% of samples as CNV genes.

For DNA methylation, we used Illumina 450K methylation data. We initially identified differentially methylated sites (DMS) using Limma (Ritchie et al., 2015). The sites, which showed statistical significance with a *FDR* < 0.05 and had mean methylation differences between disease and normal states >0.2, were marked as DMS. Subsequently, we isolated the sites located in the regions between 2,000bp upstream and downstream of the start position of ceRNA candidate genes and mapped them to related genes. For genes related to more than one DMS and more than three-quarters of these DMSs exhibit the same direction of change in methylation, we set these genes as differential methylation genes (Kim et al., 2012).

## Data Availability Statement

The RNA-seq data, microRNA-seq data, CNV data and gene methylation data are from The Cancer Genome Atlas (TCGA). The source codes of LAceModule is available at https://github.com/GaoLabXDU/LAceModule.

## Author Contributions

XW and LG conceived the study. XW and YH designed the experiments. XW performed the experiments and analyses. All authors discussed the results and contributed to the final manuscript.

### Conflict of Interest

The authors declare that the research was conducted in the absence of any commercial or financial relationships that could be construed as a potential conflict of interest.

## Supplementary Material

Supplementary Table 1 Most frequently enriched KEGG pathways.

Supplementary Table 2 Summary of BRCA-dysregulated genes.

Supplementary Table 3 Top 15 most frequently shared microRNAs in more than three modules.

Supplementary File 1 Heatmap of PCC and LA values of modules enriched for GO:0007155 in disease and normal states (top right: disease state, bottom left: normal state).

Supplementary File 2 Heatmap of PCC and LA values of modules enriched for KEGG:04514 in disease and normal states (top right: disease state, bottom left: normal state).

Supplementary File 3 Heatmap of PCC and LA values of modules enriched for KEGG:04510 in disease and normal states (top right: disease state, bottom left: normal state).

Supplementary File 4 Profiles of differentially expressed genes.

Supplementary File 5 BRCA-associated modules (Supplementary of Figure 5A).

Supplementary File 6 Kaplan–Meier curves for individual genes in Figure 6C.

Supplementary File 7 Kaplan–Meier curves of prognosis marker modules (left) and the corresponding expression profiles (right). (Supplementary of Figure 7).

Supplementary File 8 Comparison of the results of different values for the parameter λ_*LA*_.

Supplementary File 9 Patients groups by the expression of let-7 family and the gene set in Figure 6.

Supplementary File 10 The evaluation of Van der Waerden's method.
