# Supplementary material for: LAceModule: Identification of Competing Endogenous RNA Modules by Integrating Dynamic Correlation
Source: Front Genet. 2020 Mar 18;11:235. doi: 10.3389/fgene.2020.00235 (PMC7093494; doi:10.3389/fgene.2020.00235)
Supplement: Supplementary File 1 — Heatmap of PCC and LA values of modules enriched for GO:0007155 in disease and normal states (top right: disease state, bottom left: normal state). [file Data_Sheet_1.PDF]

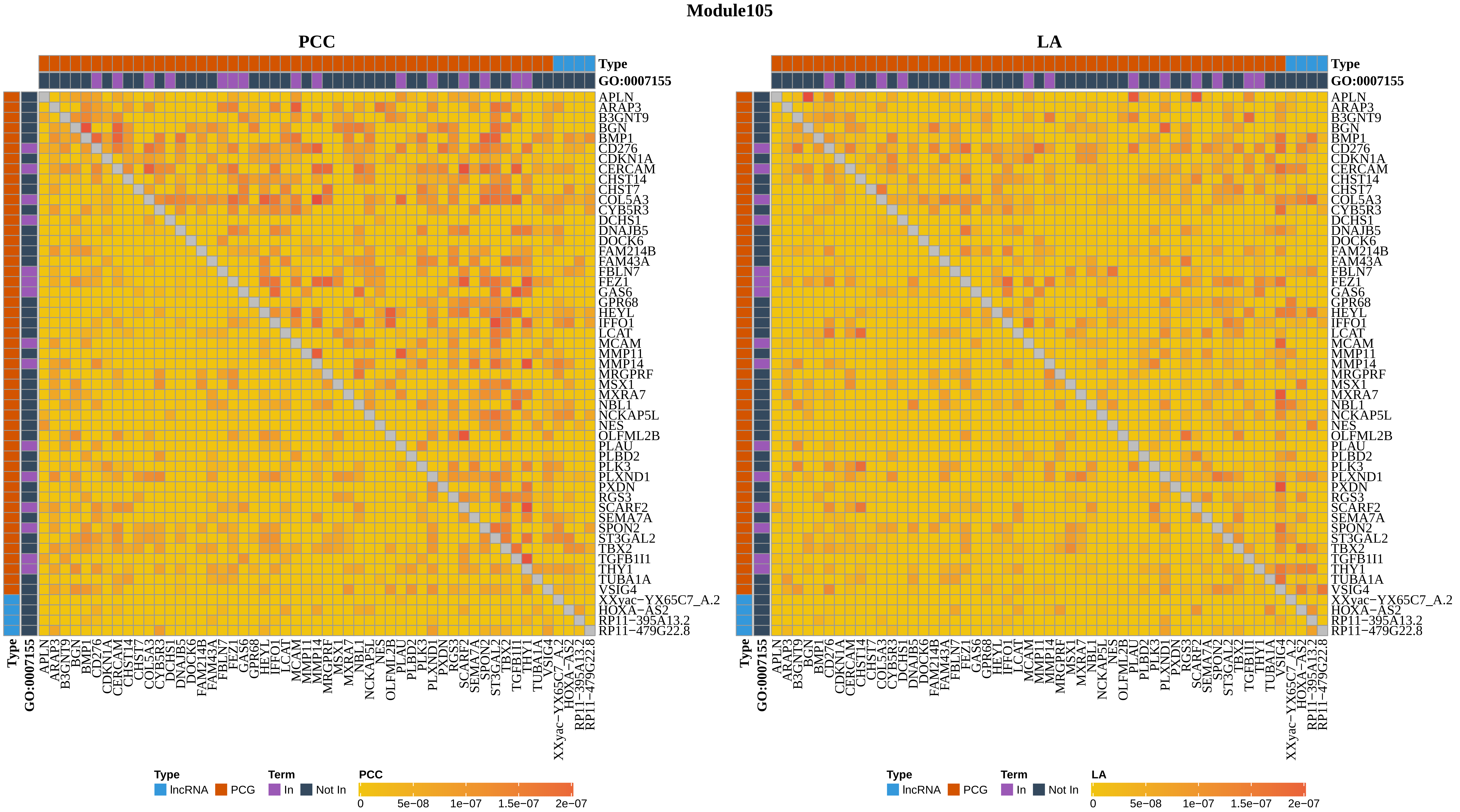
Module105
PCC
LA
Type
GO:0007155
APLN
ARAP3
B3GNT9
BGN
BMP1
CD276
CDKN1A
CERCAM
CHST14
CHST7
COL5A3
CYB5R3
DCHS1
DNAJB5
DOCK6
FAM214B
FAM43A
FBLN7
FEZ1
GAS6
GPR68
HEYL
IFFO1
LCAT
MCAM
MMP11
MMP14
MRGPRF
MSX1
MXRA7
NBL1
NCKAP5L
NES
OLFML2B
PLAU
PLBD2
PLK3
PLXND1
PXDN
RGS3
SCARF2
SEMA7A
SPON2
ST3GAL2
TBX2
TGFB1I1
THY1
TUBA1A
VSIG4
XXyac−YX65C7_A.2
HOXA−AS2
RP11−395A13.2
RP11−479G22.8
Type
lncRNA
PCG
Term
In
Not In
PCC
0
5e−08
1e−07
1.5e−07
2e−07
LA
0
5e−08
1e−07
1.5e−07
2e−07

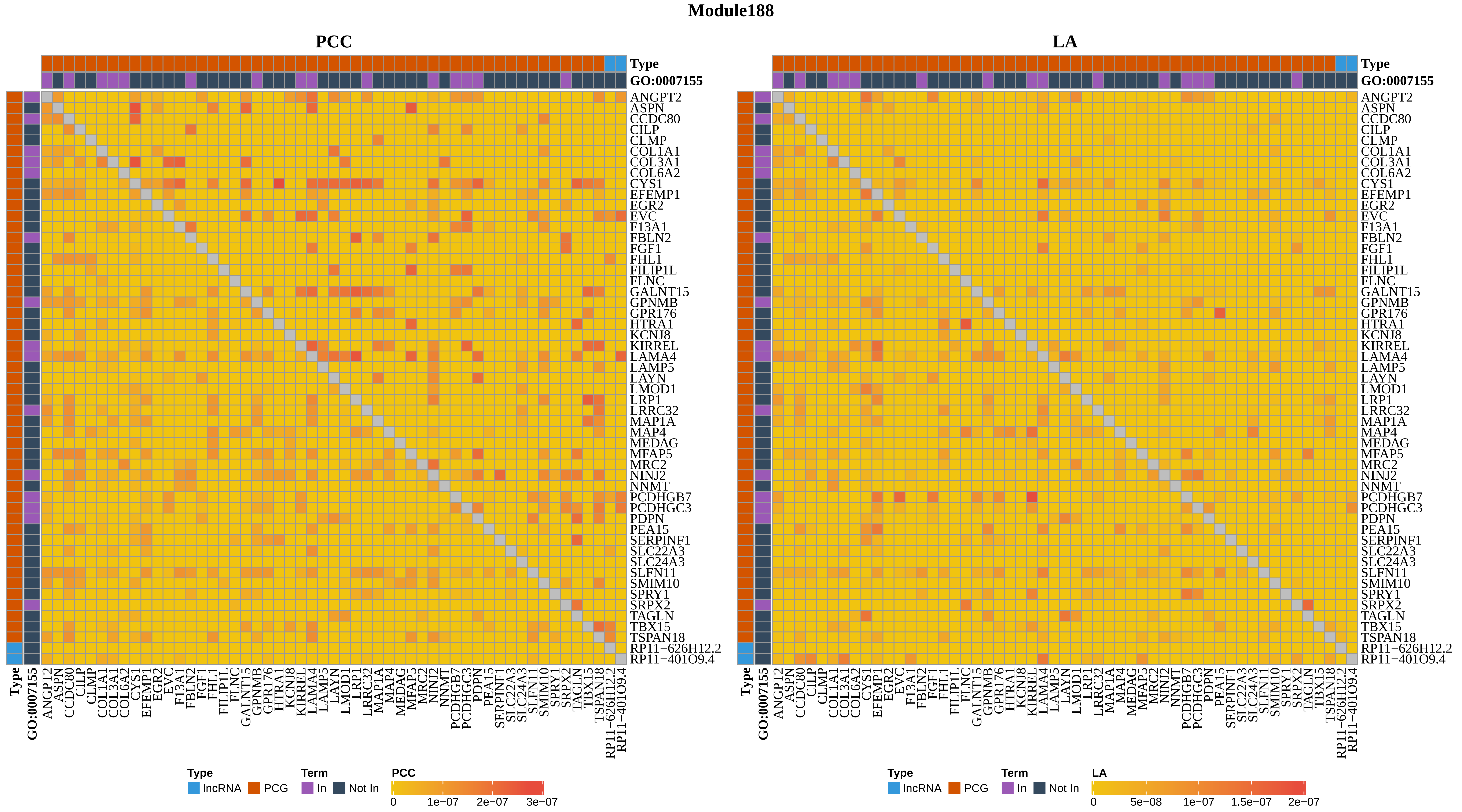
Module188
PCC
LA
Type
GO:0007155
ANGPT2
ASPN
CCDC80
CILP
CLMP
COL1A1
COL3A1
COL6A2
CYS1
EFEMP1
EGR2
EVC
F13A1
FBLN2
FGF1
FHL1
FILIP1L
FLNC
GALNT15
GPNMB
GPR176
HTRA1
KCNJ8
KIRREL
LAMA4
LAMP5
LAYN
LMOD1
LRP1
LRRC32
MAP1A
MAP4
MEDAG
MFAP5
MRC2
NINJ2
NNMT
PCDHGB7
PCDHGC3
PDPN
PEA15
SERPINF1
SLC22A3
SLC24A3
SLFN11
SMIM10
SPRY1
SRPX2
TAGLN
TBX15
TSPAN18
RP11−626H12.2
RP11−401O9.4
Type
lncRNA
PCG
Term
In
Not In
PCC
0
1e−07
2e−07
3e−07
LA
0
5e−08
1e−07
1.5e−07
2e−07

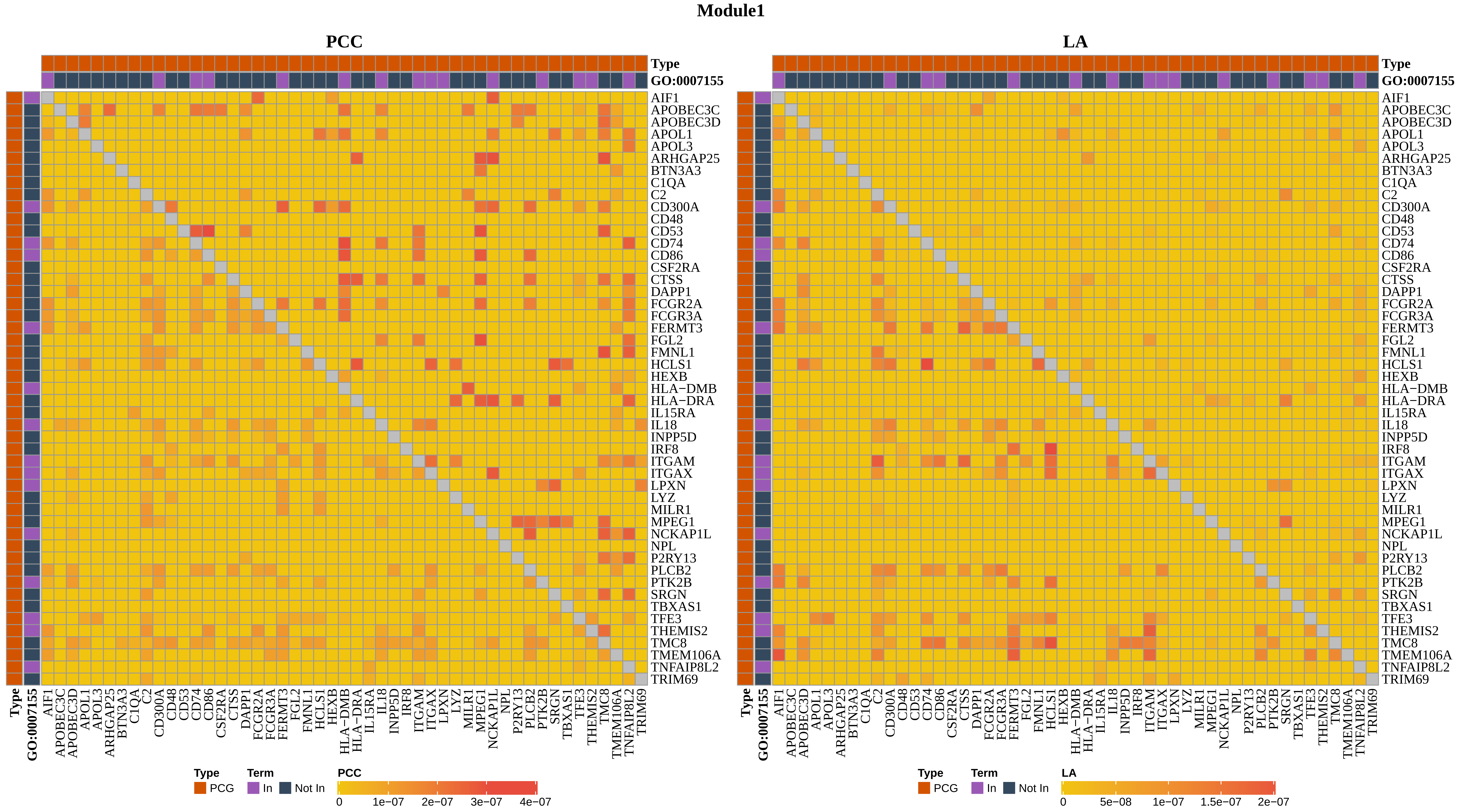
Module1
PCC
LA
Type
GO:0007155
AIF1
APOBEC3C
APOBEC3D
APOL1
APOL3
ARHGAP25
BTN3A3
C1QA
C2
CD300A
CD48
CD53
CD74
CD86
CSF2RA
CTSS
DAPP1
FCGR2A
FCGR3A
FERMT3
FGL2
FMNL1
HCLS1
HEXB
HLA−DMB
HLA−DRA
IL15RA
IL18
INPP5D
IRF8
ITGAM
ITGAX
LPXN
LYZ
MILR1
MPEG1
NCKAP1L
NPL
P2RY13
PLCB2
PTK2B
SRGN
TBXAS1
TFE3
THEMIS2
TMC8
TMEM106A
TNFAIP8L2
TRIM69
Type: PCG
Term: In, Not In
PCC: 0, 1e−07, 2e−07, 3e−07, 4e−07
LA: 0, 5e−08, 1e−07, 1.5e−07, 2e−07

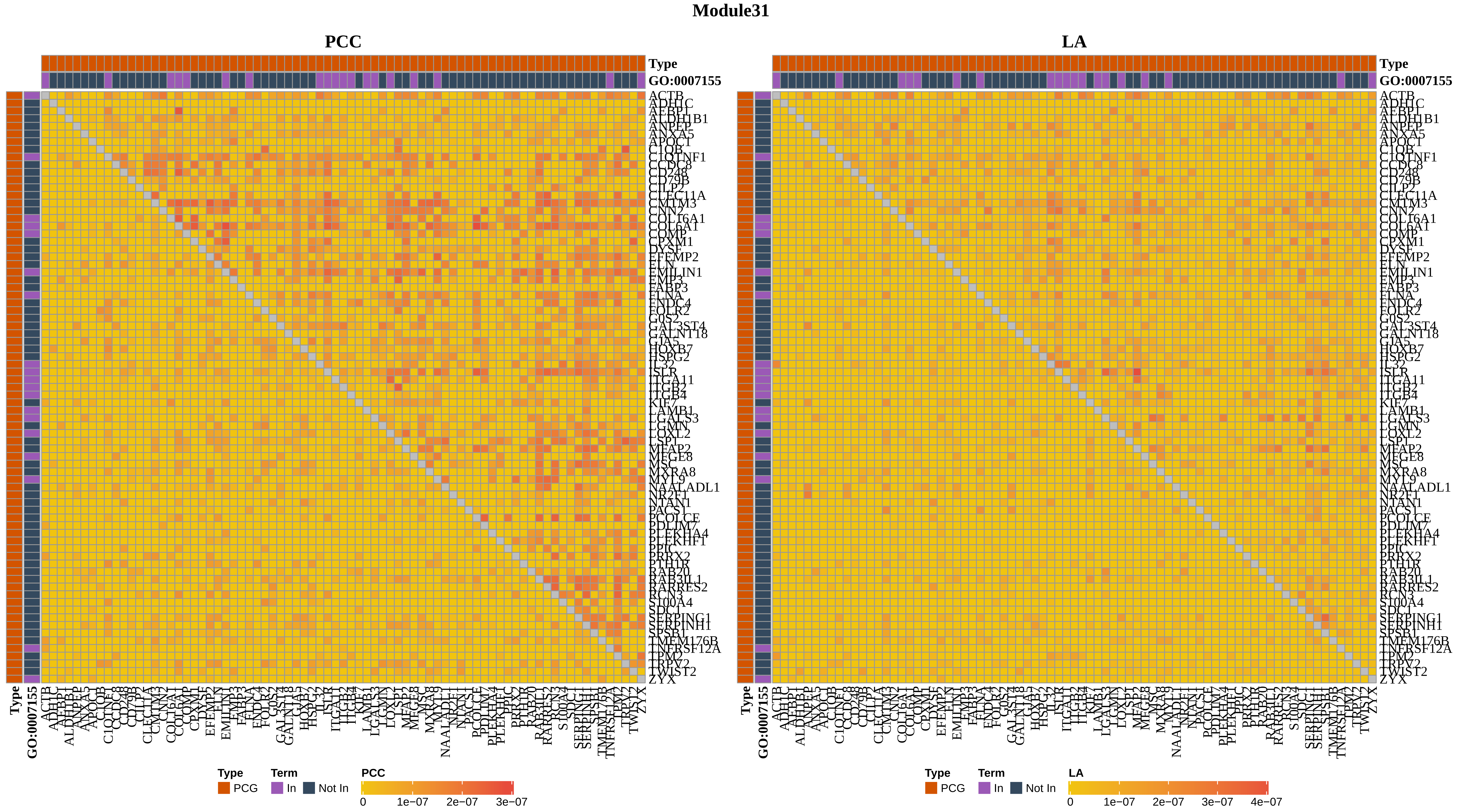
Module31
PCC
LA
Type
GO:0007155
ACTB
ADH1C
AEBP1
ALDH1B1
ANPEP
ANXA5
APOC1
C1QB
C1QTNF1
CCDC8
CD248
CD79B
CILP2
CLEC11A
CMTM3
CNN2
COL16A1
COL6A1
COMP
CPXM1
DYSF
EFEMP2
ELN
EMILIN1
EMP3
FABP3
FLNA
FNDC4
FOLR2
G0S2
GAL3ST4
GALNT18
GJA5
HOXB7
HSPG2
IL32
ISLR
ITGA11
ITGB2
ITGB4
KIF7
LAMB1
LGALS3
LGMN
LOXL2
LSP1
MFAP2
MFGE8
MSC
MXRA8
MYL9
NAALADL1
NR2F1
NTAN1
PACS1
PCOLCE
PDLIM7
PLEKHA4
PLEKHF1
PPIC
PRRX2
PTH1R
RAB20
RAB3IL1
RARRES2
RCN3
S100A4
SDC1
SERPING1
SERPINH1
SPSB1
TMEM176B
TNFRSF12A
TPM2
TRPV2
TWIST2
ZYX
Type
PCG
Term
In
Not In
PCC
0
1e−07
2e−07
3e−07
LA
0
1e−07
2e−07
3e−07
4e−07

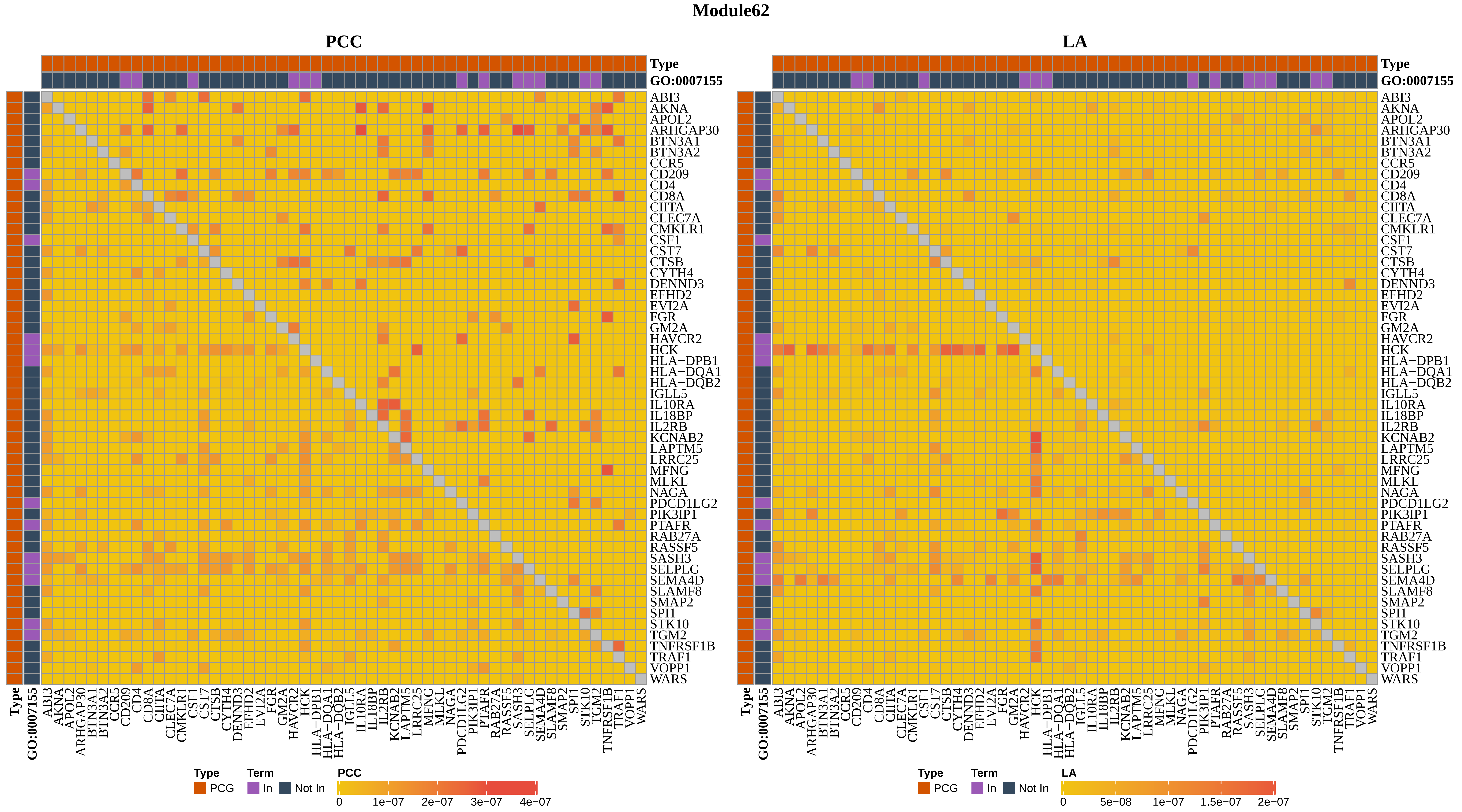
Module62
PCC
LA
Type
GO:0007155
ABI3 AKNA APOL2 ARHGAP30 BTN3A1 BTN3A2 CCR5 CD209 CD4 CD8A CIITA CLEC7A CMKLR1 CSF1 CST7 CTSB CYTH4 DENND3 EFHD2 EVI2A FGR GM2A HAVCR2 HCK HLA−DPB1 HLA−DQA1 HLA−DQB2 IGLL5 IL10RA IL18BP IL2RB KCNAB2 LAPTM5 LRRC25 MFNG MLKL NAGA PDCD1LG2 PIK3IP1 PTAFR RAB27A RASSF5 SASH3 SELPLG SEMA4D SLAMF8 SMAP2 SPI1 STK10 TGM2 TNFRSF1B TRAF1 VOPP1 WARS
Type: PCG
Term: In, Not In
PCC: 0, 1e−07, 2e−07, 3e−07, 4e−07
LA: 0, 5e−08, 1e−07, 1.5e−07, 2e−07

Module261

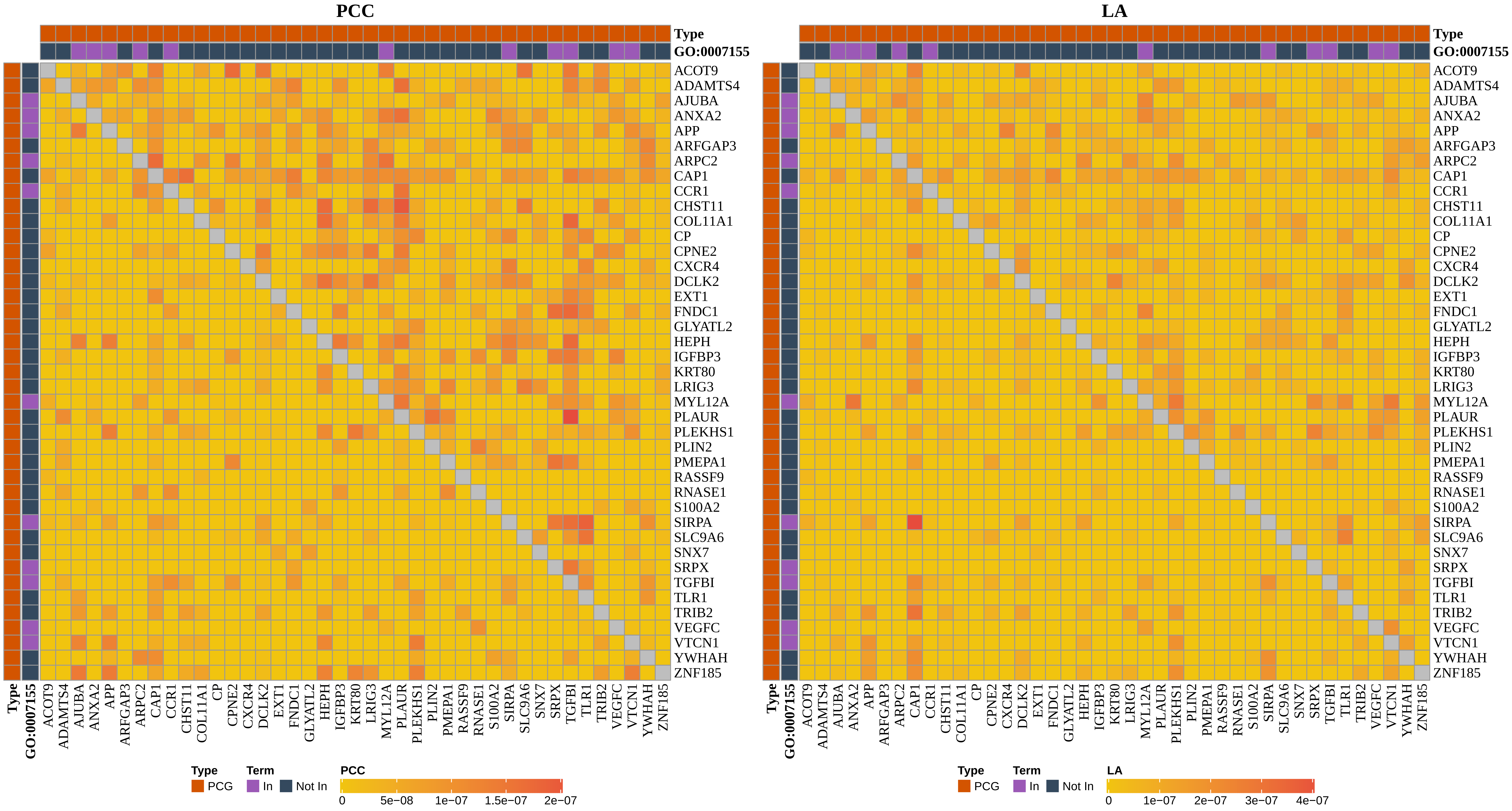
PCC
LA
Type
GO:0007155
ACOT9
ADAMTS4
AJUBA
ANXA2
APP
ARFGAP3
ARPC2
CAP1
CCR1
CHST11
COL11A1
CP
CPNE2
CXCR4
DCLK2
EXT1
FNDC1
GLYATL2
HEPH
IGFBP3
KRT80
LRIG3
MYL12A
PLAUR
PLEKHS1
PLIN2
PMEPA1
RASSF9
RNASE1
S100A2
SIRPA
SLC9A6
SNX7
SRPX
TGFBI
TLR1
TRIB2
VEGFC
VTCN1
YWHAH
ZNF185
Type
PCG
Term
In
Not In
PCC
0
5e−08
1e−07
1.5e−07
2e−07
LA
0
1e−07
2e−07
3e−07
4e−07

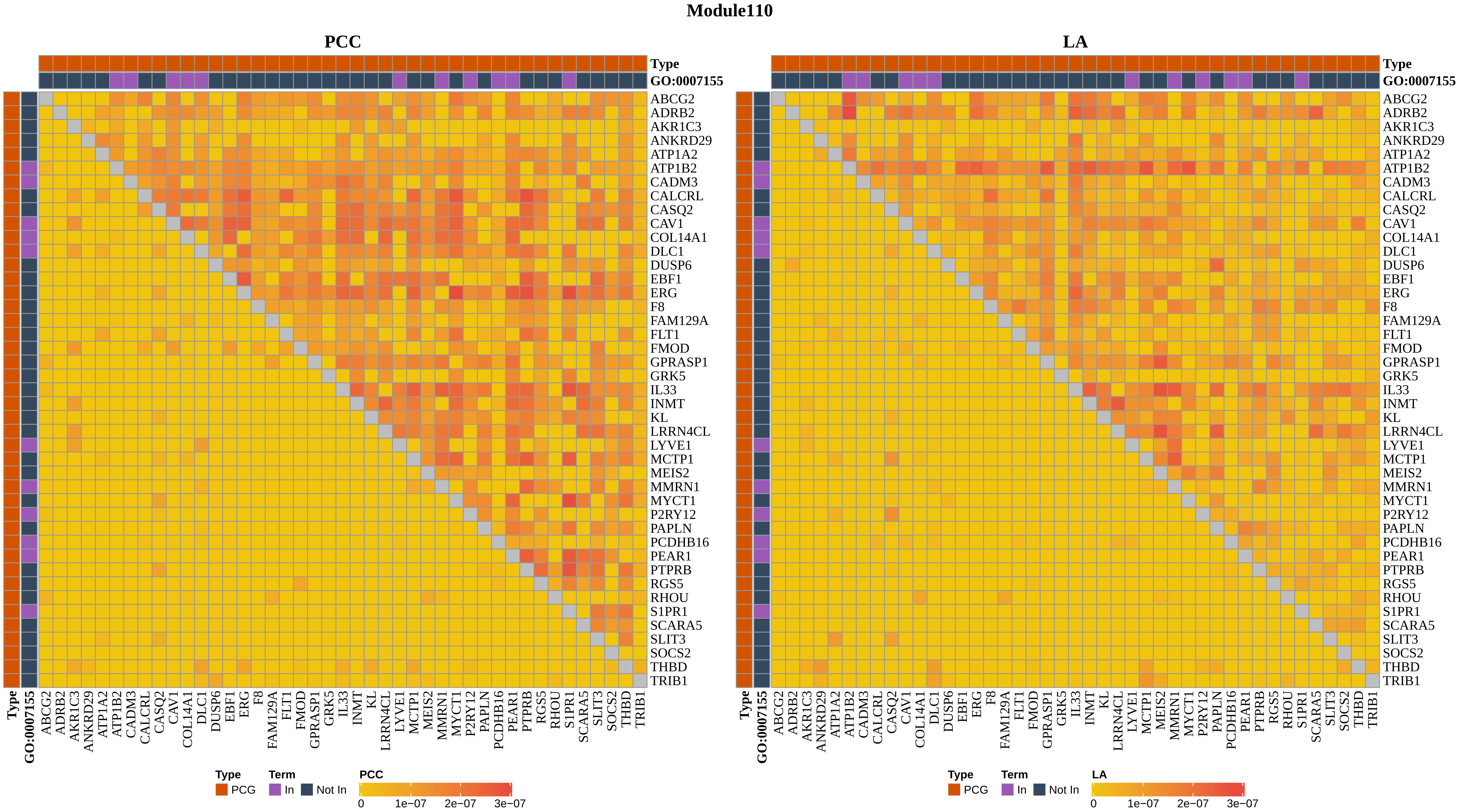
Module110
PCC
LA
Type
GO:0007155
ABCG2
ADRB2
AKR1C3
ANKRD29
ATP1A2
ATP1B2
CADM3
CALCRL
CASQ2
CAV1
COL14A1
DLC1
DUSP6
EBF1
ERG
F8
FAM129A
FLT1
FMOD
GPRASP1
GRK5
IL33
INMT
KL
LRRN4CL
LYVE1
MCTP1
MEIS2
MMRN1
MYCT1
P2RY12
PAPLN
PCDHB16
PEAR1
PTPRB
RGS5
RHOU
S1PR1
SCARA5
SLIT3
SOCS2
THBD
TRIB1
Type
PCG
Term
In
Not In
PCC
0
1e−07
2e−07
3e−07
LA
0
1e−07
2e−07
3e−07

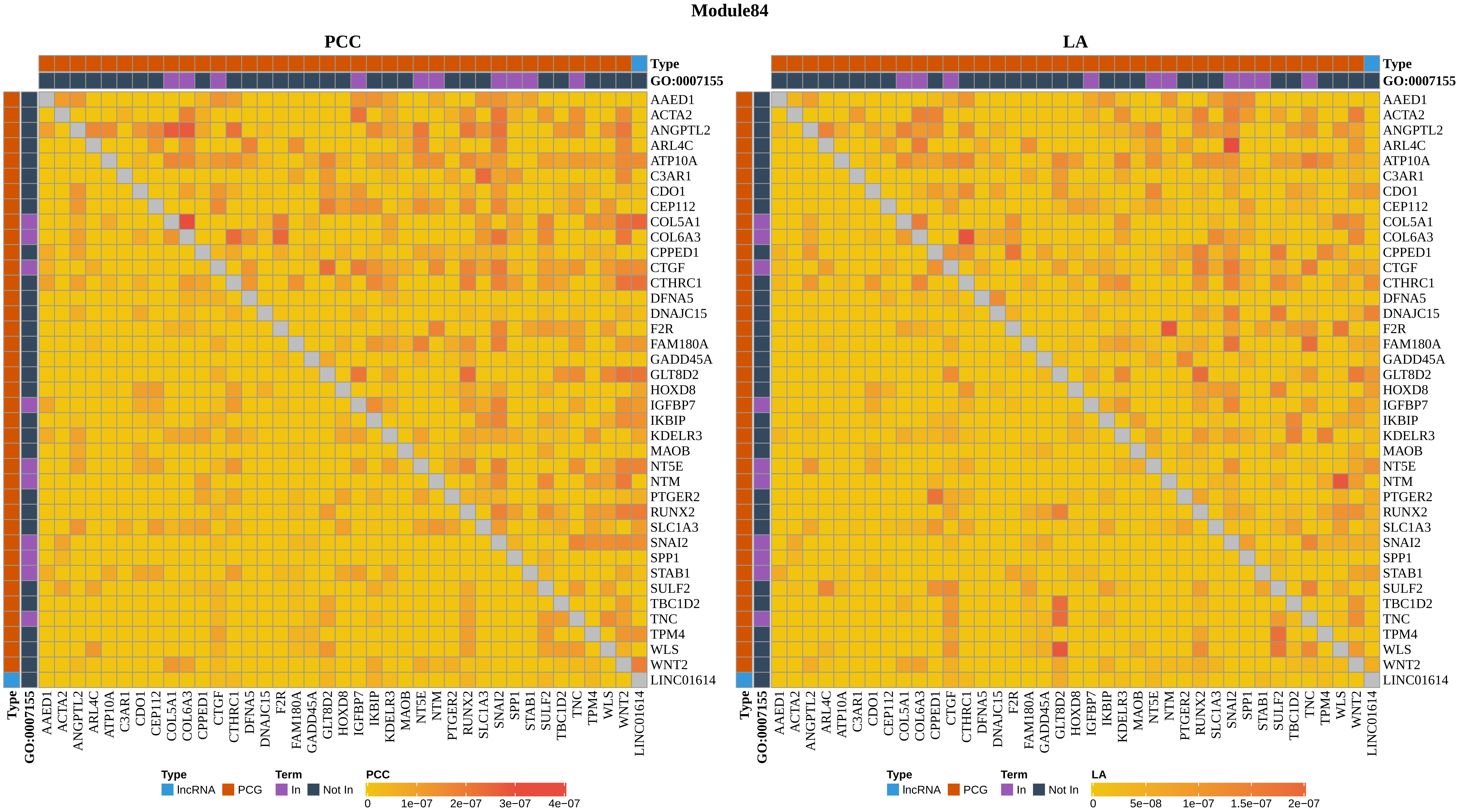
Module84
PCC
LA
Type
GO:0007155
AAED1
ACTA2
ANGPTL2
ARL4C
ATP10A
C3AR1
CDO1
CEP112
COL5A1
COL6A3
CPPED1
CTGF
CTHRC1
DFNA5
DNAJC15
F2R
FAM180A
GADD45A
GLT8D2
HOXD8
IGFBP7
IKBIP
KDELR3
MAOB
NT5E
NTM
PTGER2
RUNX2
SLC1A3
SNAI2
SPP1
STAB1
SULF2
TBC1D2
TNC
TPM4
WLS
WNT2
LINC01614
Type
lncRNA
PCG
Term
In
Not In
PCC
0
1e−07
2e−07
3e−07
4e−07
LA
0
5e−08
1e−07
1.5e−07
2e−07

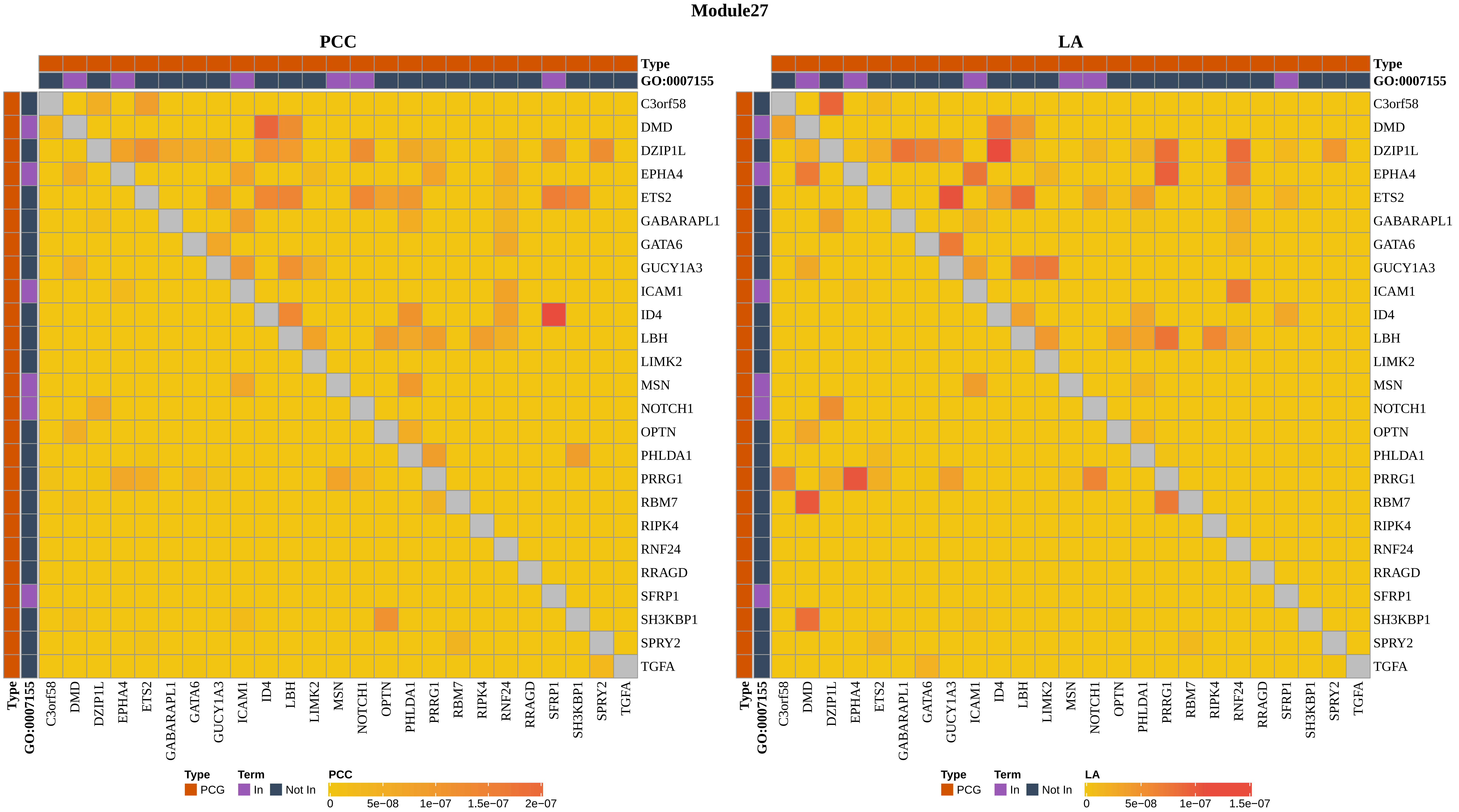

Module27
PCC
LA
Type
GO:0007155
C3orf58
DMD
DZIP1L
EPHA4
ETS2
GABARAPL1
GATA6
GUCY1A3
ICAM1
ID4
LBH
LIMK2
MSN
NOTCH1
OPTN
PHLDA1
PRRG1
RBM7
RIPK4
RNF24
RRAGD
SFRP1
SH3KBP1
SPRY2
TGFA
Type
PCG
Term
In
Not In
PCC
0
5e−08
1e−07
1.5e−07
2e−07
LA
0
5e−08
1e−07
1.5e−07

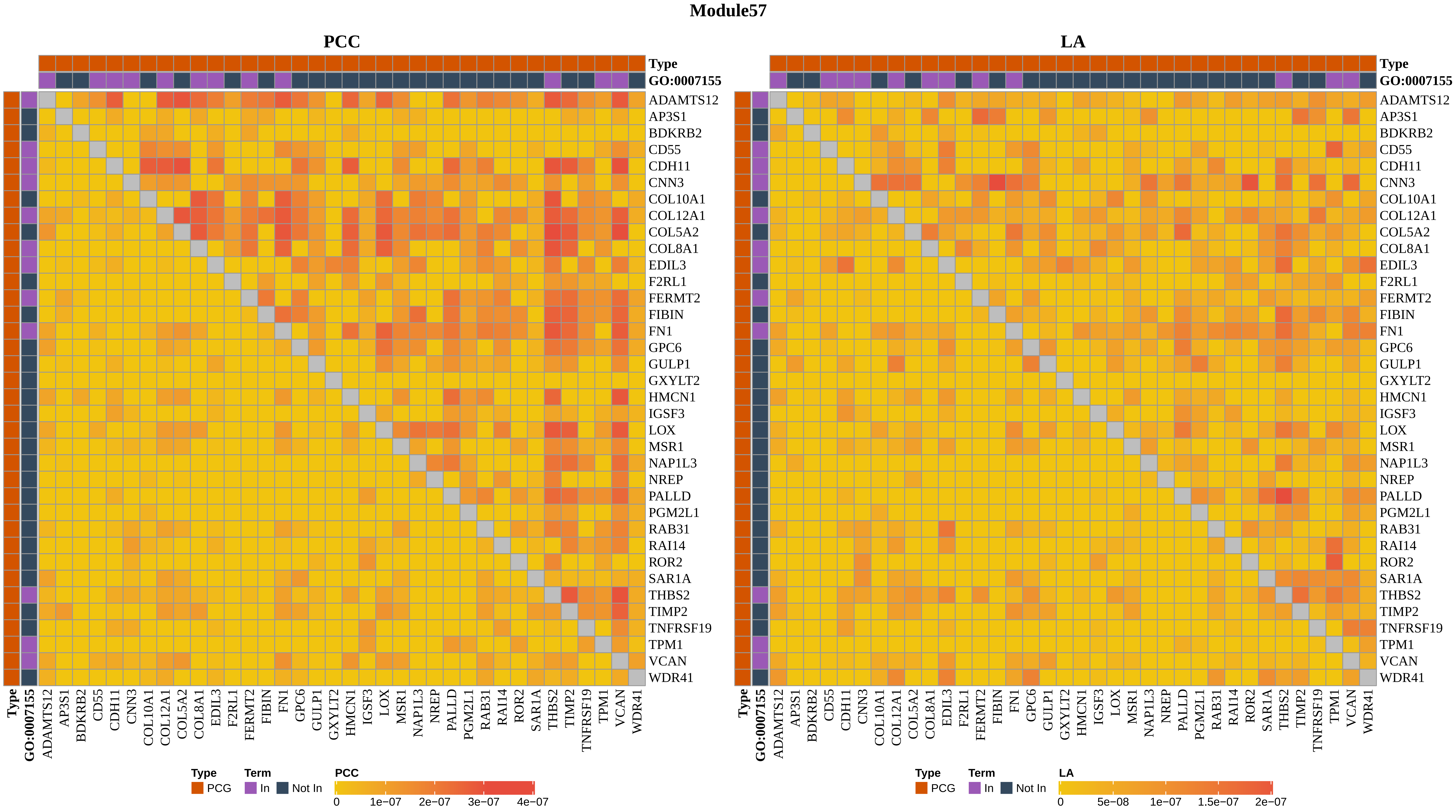
Module57
PCC
Type
GO:0007155
ADAMTS12
AP3S1
BDKRB2
CD55
CDH11
CNN3
COL10A1
COL12A1
COL5A2
COL8A1
EDIL3
F2RL1
FERMT2
FIBIN
FN1
GPC6
GULP1
GXYLT2
HMCN1
IGSF3
LOX
MSR1
NAP1L3
NREP
PALLD
PGM2L1
RAB31
RAI14
ROR2
SAR1A
THBS2
TIMP2
TNFRSF19
TPM1
VCAN
WDR41
Type
PCG
Term
In
Not In
PCC
0
1e−07
2e−07
3e−07
4e−07
LA
Type
GO:0007155
ADAMTS12
AP3S1
BDKRB2
CD55
CDH11
CNN3
COL10A1
COL12A1
COL5A2
COL8A1
EDIL3
F2RL1
FERMT2
FIBIN
FN1
GPC6
GULP1
GXYLT2
HMCN1
IGSF3
LOX
MSR1
NAP1L3
NREP
PALLD
PGM2L1
RAB31
RAI14
ROR2
SAR1A
THBS2
TIMP2
TNFRSF19
TPM1
VCAN
WDR41
Type
PCG
Term
In
Not In
LA
0
5e−08
1e−07
1.5e−07
2e−07

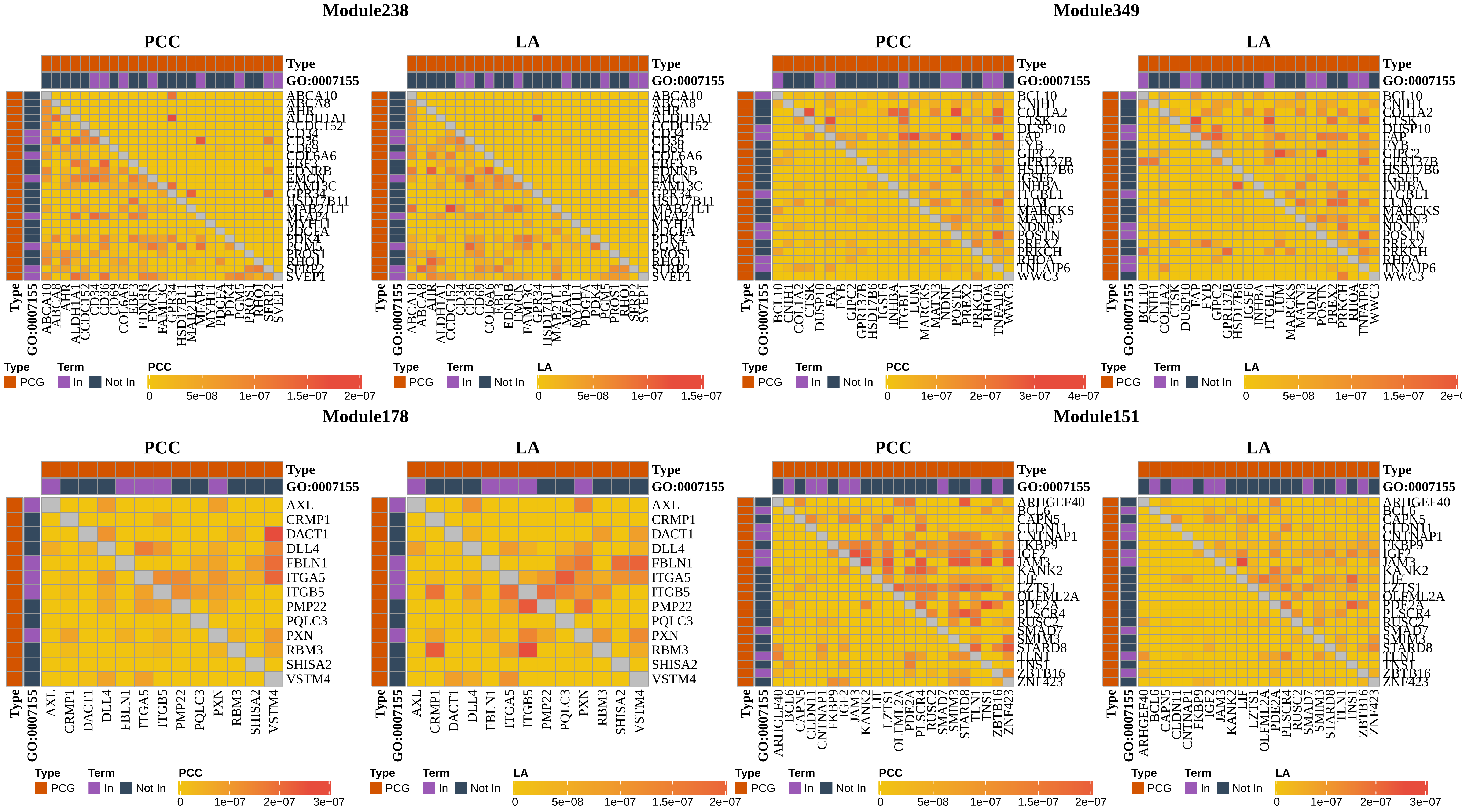

Module238
PCC
LA
Type
GO:0007155
ABCA10
ABCA8
AHR
ALDH1A1
CCDC152
CD34
CD36
CD69
COL6A6
EBF3
EDNRB
EMCN
FAM13C
GPR34
HSD17B11
MAB21L1
MFAP4
MYH11
PDGFA
PDK4
PGM5
PROS1
RHOJ
SFRP2
SVEP1
Type PCG
Term In Not In
PCC 0 5e−08 1e−07 1.5e−07 2e−07
LA 0 5e−08 1e−07 1.5e−07
Module349
PCC
LA
Type
GO:0007155
BCL10
CNIH1
COL1A2
CTSK
DUSP10
FAP
FYB
GIPC2
GPR137B
HSD17B6
IGSF6
INHBA
ITGBL1
LUM
MARCKS
MATN3
NDNF
POSTN
PREX2
PRKCH
RHOA
TNFAIP6
WWC3
Type PCG
Term In Not In
PCC 0 1e−07 2e−07 3e−07 4e−07
LA 0 5e−08 1e−07 1.5e−07 2e−
Module178
PCC
LA
Type
GO:0007155
AXL
CRMP1
DACT1
DLL4
FBLN1
ITGA5
ITGB5
PMP22
PQLC3
PXN
RBM3
SHISA2
VSTM4
Type PCG
Term In Not In
PCC 0 1e−07 2e−07 3e−07
LA 0 5e−08 1e−07 1.5e−07 2e−07
Module151
PCC
LA
Type
GO:0007155
ARHGEF40
BCL6
CAPN5
CLDN11
CNTNAP1
FKBP9
IGF2
JAM3
KANK2
LIF
LZTS1
OLFML2A
PDE2A
PLSCR4
RUSC2
SMAD7
SMIM3
STARD8
TLN1
TNS1
ZBTB16
ZNF423
Type PCG
Term In Not In
PCC 0 5e−08 1e−07 1.5e−07 2e−07
LA 0 1e−07 2e−07 3e−07
