# Supplementary figures and images for: LAceModule: Identification of Competing Endogenous RNA Modules by Integrating Dynamic Correlation
Source: Front Genet. 2020 Mar 18;11:235. doi: 10.3389/fgene.2020.00235 (PMC7093494; doi:10.3389/fgene.2020.00235)

Module246

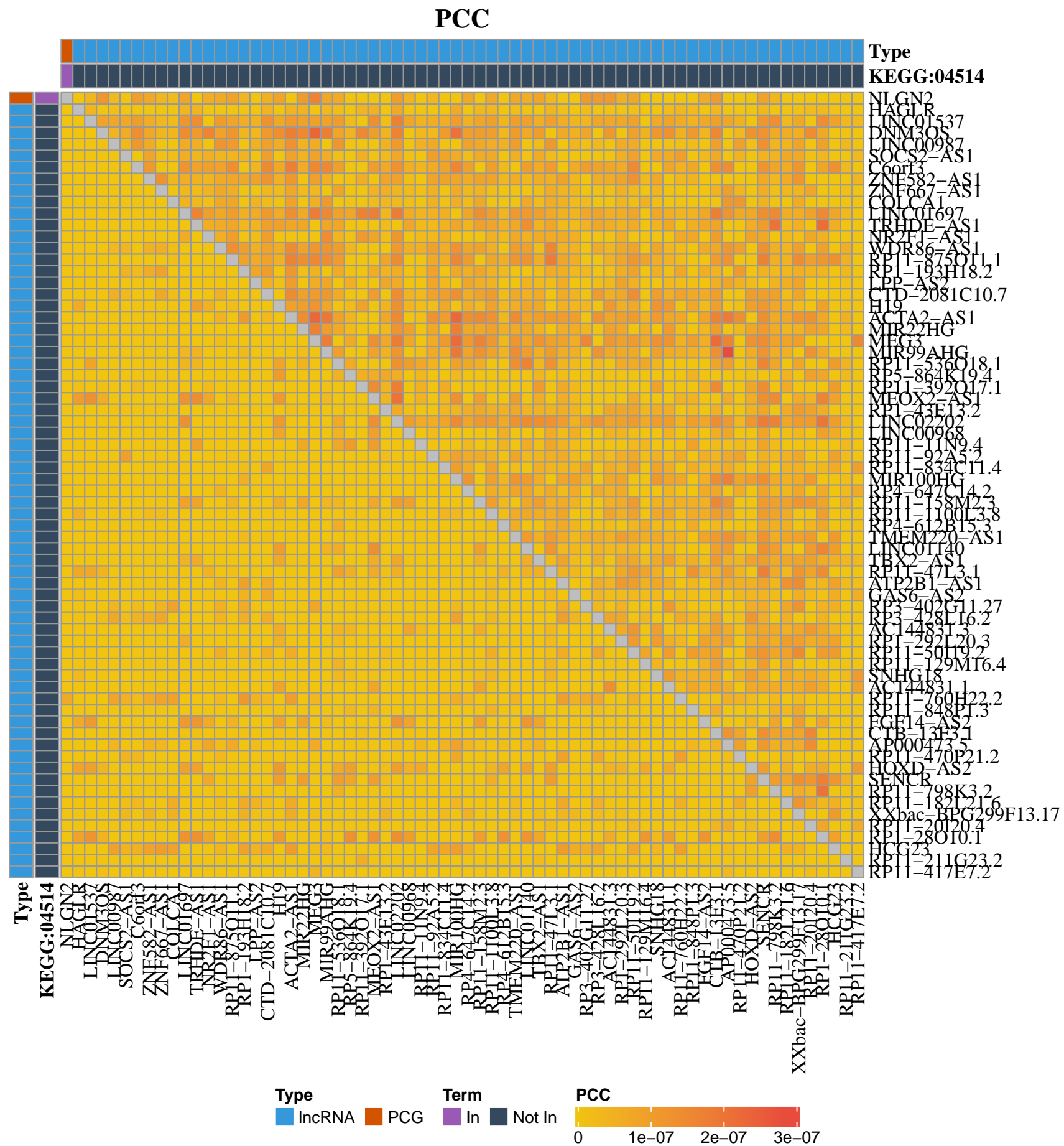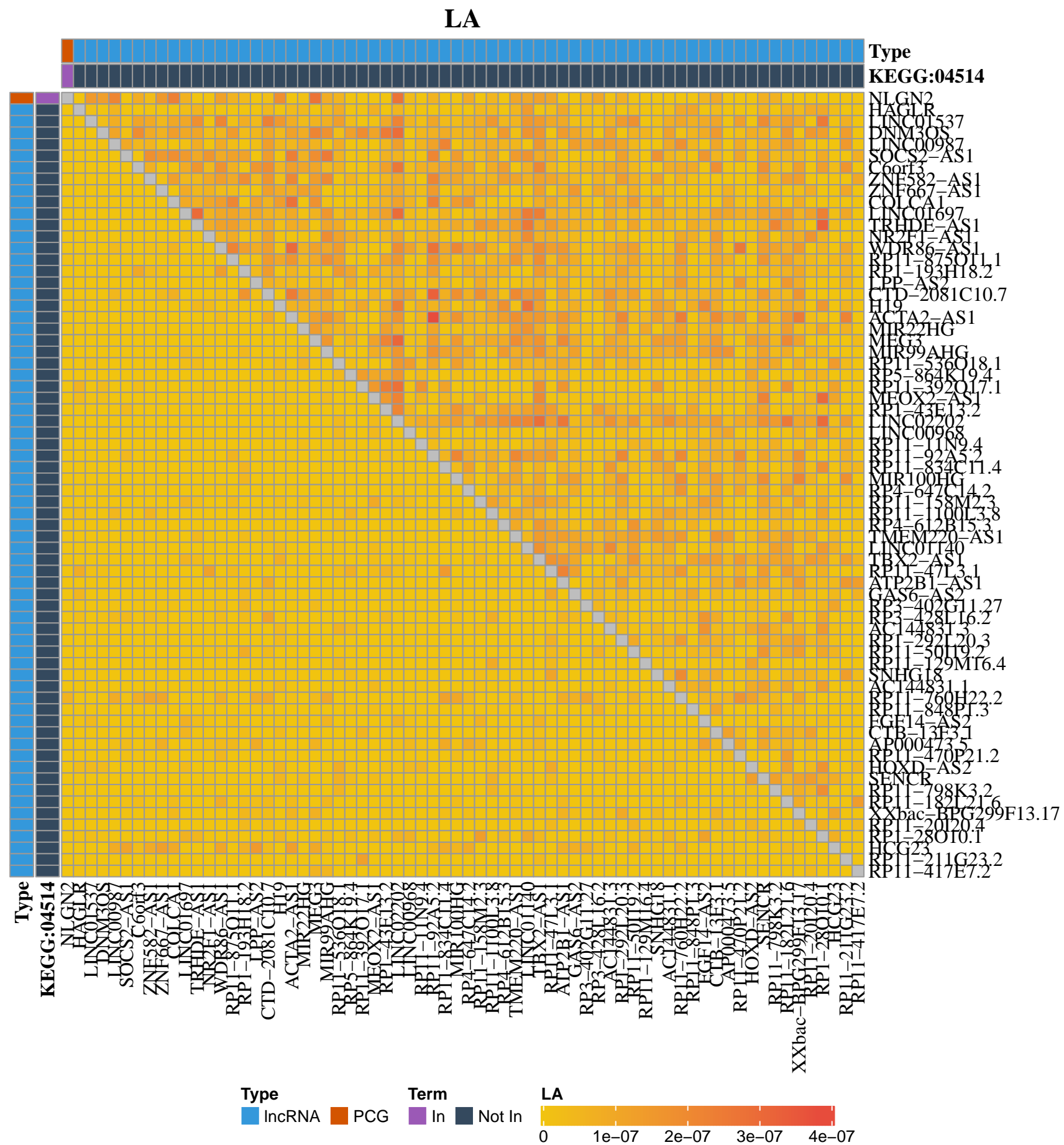

Module1

PCC

LA

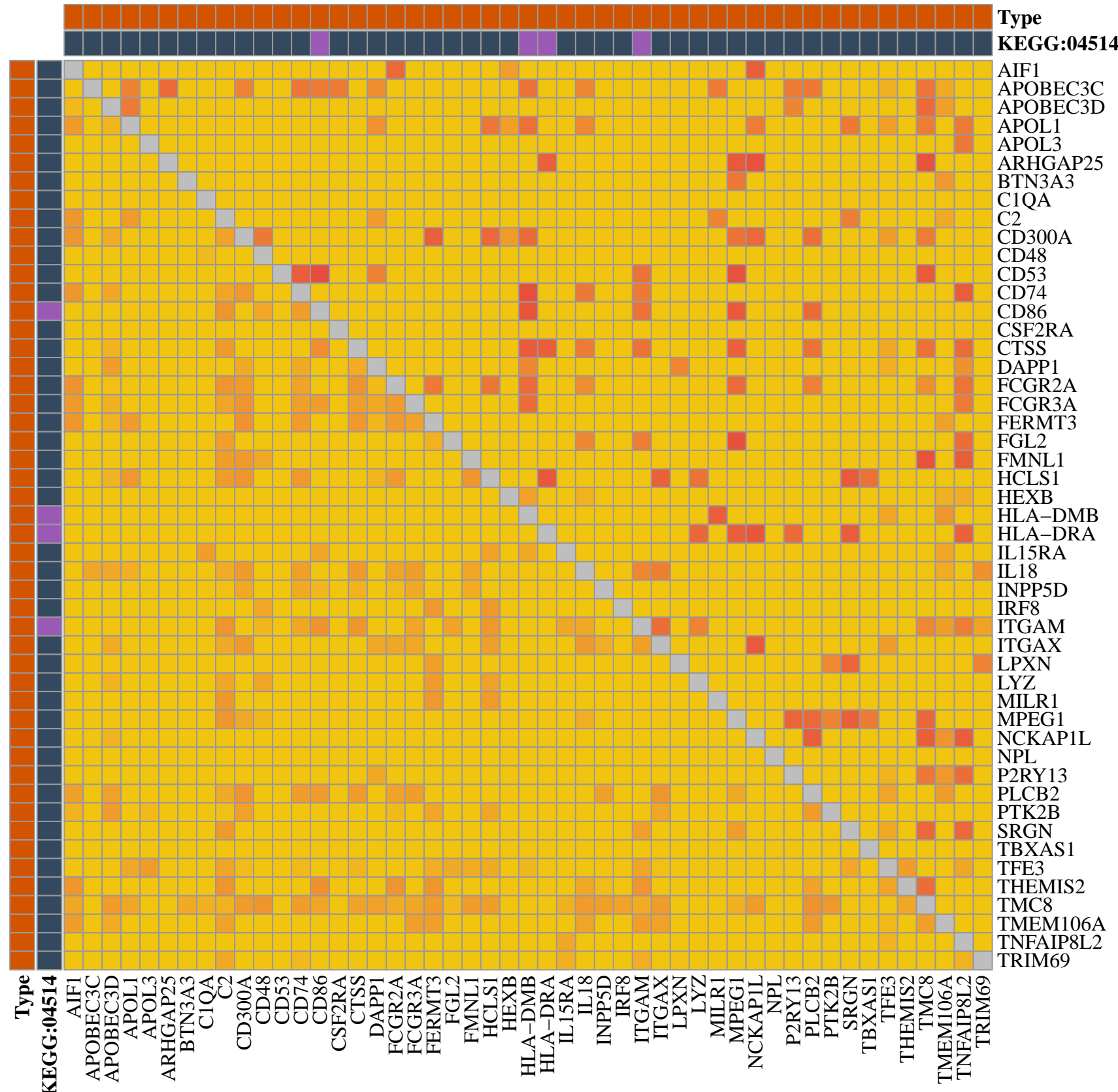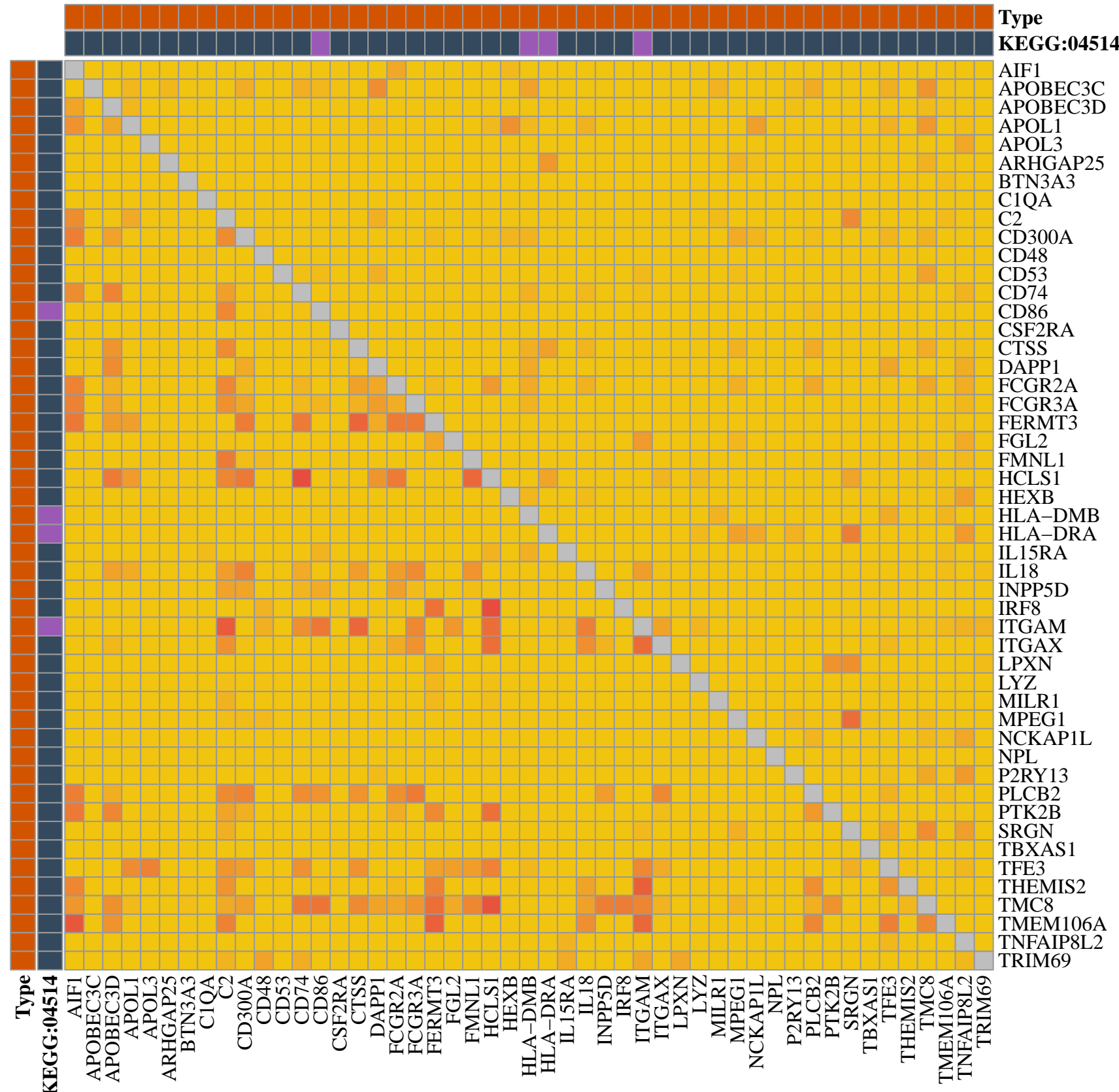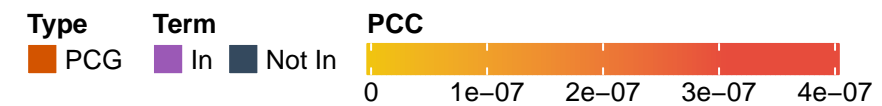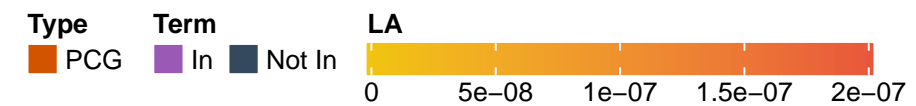

Module72

PCC

LA

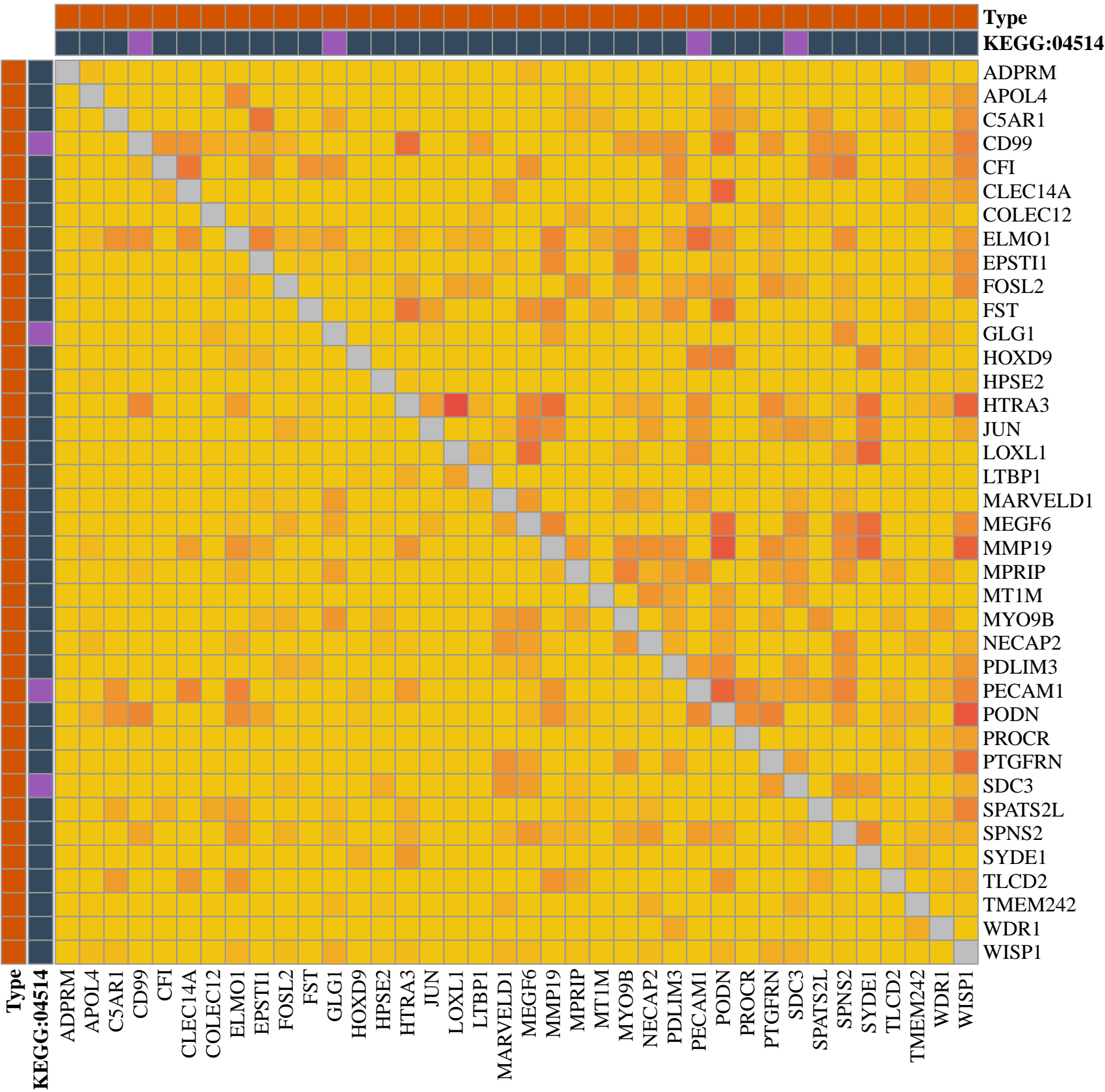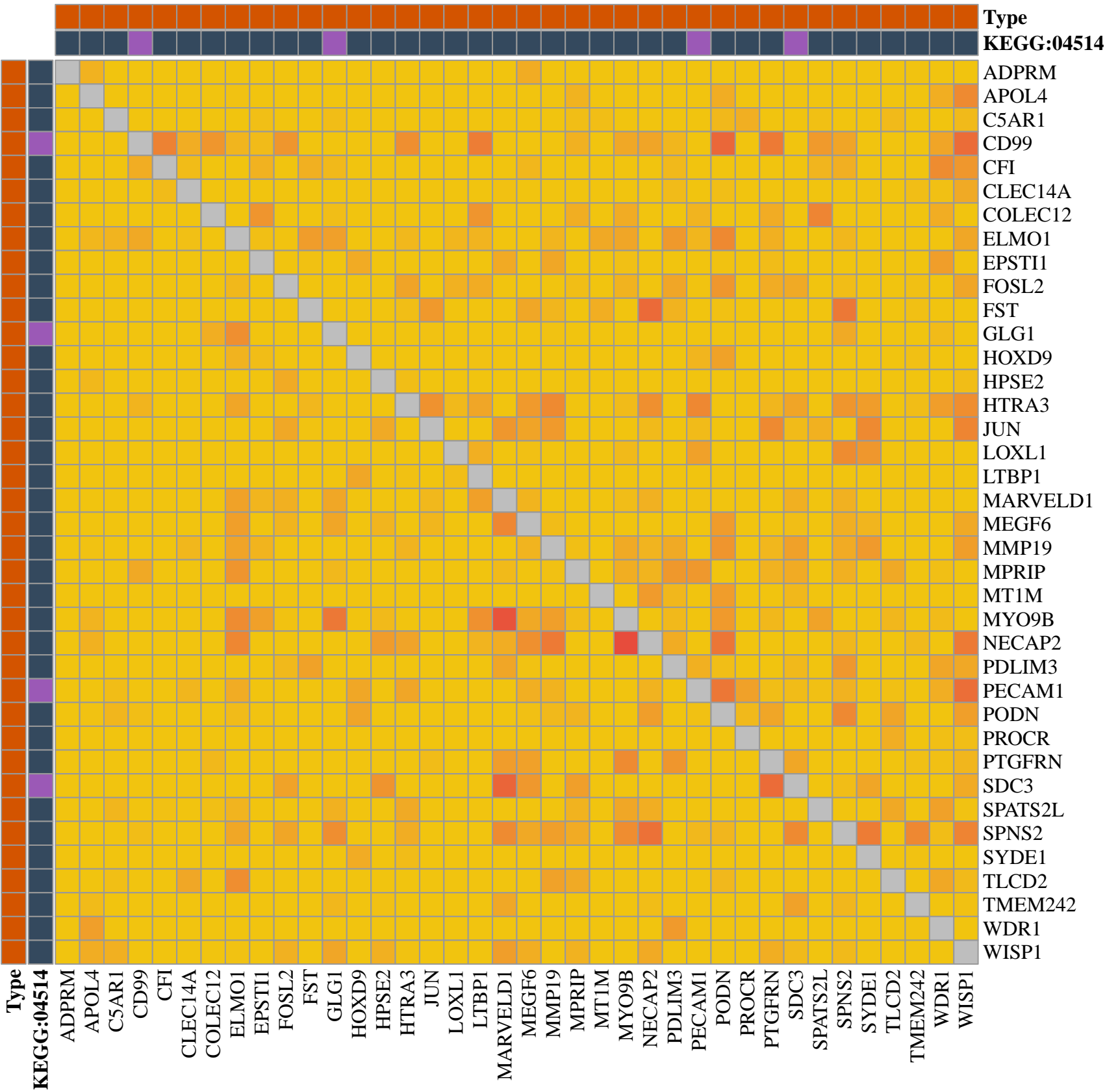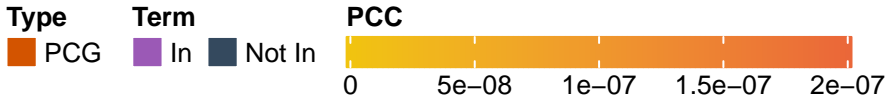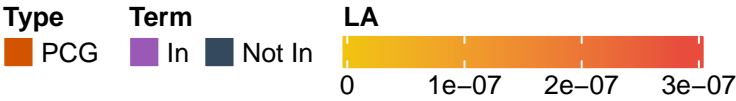



Module62

PCC

LA

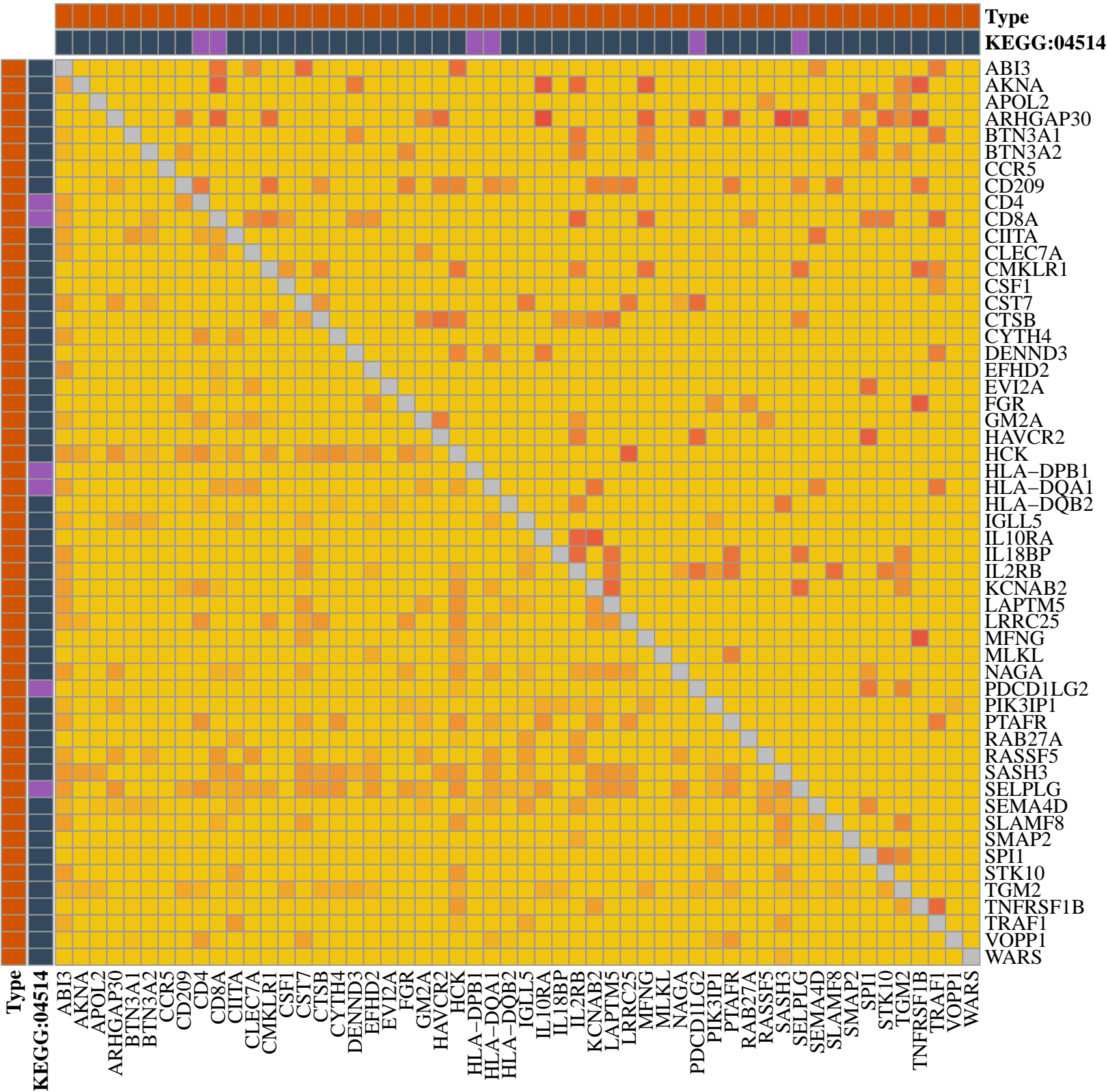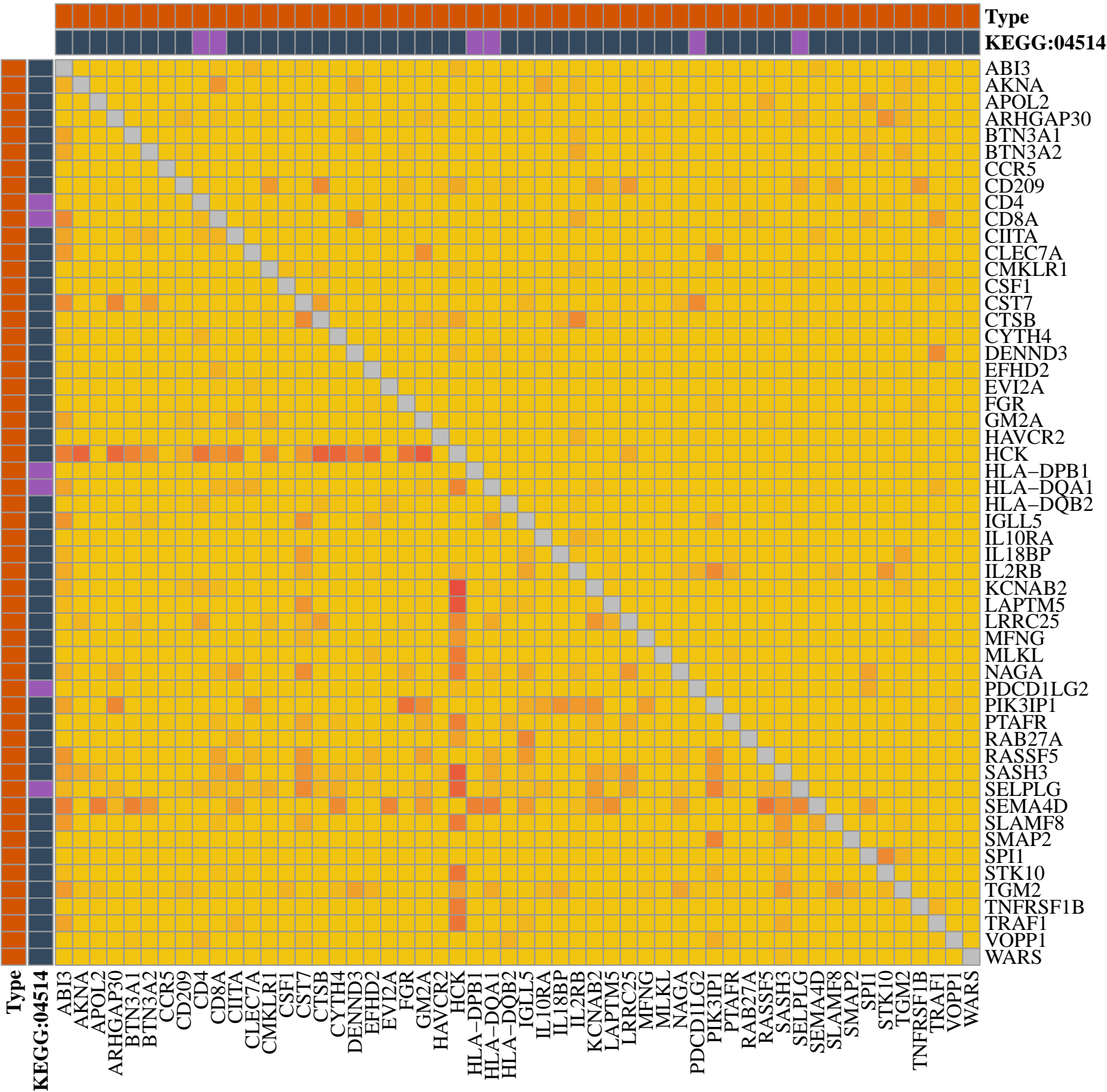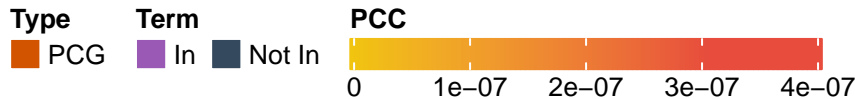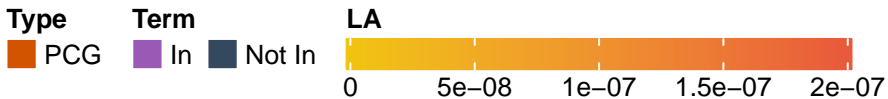

Module333

PCC

LA

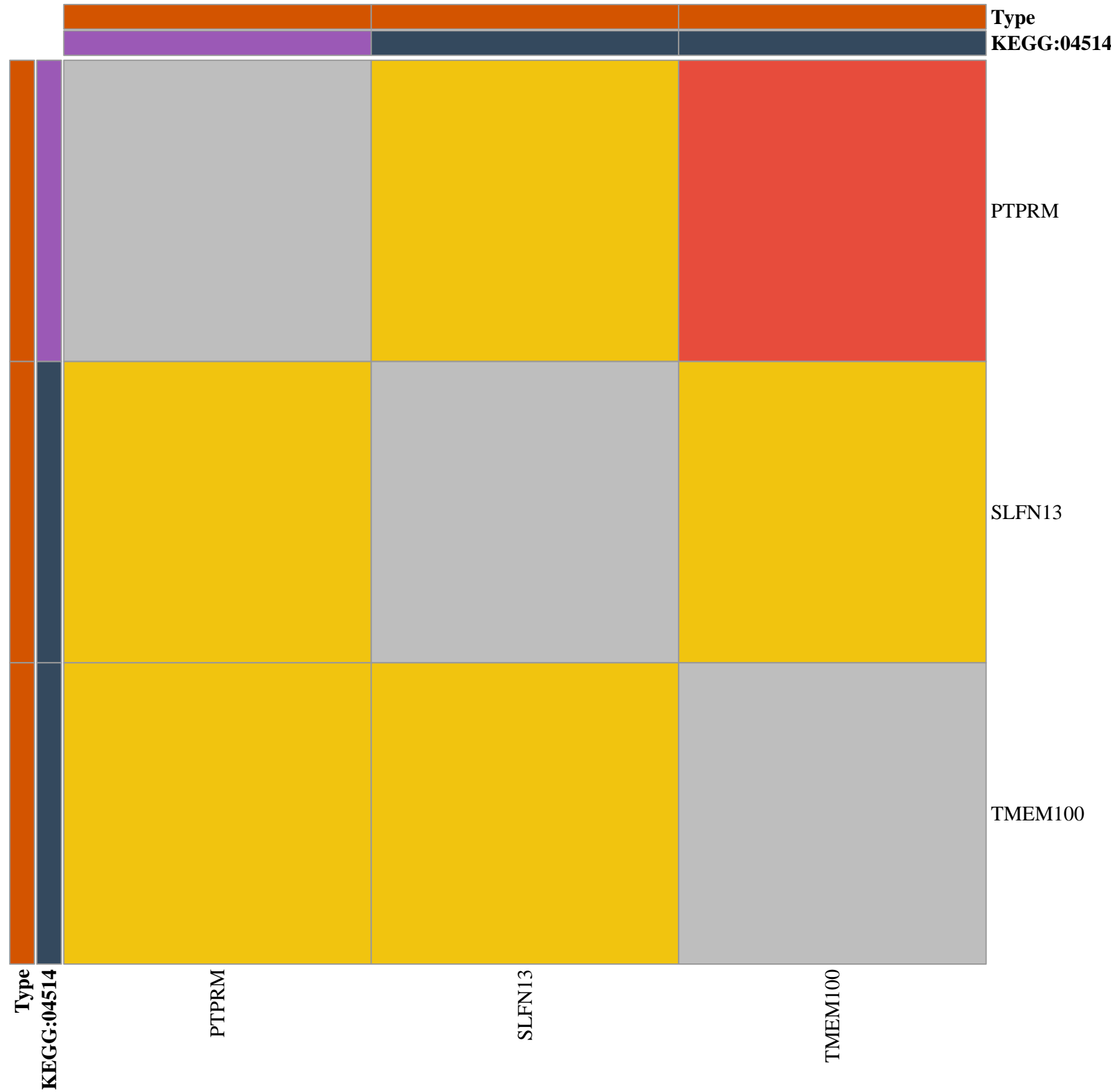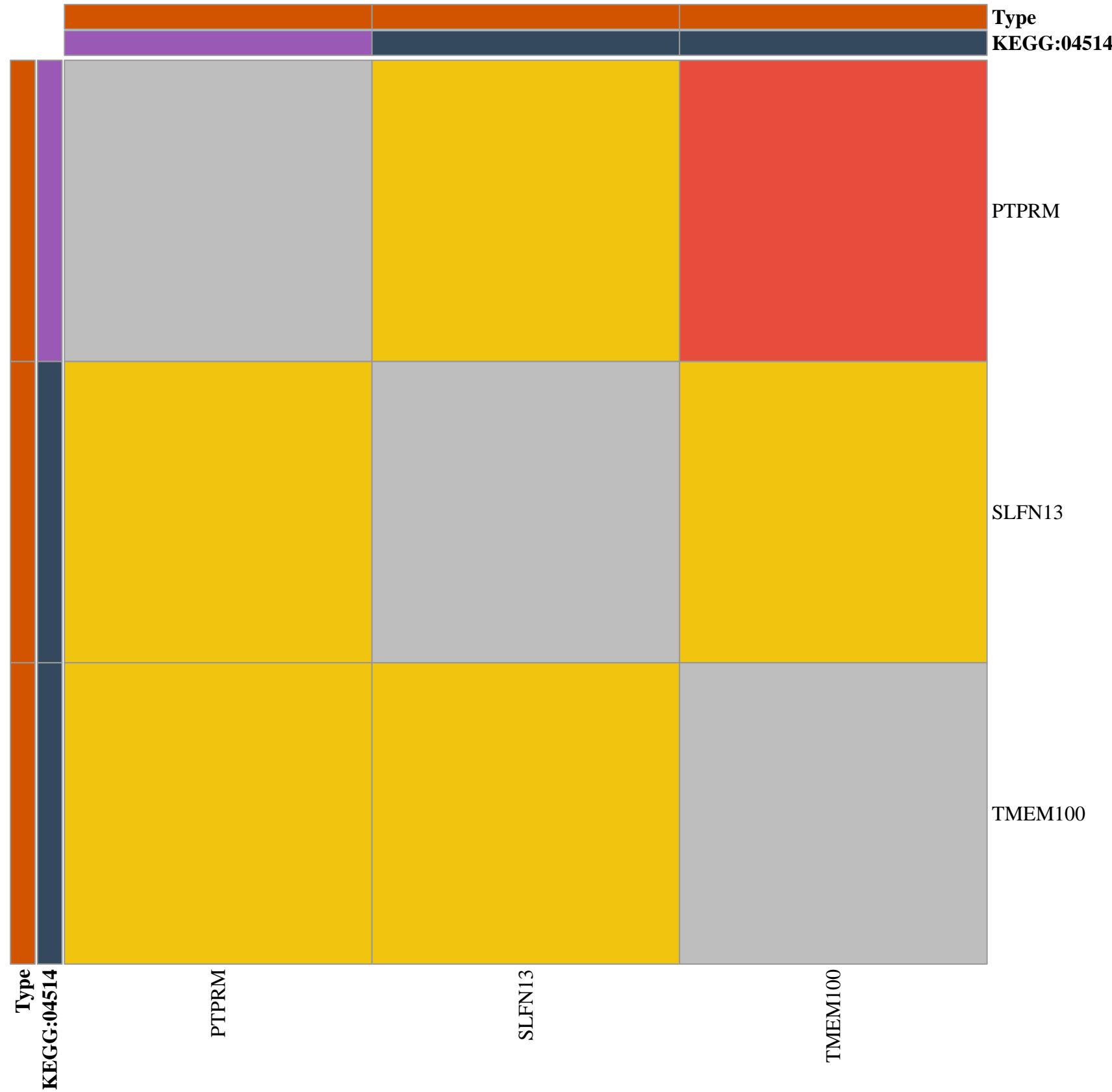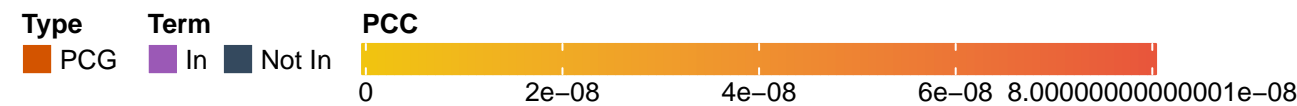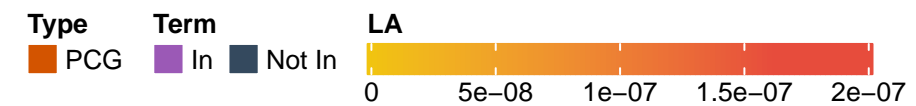

Supplement: Supplementary File 2 — Heatmap of PCC and LA values of modules enriched for KEGG:04514 in disease and normal states (top right: disease state, bottom left: normal state). [file Data_Sheet_2.PDF]

Module188

PCC

LA

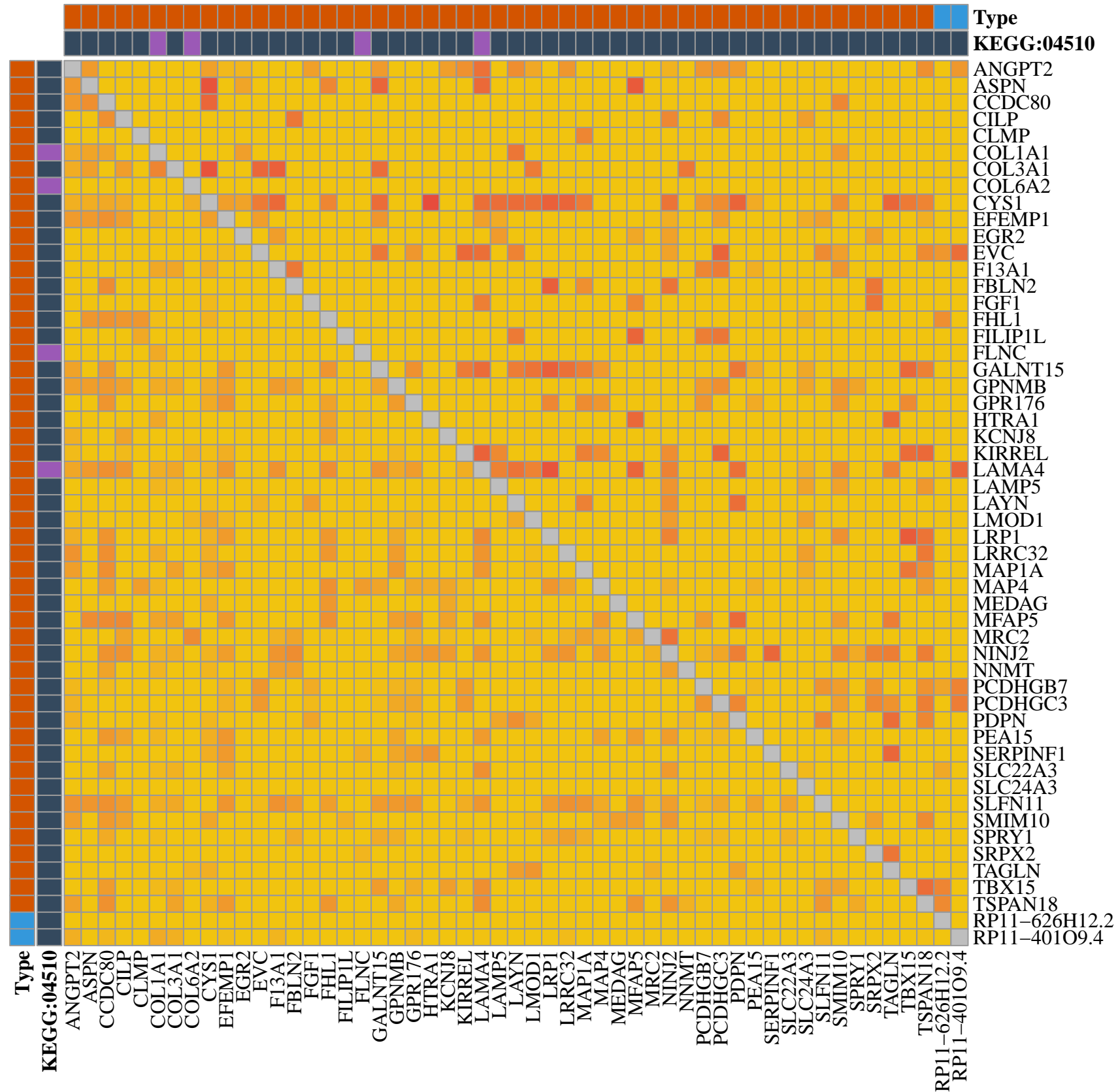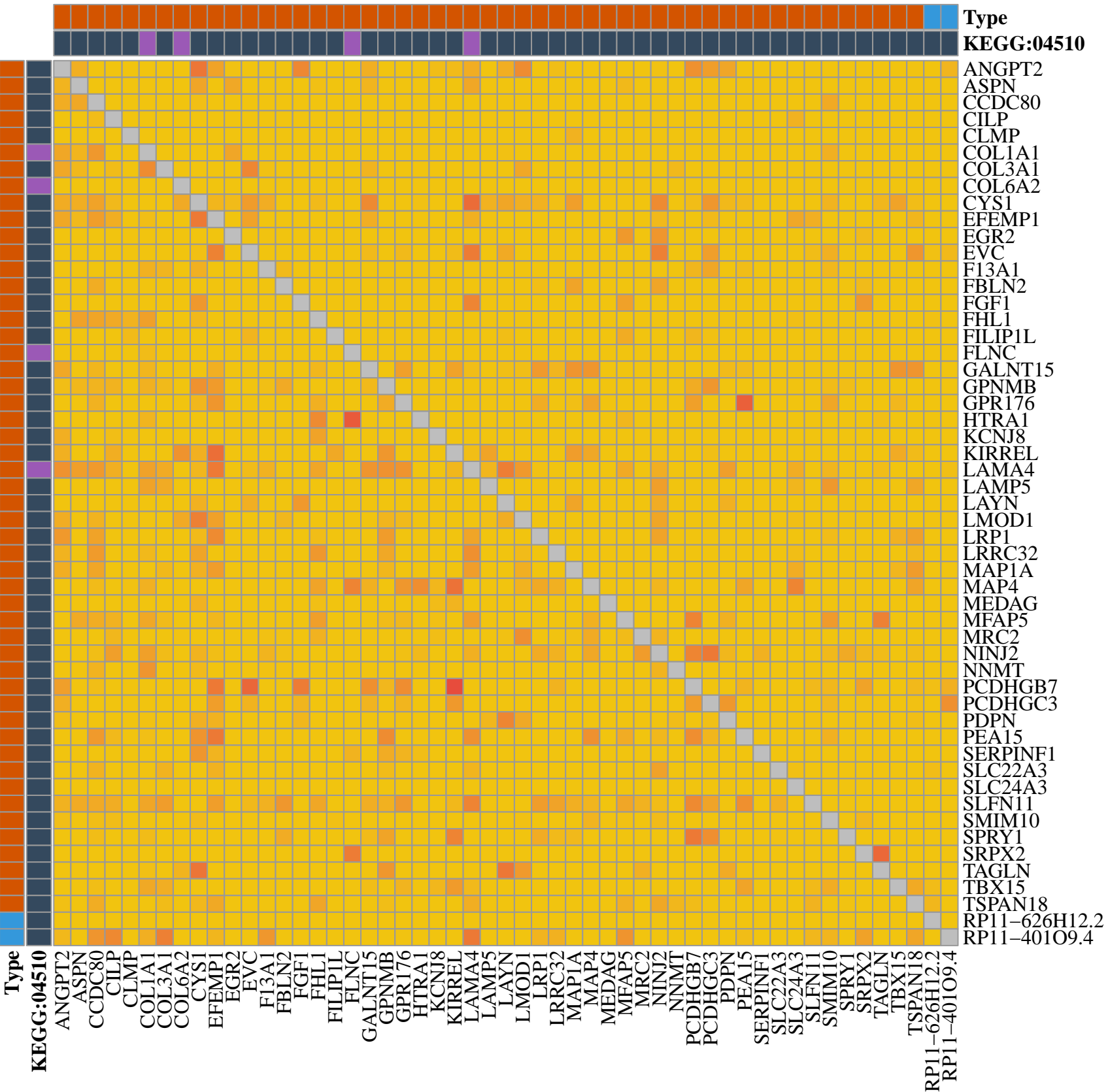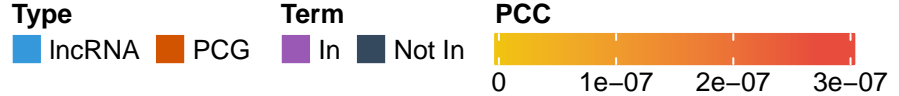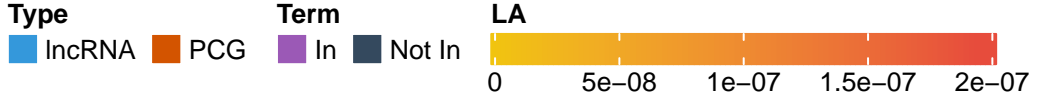

Module81

PCC

LA

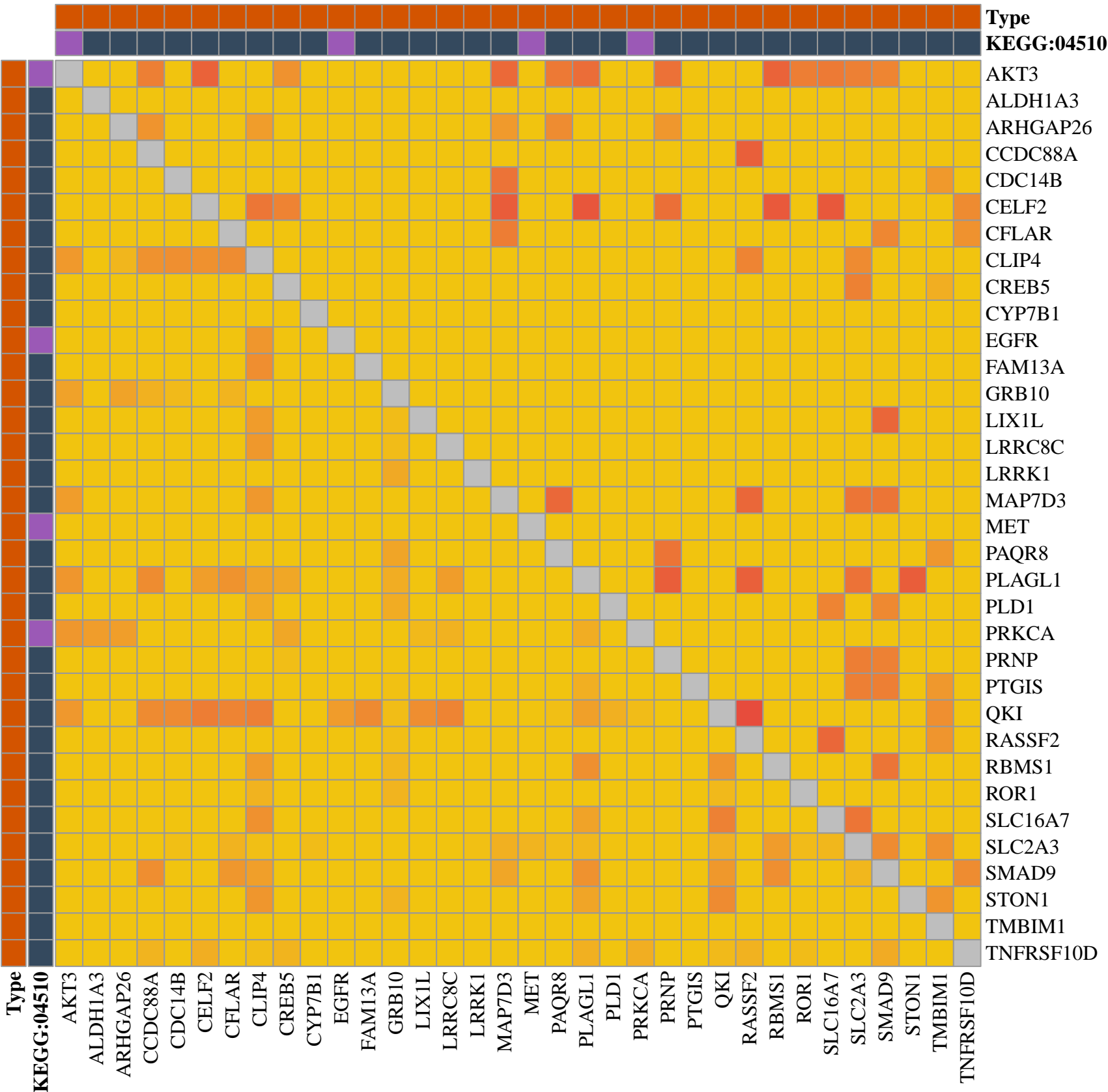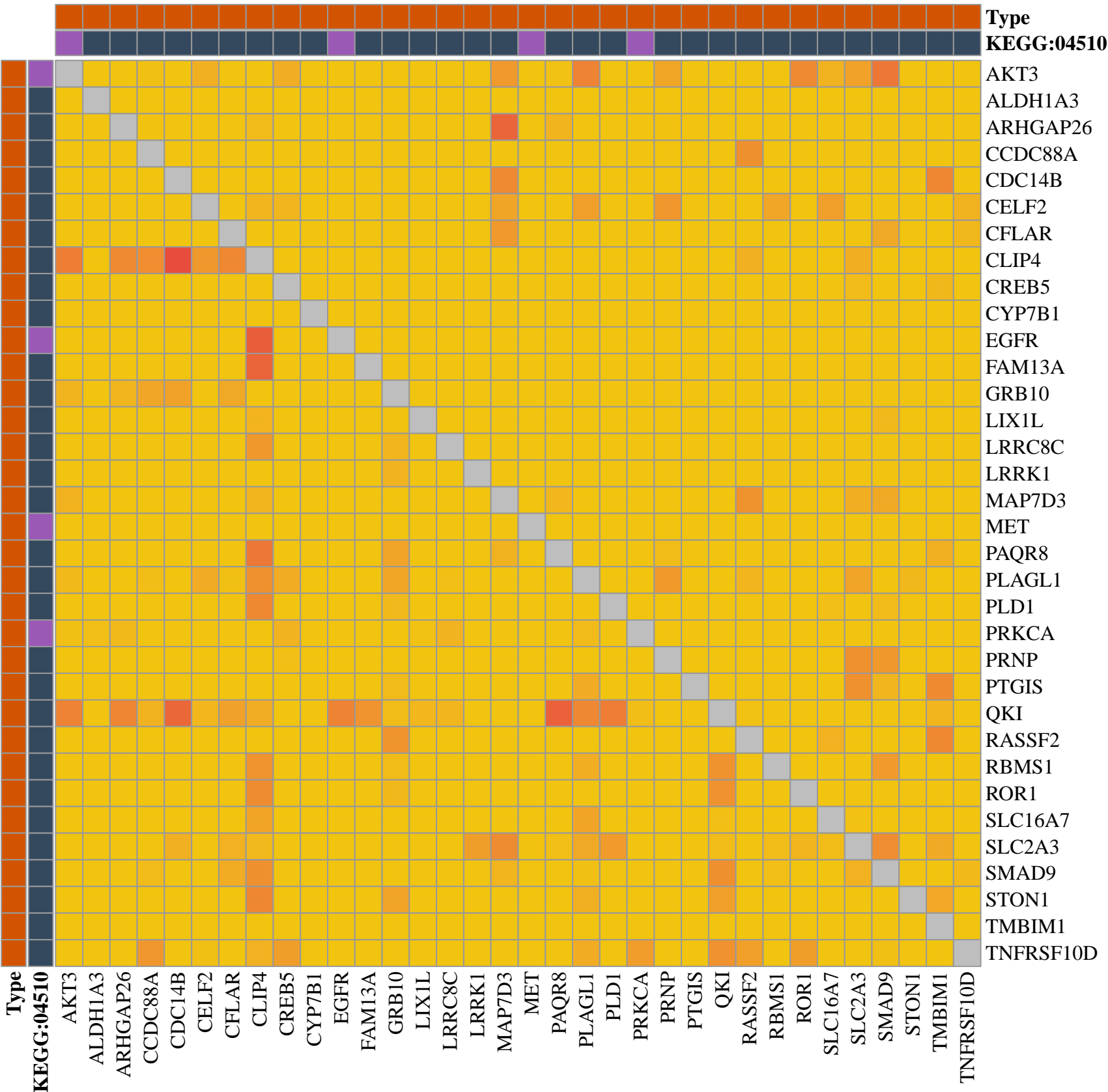

# Module31

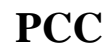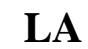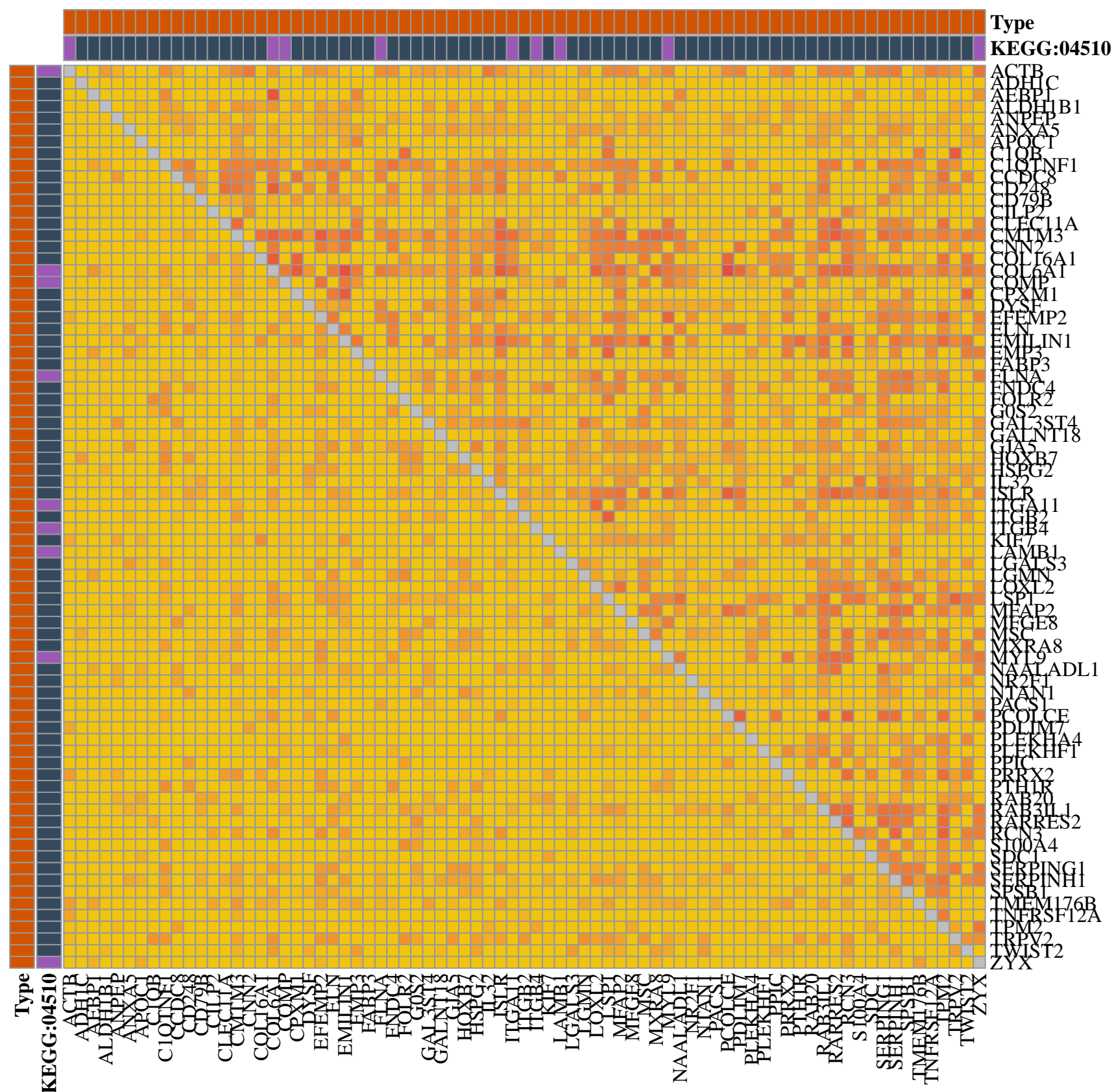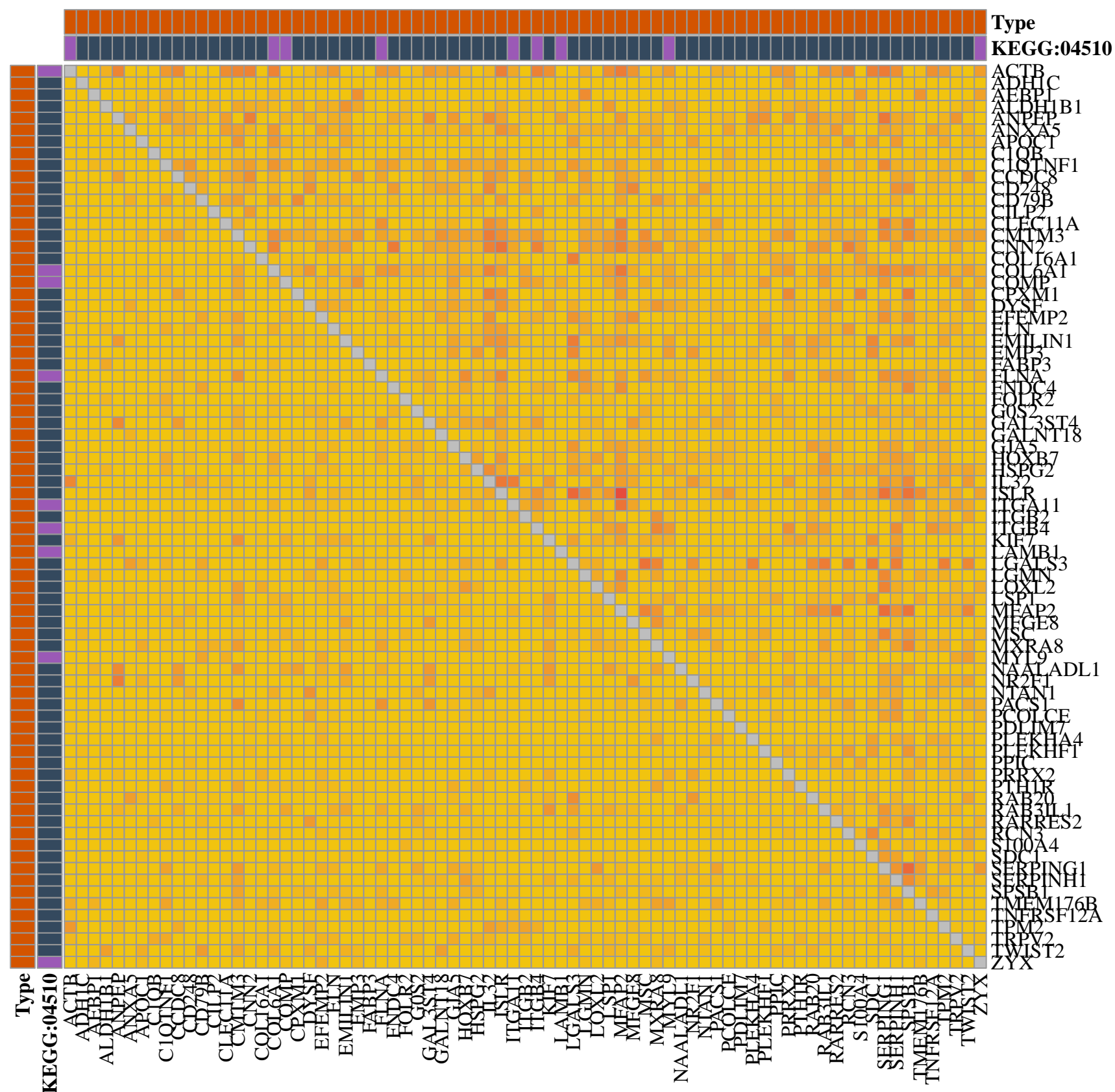

# Module277

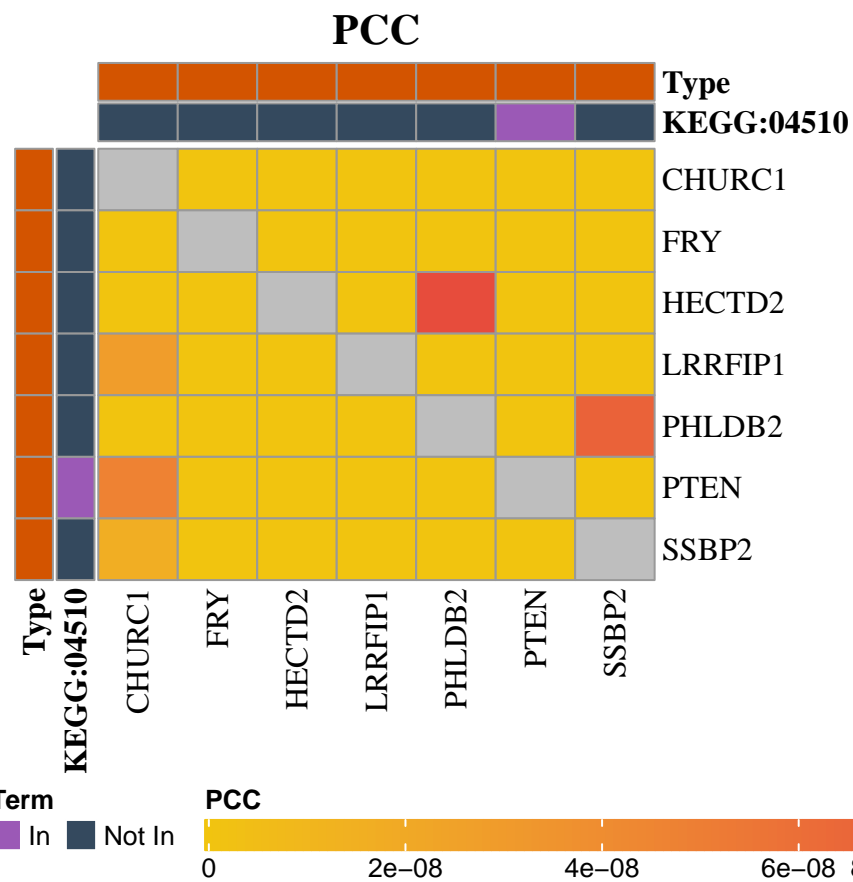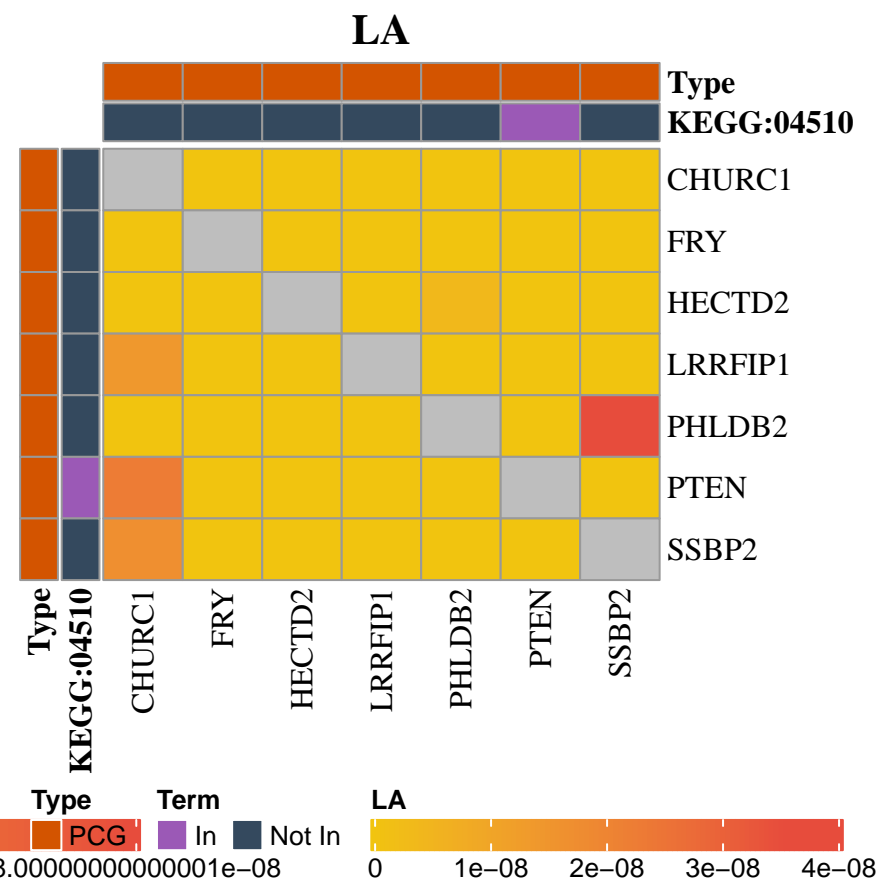

# Module178

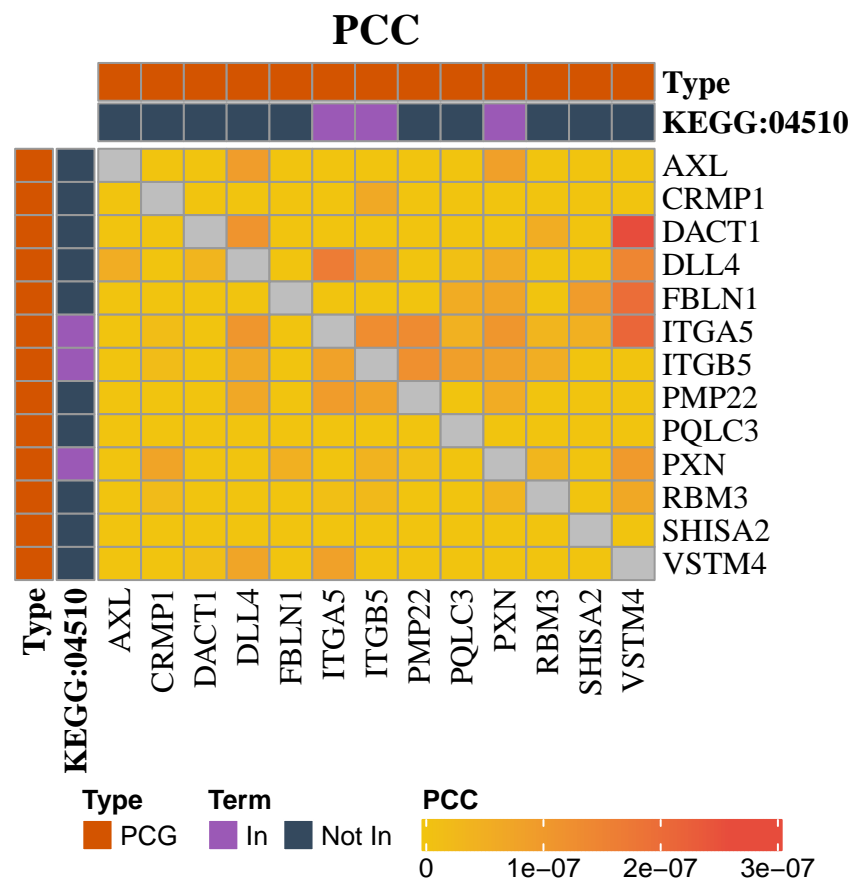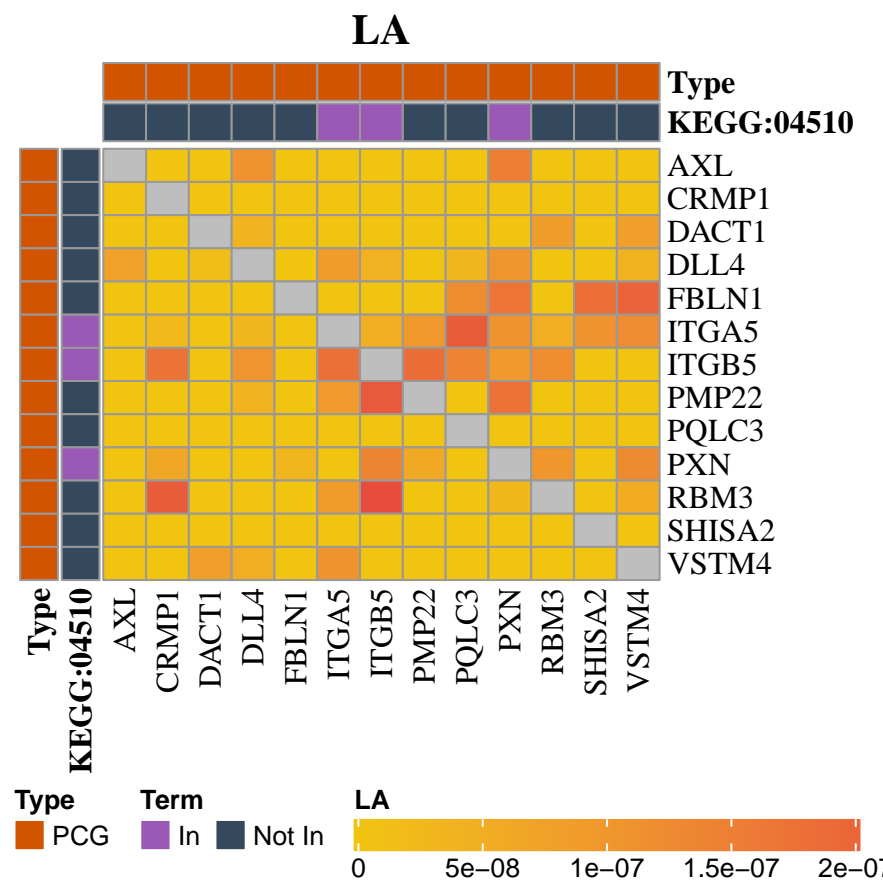

Supplement: Supplementary File 3 — Heatmap of PCC and LA values of modules enriched for KEGG:04510 in disease and normal states (top right: disease state, bottom left: normal state). [file Data_Sheet_3.PDF]

# Volcano Plot of Differential Expression Genes

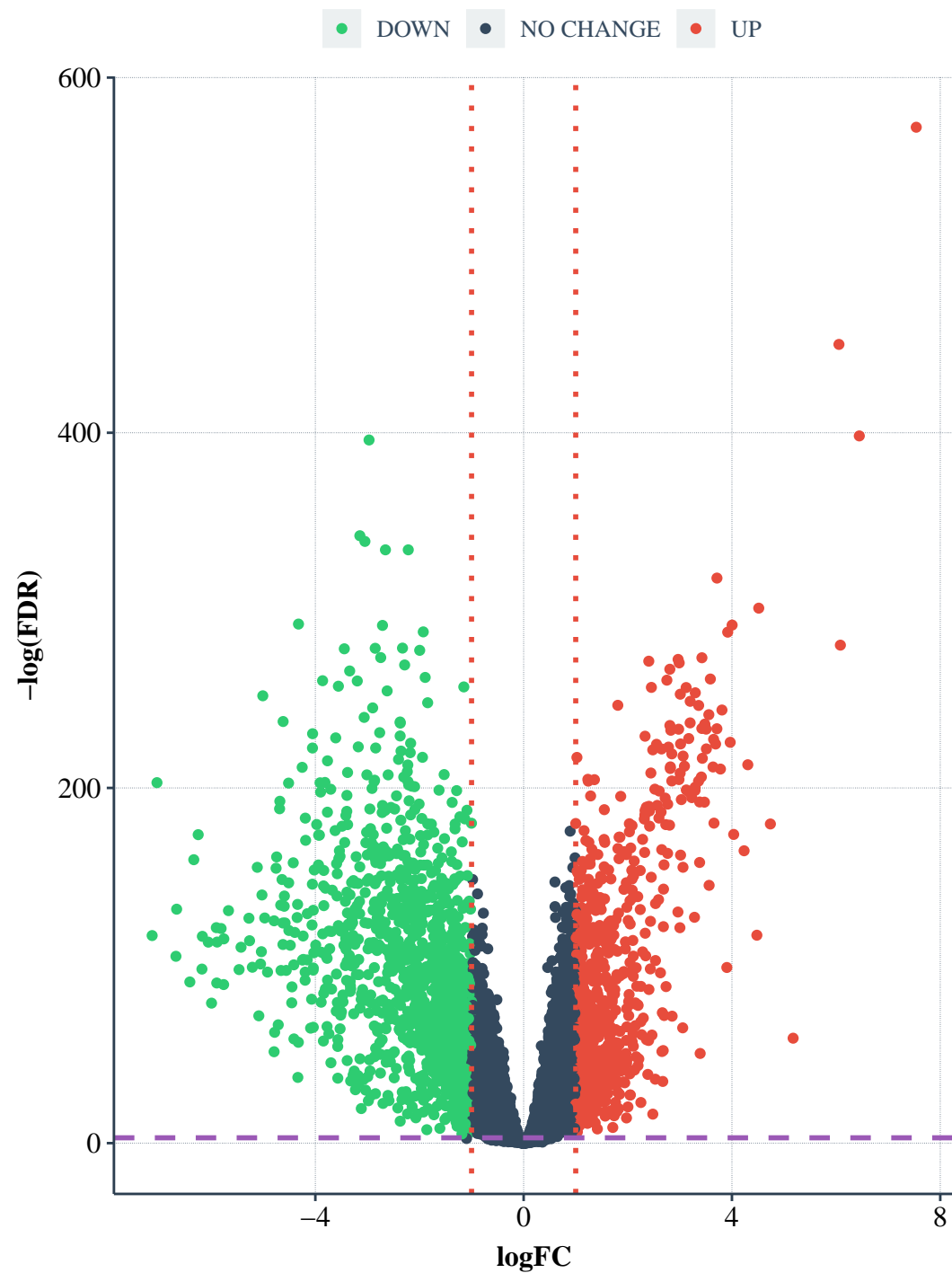

# Differentially Expressed Gene Profile

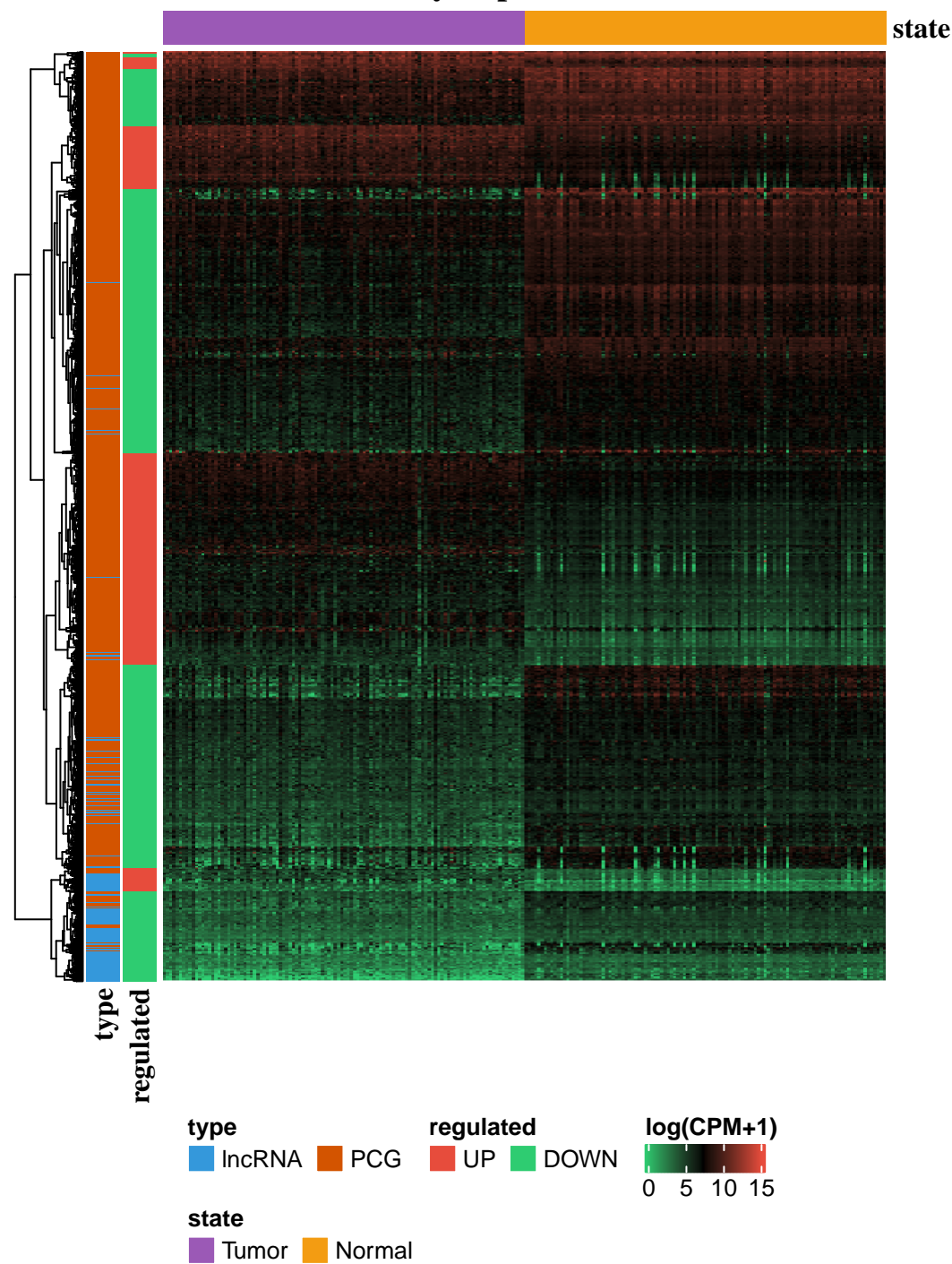

Supplement: Supplementary File 4 — Profiles of differentially expressed genes. [file Data_Sheet_4.PDF]

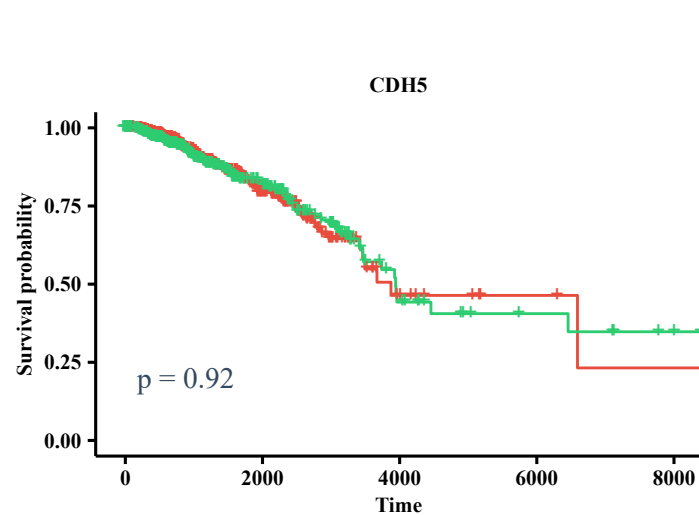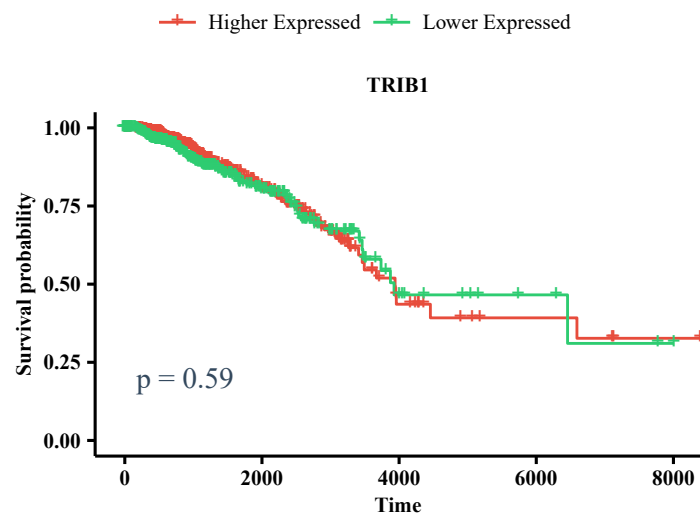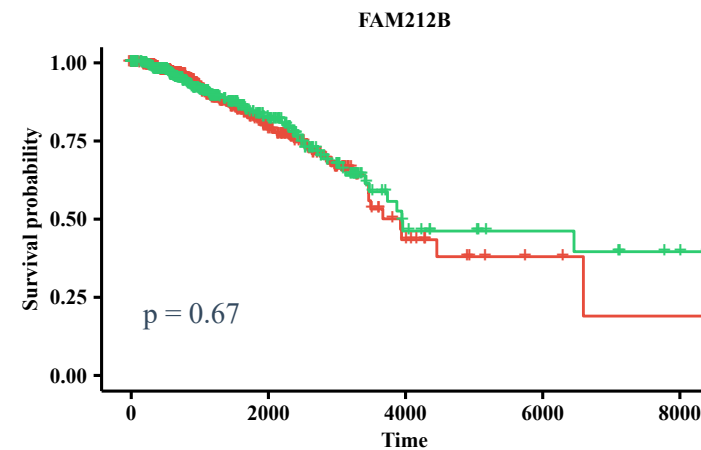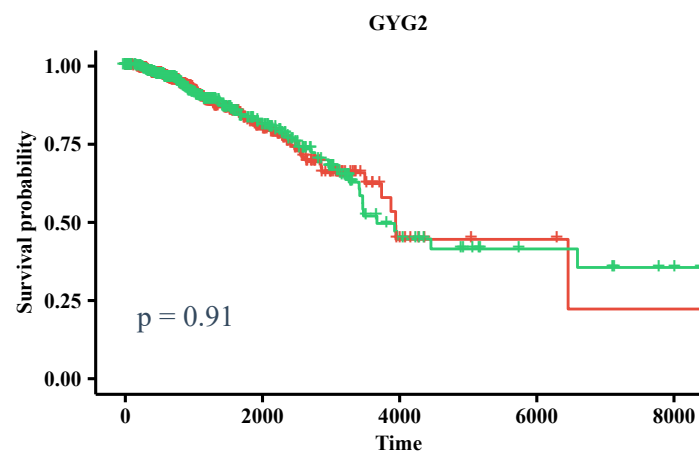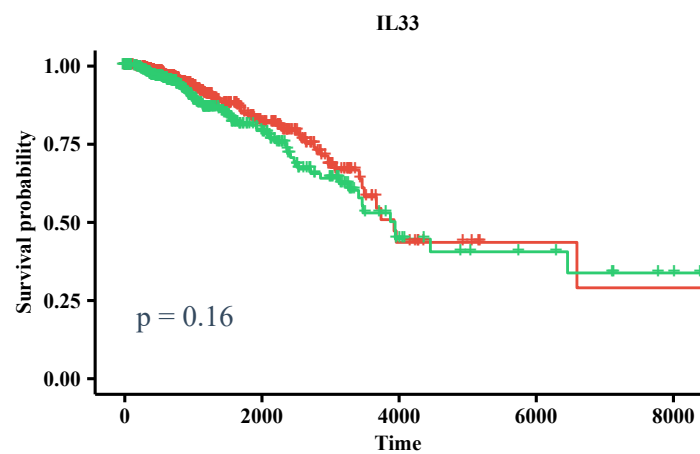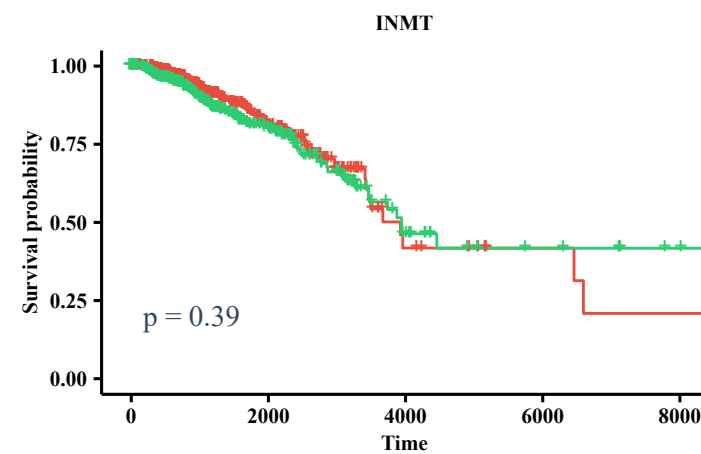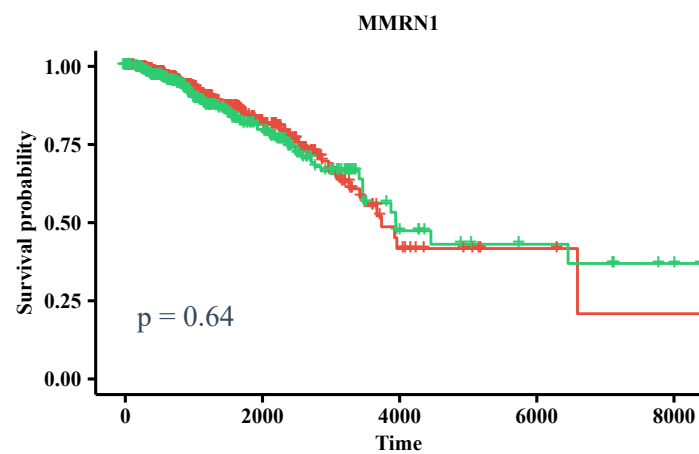

Supplement: Supplementary File 6 — Kaplan–Meier curves for individual genes in Figure 6C. [file Data_Sheet_6.PDF]

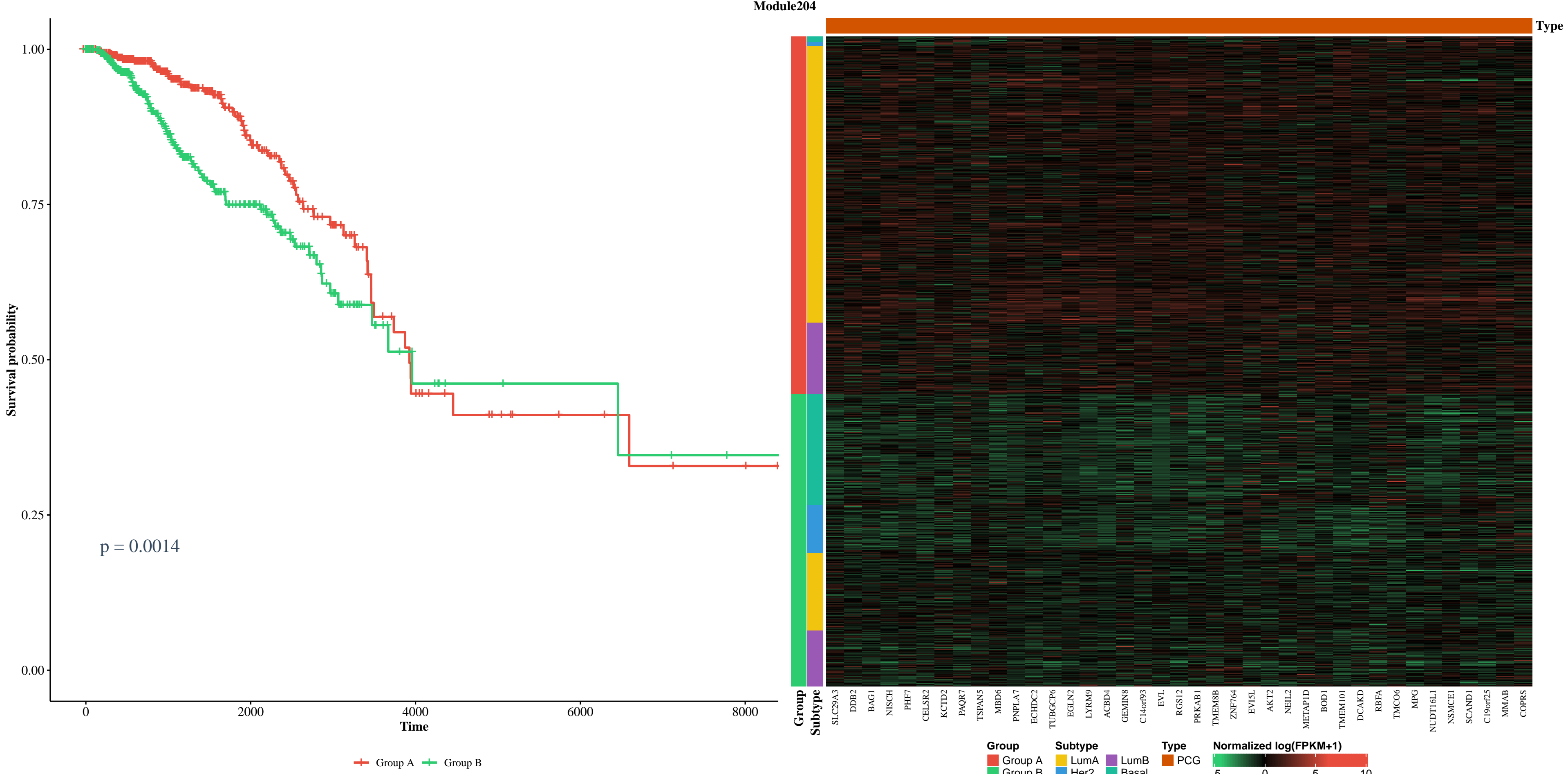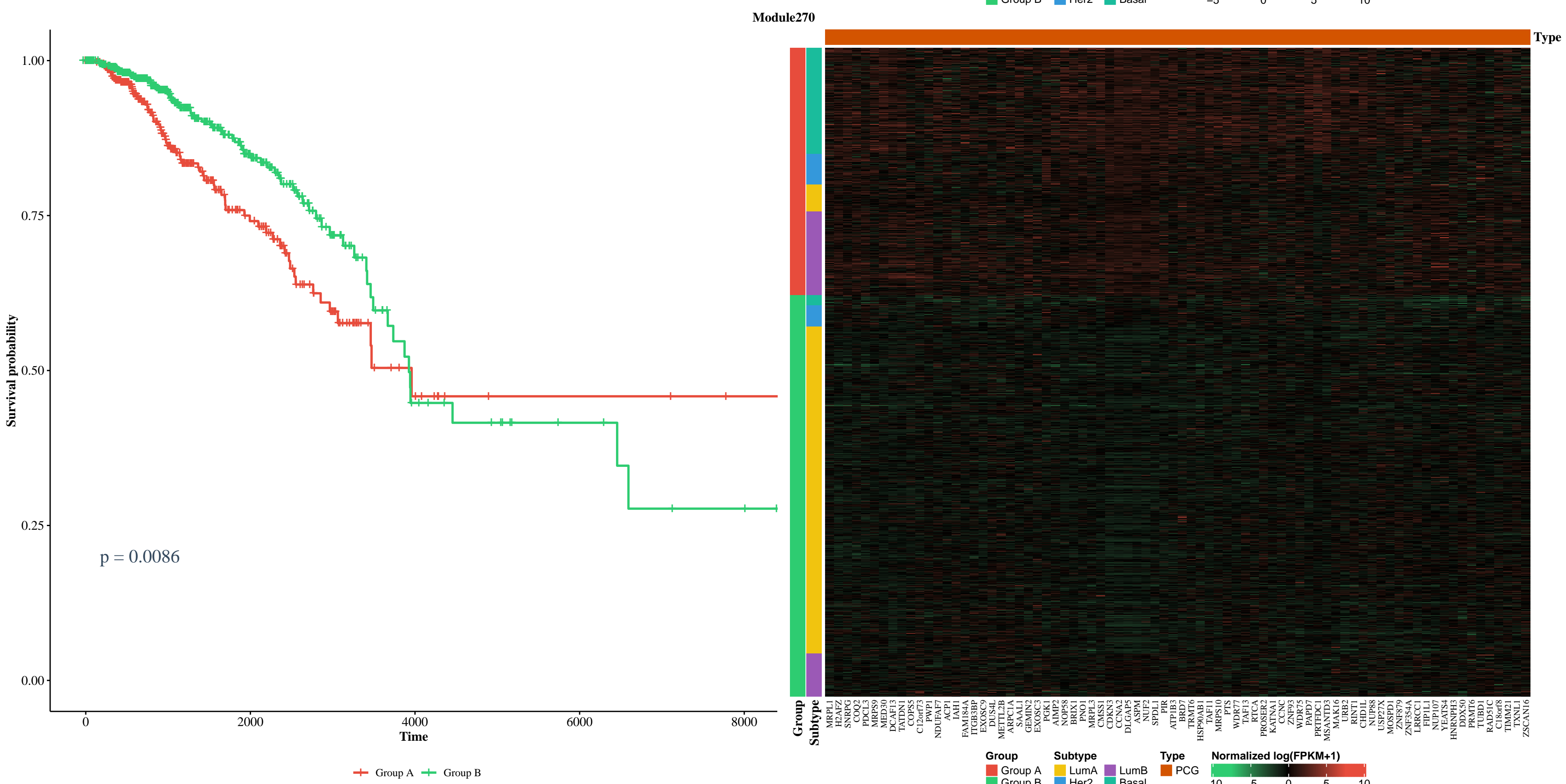

Supplement: Supplementary File 7 — Kaplan–Meier curves of prognosis marker modules (left) and the corresponding expression profiles (right). (Supplementary of Figure 7). [file Data_Sheet_7.PDF]

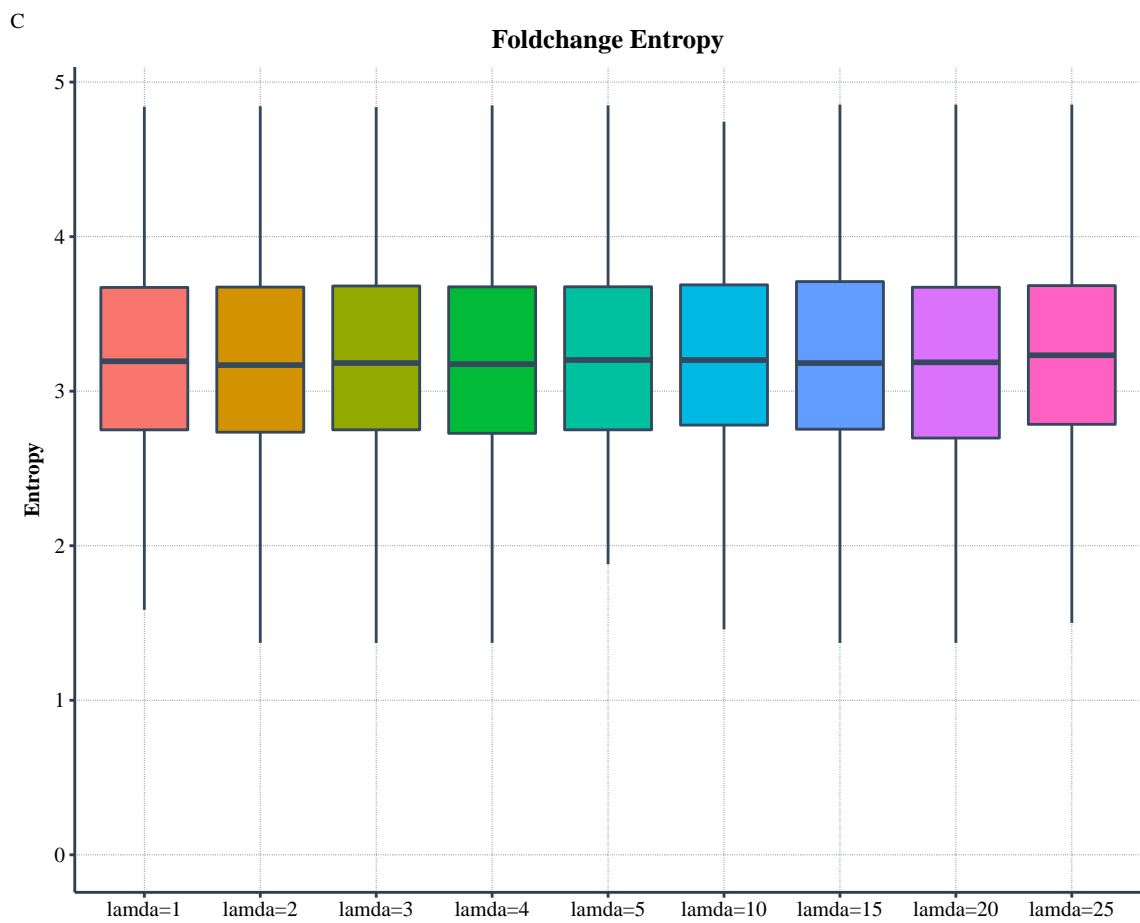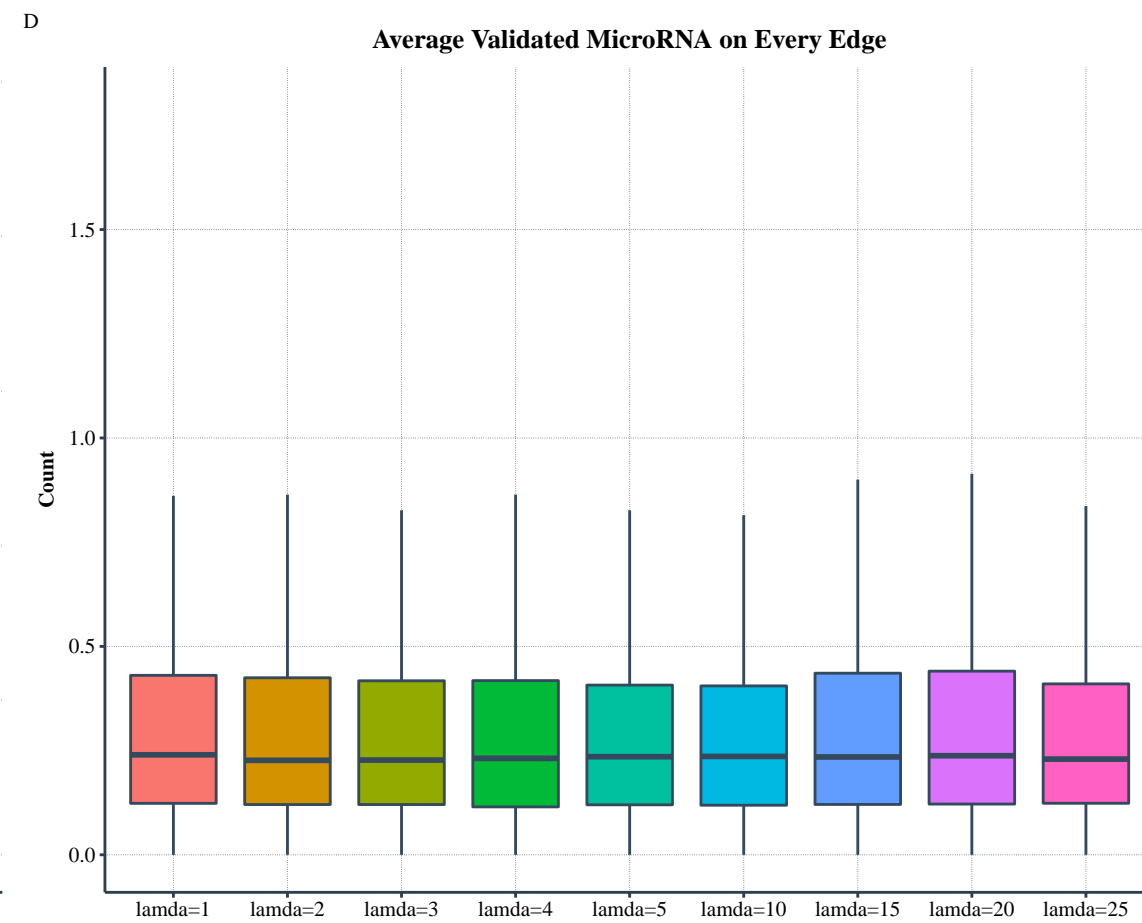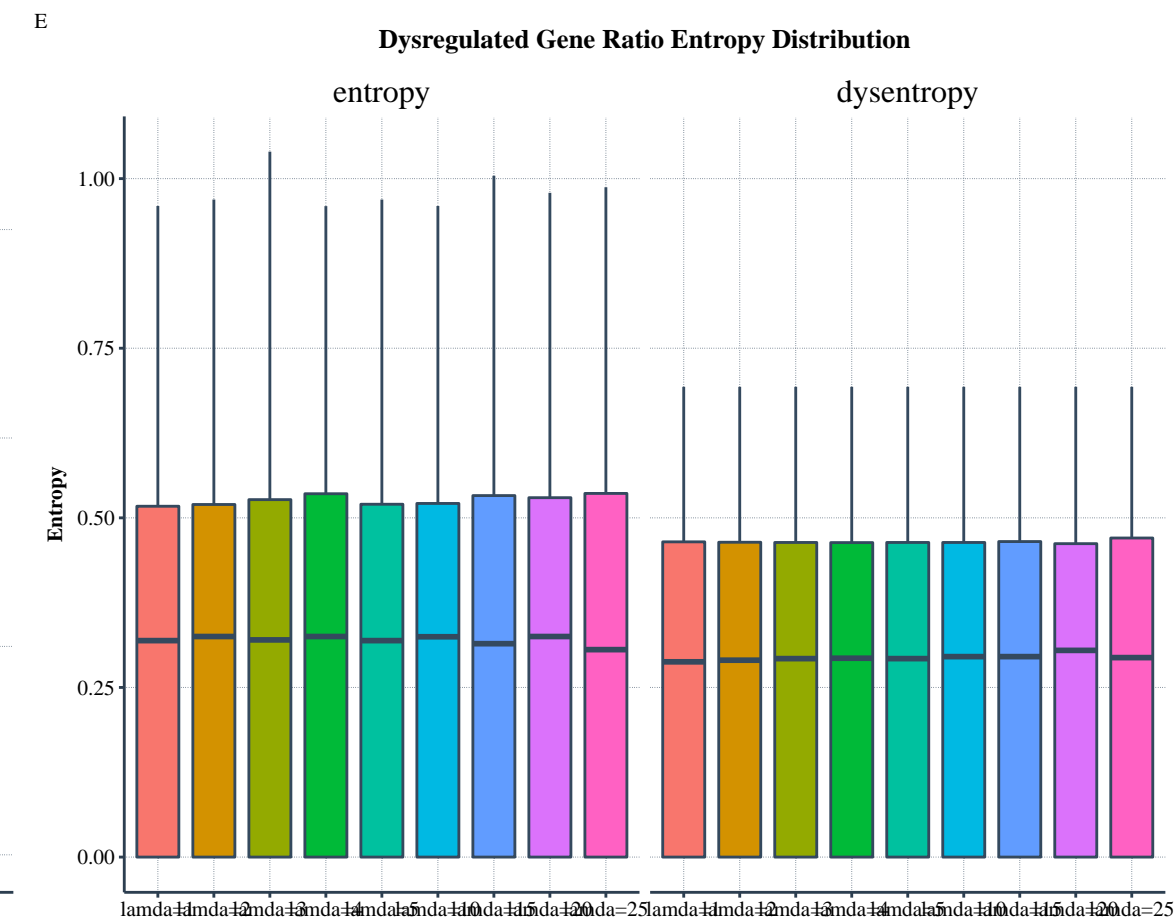

Supplement: Supplementary File 8 — Comparison of the results of different values for the parameter λLA. [file Data_Sheet_8.PDF]

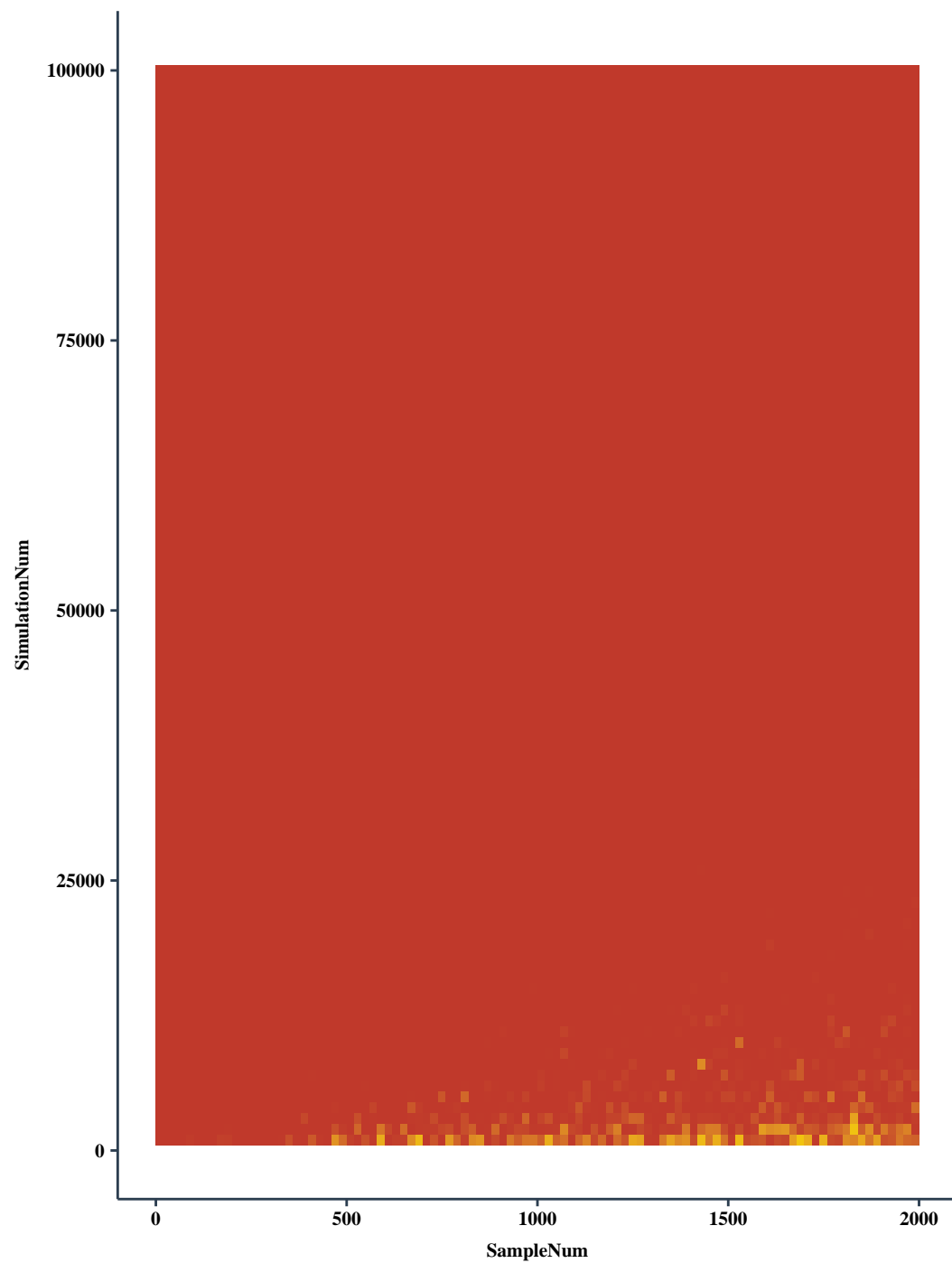

P-value 0.25 0.50 0.75 1.00

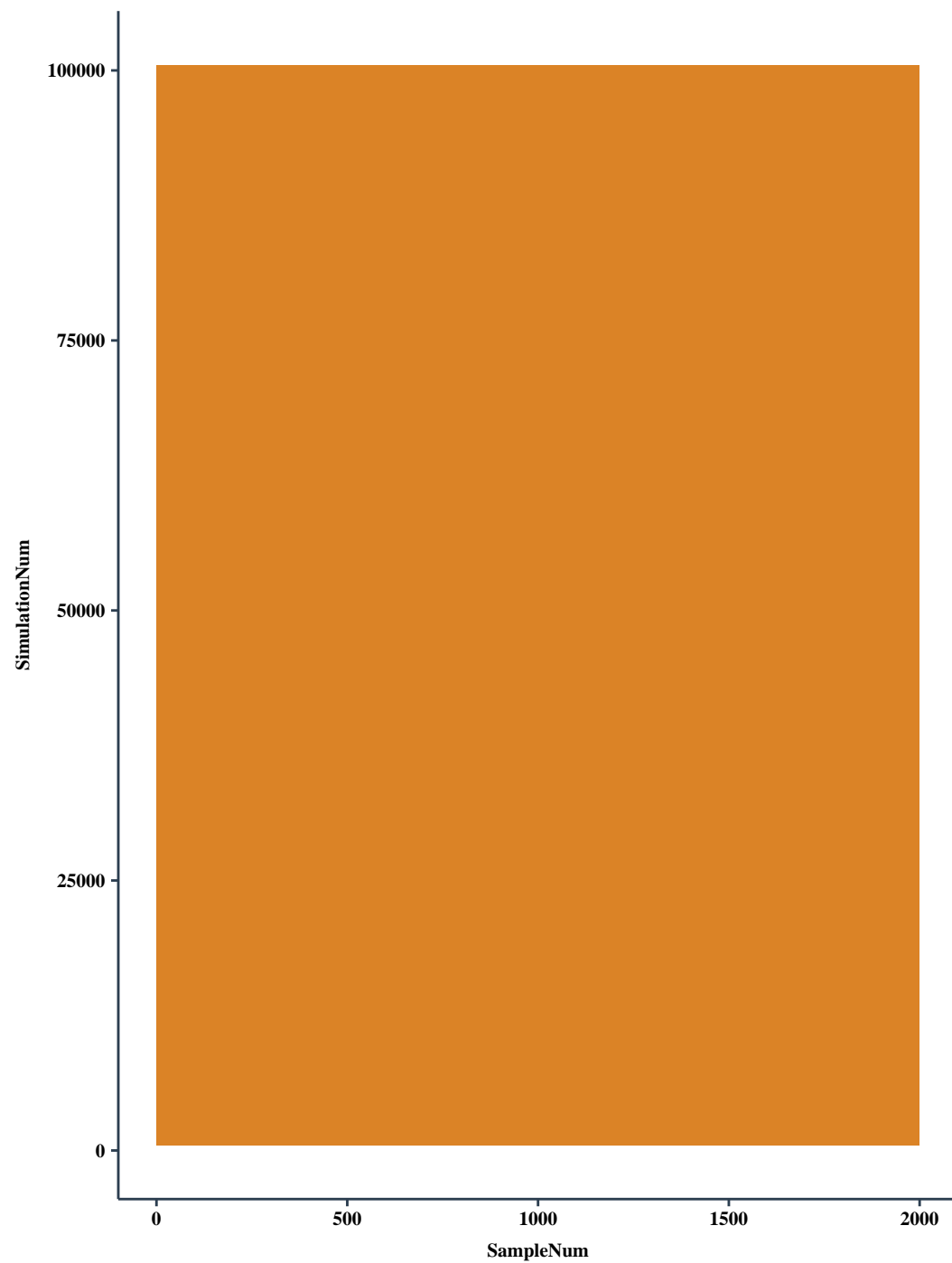

FDR 1

Supplement: Supplementary File 10 — The evaluation of Van der Waerden's method. [file Data_Sheet_10.PDF]
