# Supplementary material for: LAceModule: Identification of Competing Endogenous RNA Modules by Integrating Dynamic Correlation
Source: Front Genet. 2020 Mar 18;11:235. doi: 10.3389/fgene.2020.00235 (PMC7093494; doi:10.3389/fgene.2020.00235)
Supplement: Supplementary File 5 — BRCA-associated modules (Supplementary of Figure 5A). [file Data_Sheet_5.PDF]

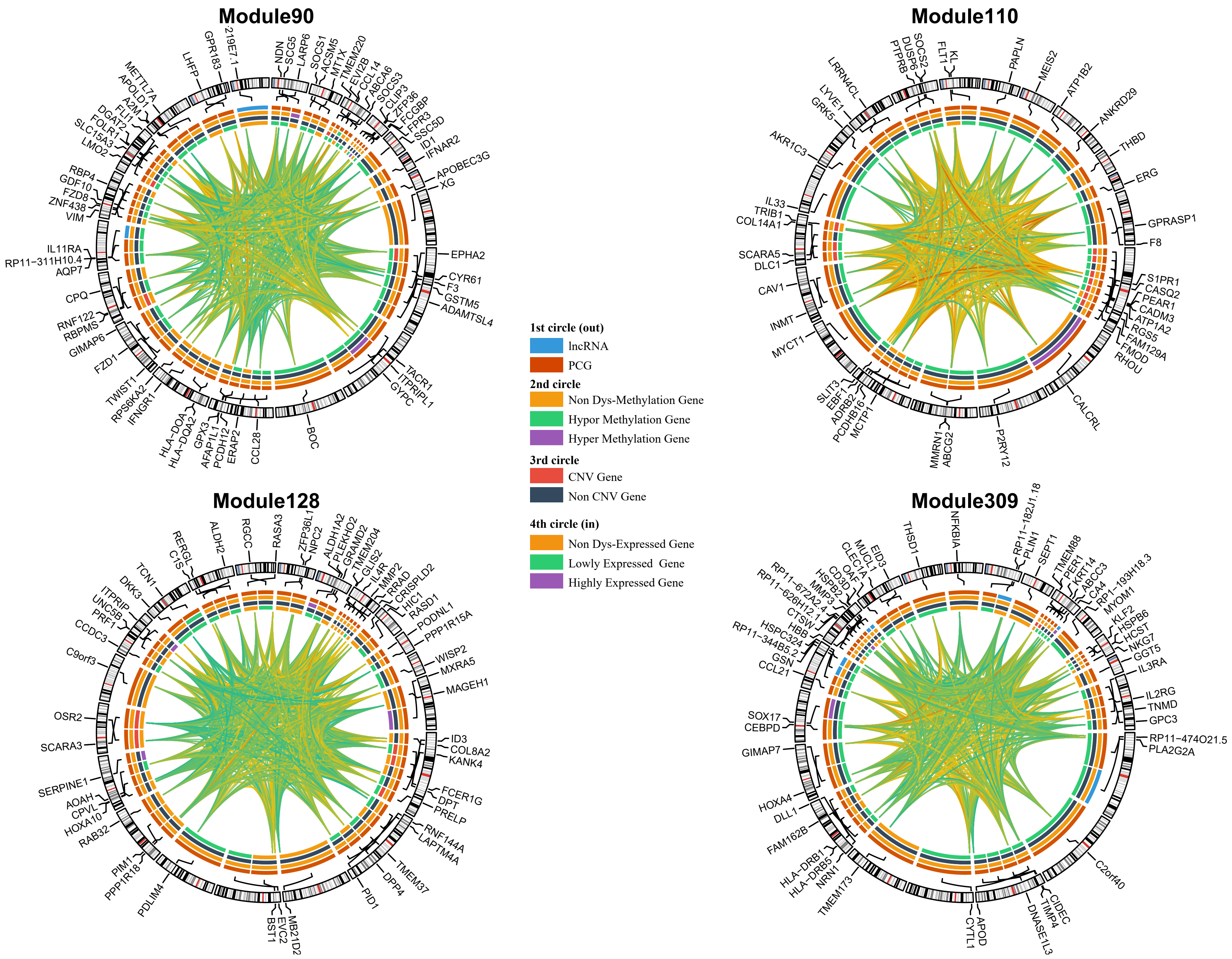
Module90
NDN
SCG5
LARP6
SOCS1
ACSM5
MT1X
TMEM220
EVI2B
CCL14
ABCA6
SOCS3
CLIP3
ZFP36
FCGBP
FPR3
SSC5D
ID1
IFNAR2
APOBEC3G
XG
EPHA2
CYR61
F3
GSTM5
ADAMTSL4
TACR1
ITPRIPL1
GYPC
BOC
CCL28
ERAP2
PCDH12
AFAP1L1
GPX3
HLA-DQA2
HLA-DOA
IFNGR1
RPS6KA2
TWIST1
FZD1
GIMAP6
RBPMS
RNF122
CPQ
AQP7
RP11-311H10.4
IL11RA
VIM
ZNF438
FZD8
GDF10
RBP4
LMO2
SLC15A3
FOLR1
DGAT2
FLI1
A2M
APOLD1
METTL7A
LHFP
GPR183
Module110
PTPRB
DUSP6
SOCS2
FLT1
KL
PAPLN
MEIS2
ATP1B2
ANKRD29
THBD
ERG
GPRASP1
F8
S1PR1
CASQ2
PEAR1
CADM3
ATP1A2
RGS5
FAM129A
FMOD
RHOU
CALCRL
P2RY12
ABCG2
MMRN1
MCTP1
PCDHB16
ADRB2
EBF1
SLIT3
MYCT1
INMT
CAV1
DLC1
SCARA5
COL14A1
TRIB1
IL33
AKR1C3
GRK5
LYVE1
LRRN4CL
1st circle (out)
lncRNA
PCG
2nd circle
Non Dys-Methylation Gene
Hypor Methylation Gene
Hyper Methylation Gene
3rd circle
CNV Gene
Non CNV Gene
4th circle (in)
Non Dys-Expressed Gene
Lowly Expressed Gene
Highly Expressed Gene
Module128
RERGL
C1S
ALDH2
RGCC
RASA3
ZFP36L1
NPC2
ALDH1A2
PLEKHO2
GRAMD2
TMEM204
GLIS2
IL4R
MMP2
RRAD
CRISPLD2
HIC1
RASD1
PODNL1
PPP1R15A
WISP2
MXRA5
MAGEH1
ID3
COL8A2
KANK4
FCER1G
DPT
PRELP
RNF144A
LAPTM4A
TMEM37
DPP4
PID1
MB21D2
EVC2
BST1
PDLIM4
PPP1R18
PIM1
RAB32
HOXA10
CPVL
AOAH
SERPINE1
SCARA3
OSR2
C9orf3
CCDC3
PRF1
UNC5B
ITPRIP
DKK3
TCN1
Module309
THSD1
NFKBIA
RP11-182J1.18
PLIN1
SEPT1
TMEM88
PER1
KRT14
ABCC3
CA4
RP1-193H18.3
MYOM1
KLF2
HSPB6
HCST
NKG7
GGT5
IL3RA
IL2RG
TNMD
GPC3
RP11-474O21.5
PLA2G2A
C2orf40
CIDEC
TIMP4
DNASE1L3
APOD
CYTL1
TMEM173
NRN1
HLA-DRB5
HLA-DRB1
FAM162B
DLL1
HOXA4
GIMAP7
CEBPD
SOX17
CCL21
GSN
RP11-344B5.2
HSPC324
HBB
CTSW
RP11-626H12.1
RP11-672A2.4
MMP3
HSPB2
CD3D
OAF
CLEC1A
MLIC1
EID3
