# Supplementary material for: LAceModule: Identification of Competing Endogenous RNA Modules by Integrating Dynamic Correlation
Source: Front Genet. 2020 Mar 18;11:235. doi: 10.3389/fgene.2020.00235 (PMC7093494; doi:10.3389/fgene.2020.00235)
Supplement: Supplementary File 9 — Patients groups by the expression of let-7 family and the gene set in Figure 6. [file Data_Sheet_9.PDF]

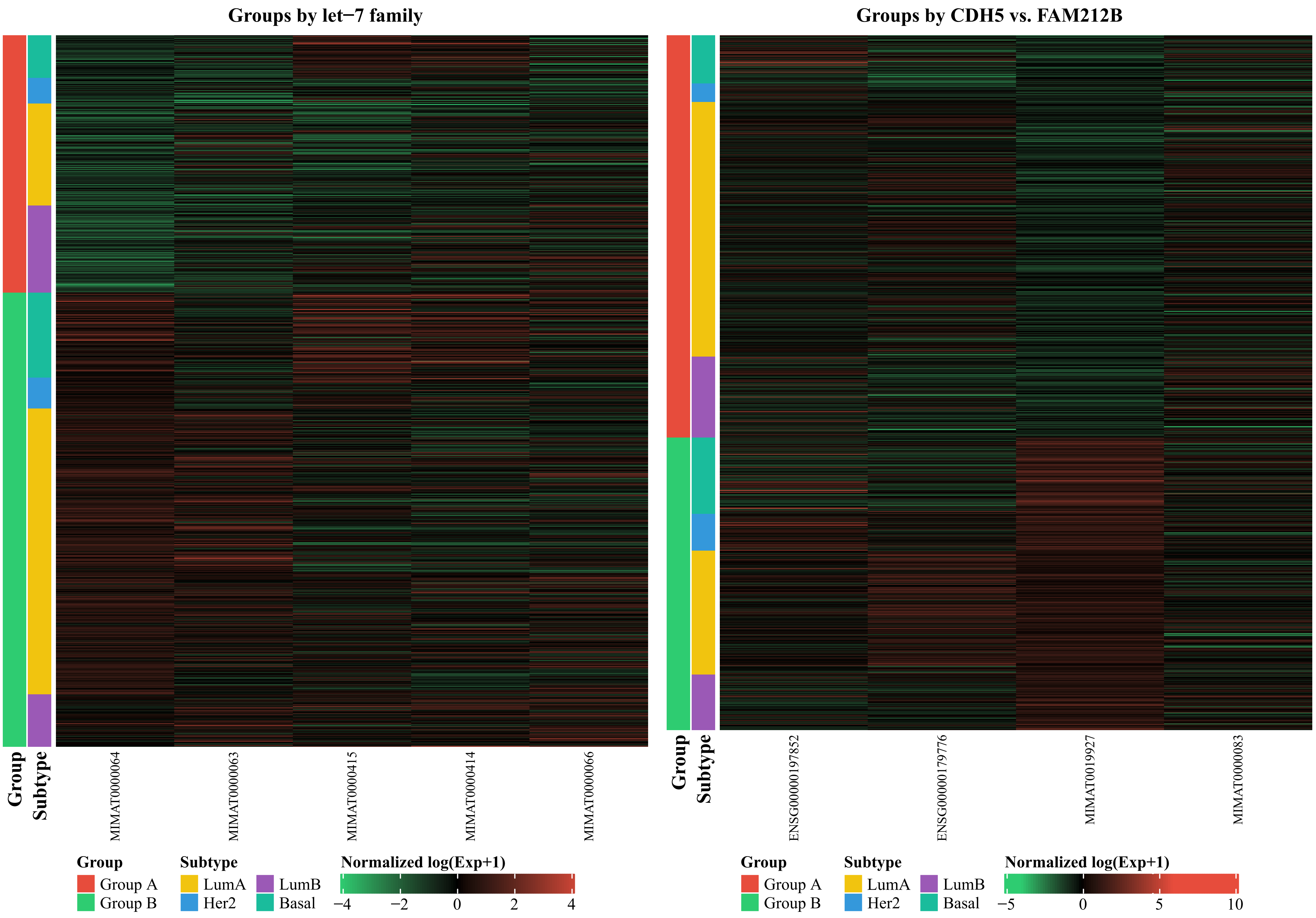
Groups by let−7 family
Groups by CDH5 vs. FAM212B
Group
Subtype
MIMAT0000064
MIMAT0000063
MIMAT0000415
MIMAT0000414
MIMAT0000066
ENSG00000197852
ENSG00000179776
MIMAT0019927
MIMAT0000083
Group
Group A
Group B
Subtype
LumA
LumB
Her2
Basal
Normalized log(Exp+1)
−4
−2
0
2
4
−5
0
5
10

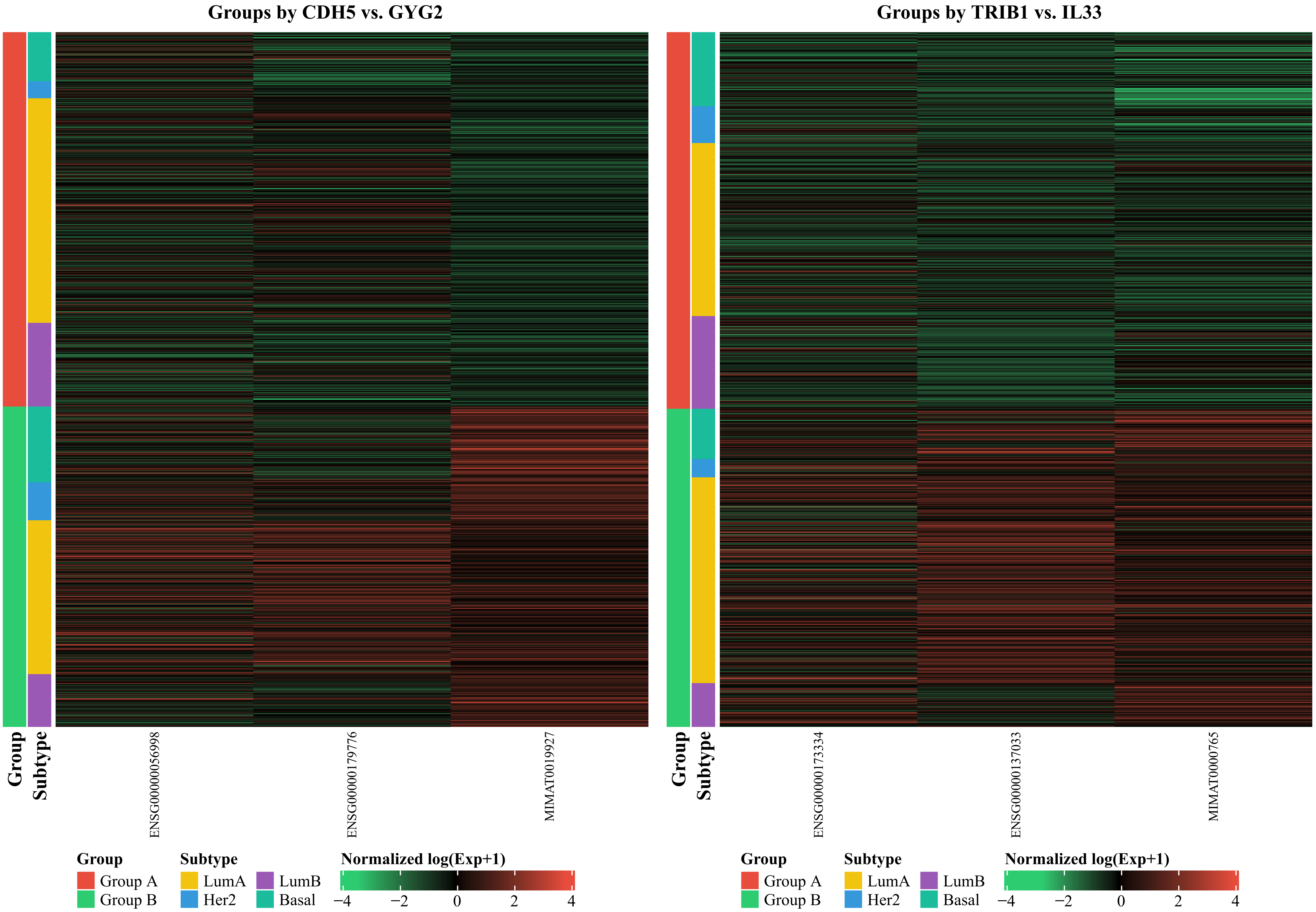

Groups by CDH5 vs. GYG2
Group
Subtype
ENSG00000056998
ENSG00000179776
MIMAT0019927
Group
Group A
Group B
Subtype
LumA
LumB
Her2
Basal
Normalized log(Exp+1)
−4
−2
0
2
4
Groups by TRIB1 vs. IL33
Group
Subtype
ENSG00000173334
ENSG00000137033
MIMAT0000765
Group
Group A
Group B
Subtype
LumA
LumB
Her2
Basal
Normalized log(Exp+1)
−4
−2
0
2
4

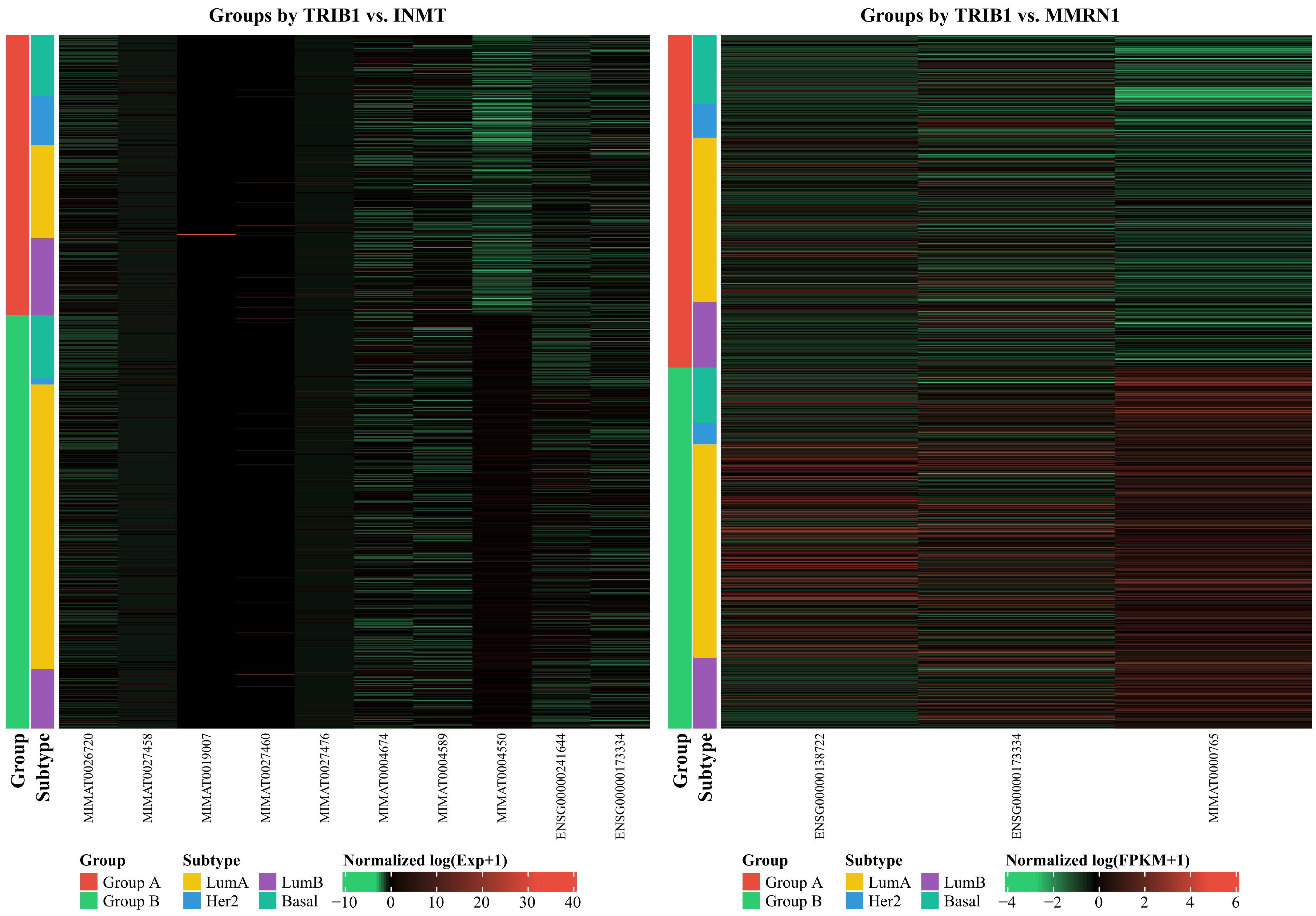
Groups by TRIB1 vs. INMT
Group
Subtype
MIMAT0026720
MIMAT0027458
MIMAT0019007
MIMAT0027460
MIMAT0027476
MIMAT0004674
MIMAT0004589
MIMAT0004550
ENSG00000241644
ENSG00000173334
Group
Group A
Group B
Subtype
LumA
Her2
LumB
Basal
Normalized log(Exp+1)
−10
0
10
20
30
40
Groups by TRIB1 vs. MMRN1
Group
Subtype
ENSG00000138722
ENSG00000173334
MIMAT0000765
Group
Group A
Group B
Subtype
LumA
Her2
LumB
Basal
Normalized log(FPKM+1)
−4
−2
0
2
4
6
